# Base-induced reversible H_2_ addition to a single Sn(ii) centre†
†Electronic supplementary information (ESI) available: Experimental and computational details. See DOI: 10.1039/c8sc03110j


**DOI:** 10.1039/c8sc03110j

**Published:** 2018-09-18

**Authors:** Roland C. Turnell-Ritson, Joshua S. Sapsford, Robert T. Cooper, Stella S. Lee, Tamás Földes, Patricia A. Hunt, Imre Pápai, Andrew E. Ashley

**Affiliations:** a Department of Chemistry , Imperial College London , London , SW7 2AZ , UK . Email: a.ashley@imperial.ac.uk; b Research Center for Natural Sciences , Hungarian Academy of Sciences , Magyar tudósok körútja 2 , H-1117 Budapest , Hungary . Email: papai.imre@ttk.mta.hu

## Abstract

A range of amines catalyse the oxidative addition (OA) of H_2_ to [(Me_3_Si)_2_CH]_2_Sn (**1**), forming [(Me_3_Si)_2_CH]_2_SnH_2_ (**2**). Experimental and computational studies point to ‘frustrated Lewis pair’ mechanisms in which **1** acts as a Lewis acid and involve unusual late transition states; this is supported by the observation of a kinetic isotope effect  for Et_3_N. When DBU is used the energetics of H_2_ activation are altered, allowing an equilibrium between **1**, **2** and adduct [**1**·DBU] to be established, thus demonstrating reversible oxidative addition/reductive elimination (RE) of H_2_ at a single main group centre.

## Abstract 2

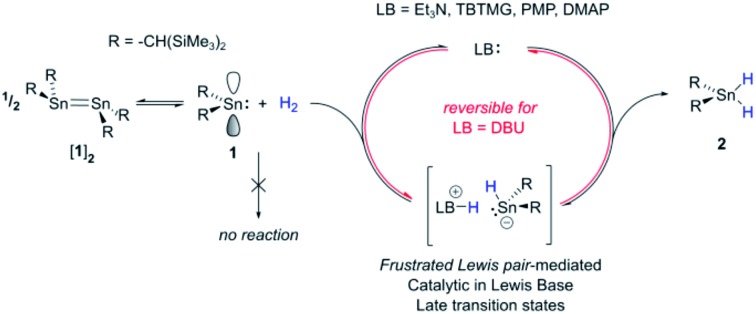
A ‘frustrated Lewis pair’-type mechanism allows the first observation of reversible H_2_ addition to a single-site main-group complex.

## Graphical abstract

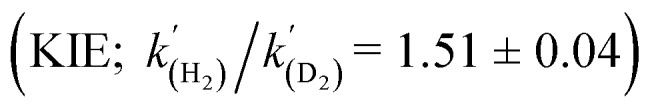

## Introduction

**Fig. 1:**
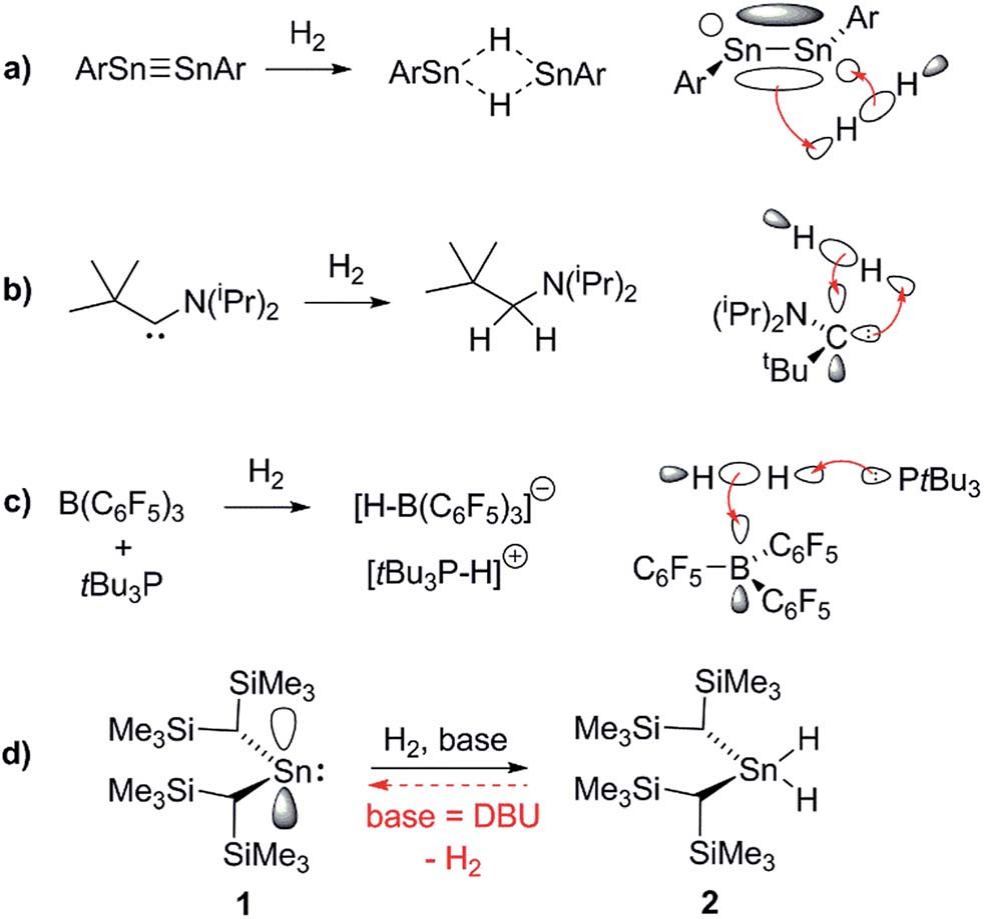
Representative orbital interactions between H_2_ and main group compounds: (a) unsaturated E–E compounds *e.g.* distannynes (Ar = C_6_H_2_-2,6-(C_6_H_3_-2,6-^i^Pr_2_)_2_-4-X; X = H, SiMe_3_, F); for X = H, the reaction is reversible at 80 °C;5a,^13^ (b) single site low-valent centres *e.g.* carbenes;6*a* (c) sterically hindered LAs and LBs (FLPs); (d) this work.

In the past decade there has been significant interest in transition metal (TM) free systems which activate H_2_.^1^ Two main strategies have emerged to facilitate this reactivity: the use of low-valent main group (MG) compounds,^2^ and so-called ‘frustrated Lewis pairs’ (FLPs).^3^ In both cases, reactivity arises from simultaneously having access to a high-lying HOMO and low-lying LUMO (Fig. 1). Various low-valent MG compounds containing multiple E–E bonds (E = Al, Si, Ga, Ge, Sn),^4^,^5^ or single-site low-valent centres such as carbenes and heavier tetrylene analogues, have been shown to react with H_2_.^6^ The scope of Lewis bases (LBs) and, to a lesser extent, Lewis acids (LAs), which can be used in H_2_-activating FLPs has expanded to include a number of elements from across the periodic table. This is principally due to the readily tuneable steric and electronic profiles of the individual LA and LB sites.^7^–^9^ Many FLP systems display reversible H_2_ cleavage, which has facilitated their rapid expansion into the field of catalytic hydrogenation.^10^ The same is not true for low-valent MG compounds; examples of reversible H_2_ activation are very rare and limited to antiaromatic boracycles,^11^ a phosphorus-based singlet biradicaloid,^12^ and only one low-valent group 14 compound: a dinuclear Sn(i) distannyne.^13^ The design of single-site MG systems which are ergoneutral for H_2_ activation requires fine-tuning of thermodynamic (*e.g.* weak E–H bond strengths promoting an accessible formal E^*n*+2^/E^*n*^ couple) and kinetic factors, both of which are constrained to a mononuclear species, and is hence especially challenging.

The ability of L_2_Sn(ii) compounds to undergo OA has been inversely correlated with the size of the singlet–triplet (HOMO–LUMO) gap, which may be diminished through the use of extremely strong σ-donor ligands. Aldridge *et al.* have employed a bis(boryl)tin(ii) system to achieve the only example of direct OA of H_2_ to a mononuclear Sn(ii) centre, irreversibly forming the Sn(iv) dihydride; boryl ligands are even stronger σ-donors than hydride or alkyl ligands, permitting a successful reaction outcome.6*d*

Conversely, the irreversible base-induced RE of H_2_ from organostannanes is well-known.^14^ Wesemann and others have studied RE from ArSnH_3_ and [(Me_3_Si)_2_CH]SnH_3_ compounds to yield various mononuclear Sn and Sn–Sn bound species (Ar = terphenyl).^15^ Nevertheless, there has yet to be a report of reversible OA and RE occurring on a single Sn(ii) scaffold. Lappert's stannylene [(Me_3_Si)_2_CH]_2_Sn (**1**), which can act as both Lewis acid (LA) and base (LB), is a paradigmatic system for investigating OA to low-valent MG centres, yet to date its reactivity with H_2_ has been unexplored.^16^ Herein we report the use of FLP methodology to promote formal OA of H_2_ to this simple dialkylstannylene. Furthermore we document the first example of reversible H_2_ addition to a single-site MG complex, which accesses an FLP *via* reversible dissociation of a classical **1**·LB adduct; formation of the latter renders OA of H_2_ to **1** energetically less favourable, enabling RE to occur from the Sn(iv) dihydride and reform **1**, which is in equilibrium with **1**·LB.^17^

## Results and discussion

**Fig. 2:**
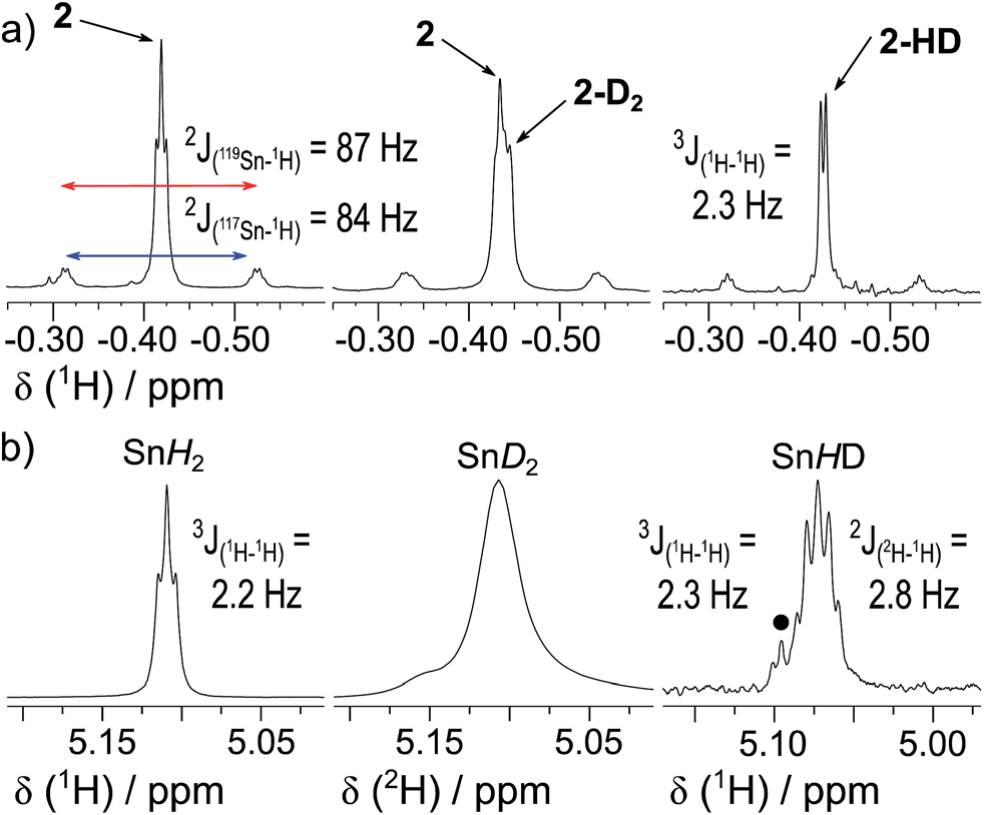
^1^H/^2^H NMR spectra from the reaction of **1** and 20 mol% Et_3_N: (a) C*H*(SiMe_3_)_2_ region under H_2_ (left); H_2_/D_2_ (1 : 1) (middle); HD (right). (b) Sn*H* region under H_2_ (left); H_2_/D_2_ (1 : 1) (middle; ^2^H NMR); HD (right). [black circle] denotes trace formation of **2** from H_2_ in commercial HD gas.

**1** is in a rapid solution-phase equilibrium with its dimer [**1**]_2_, which has been crystallographically characterised and contains a formal Sn=Sn double bond.^18^ When a *d*_8_-toluene solution of **1**/[**1**]_2_ was placed under an atmosphere of H_2_ (4 bar) in a sealed NMR tube, no change was observed in the ^1^H NMR spectrum, even after prolonged periods (>48 h), confirming that neither **1** nor [**1**]_2_ can react with H_2_ alone. Separately, addition of Et_3_N (20 mol%) to a solution of **1** resulted in no perturbation of their ^1^H NMR resonances, suggesting no interaction between the components; *i.e.* the formation of an FLP.^19^ Placing this new mixture under H_2_ (4 bar, RT) resulted in the solution turning from deep red to colourless over the course of 24 h, with the ^1^H NMR spectrum revealing complete consumption of **1** and a new Sn–H triplet resonance at *δ* = 5.10 ppm [^3^*J*(^1^H–^1^H) = 2.2 Hz] with attendant satellites [^1^*J*(^117^Sn–^1^H) = 1704 Hz; ^1^*J*(^119^Sn–^1^H) = 1784 Hz], in addition to signals for the Si(C*H*_3_)_3_ and methine protons [*δ*/ppm = 0.17 (s) and –0.42 (t, ^3^*J*(^1^H–^1^H) = 2.2 Hz), respectively] (see Fig. 2). ^119^Sn NMR spectroscopy showed only a triplet of triplets at –196 ppm [^1^*J*(^119^Sn–^1^H) = 1784 Hz, ^2^*J*(^119^Sn–^1^H) = 87 Hz] which collapsed to a singlet upon ^1^H decoupling. Collectively these data correspond to the previously unreported dihydride [(Me_3_Si)_2_CH]_2_SnH_2_ (**2**), which was confirmed by comparison with an authentic sample prepared by the reaction of LiAlH_4_ and [(Me_3_Si)_2_CH]_2_SnCl_2_ (see ESI† for details).

### Isotopic investigation

When D_2_ was used in place of H_2_, the methine peak present in the ^1^H NMR spectrum of the product mixture resolved as a singlet, while the Sn–H signal was absent and replaced by a Sn–D signal at *δ* = 5.11 ppm [^1^*J*(^117^Sn–^2^H) = 262 Hz, ^1^*J*(^119^Sn–^2^H) = 274 Hz] in the ^2^H NMR spectrum. These results demonstrate the formation of dideuteride **2-D_2_**,^20^ and that the Sn-bound protons in **2** must originate from the hydrogen atmosphere.

In order to probe the mechanism further, a *d*_8_-toluene solution of **1**/[**1**]_2_ and Et_3_N was reacted with a 1 : 1 mixture of H_2_/D_2_. The resultant ^1^H NMR spectrum was very similar in appearance to that of **2**, with two exceptions: the relative integration of the Sn–H peak did not match that of the methine signal (1.2 : 2; consistent with the faster rate of reaction with H_2_*vs.* D_2_ – *vide infra*), and the C–*H* resonance was composed of overlapping peaks commensurate with a mixture of **2** and **2-D_2_**. No spectroscopic evidence was seen for the formation of **2-HD**, which was independently and selectively obtained by analogous reaction of **1**/[**1**]_2_ under an HD atmosphere. These observations provide strong evidence that delivery of both atoms from H_2_/D_2_/HD to a single Sn centre occurs either simultaneously, or in a near-concerted fashion.

### Kinetic analysis

**Scheme 1:**
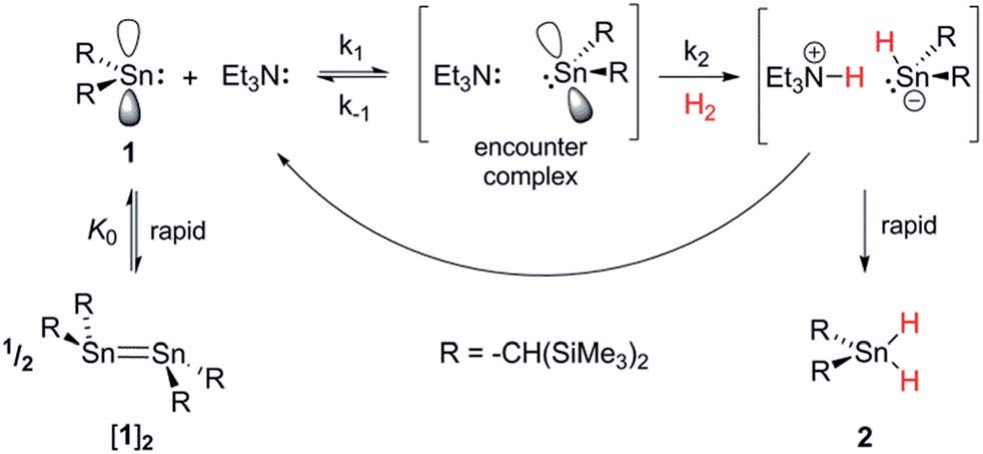
Proposed reaction mechanism for H_2_ heterolysis by **1**, catalysed by Et_3_N.

By analogy with established FLP systems, and the microscopic reverse of the polar mechanism by which dehydrogenation of ArSnH_3_ species is proposed to occur,15*a* we envisaged a reaction mechanism in which **1** and Et_3_N form a weakly associated ‘encounter complex’ which subsequently reacts with H_2_ (Scheme 1).^21^ Assuming that encounter complex formation is a rapid pre-equilibrium prior to rate-limiting H_2_ activation gives the expected rate law: rate = *k*′[**1**][Et_3_N][H_2_], where *k*′ = (*k*_1_*k*_2_)/*k*_–1_. Calorimetric studies on H_2_ activation by the FLP Mes_3_P/B(C_6_F_5_)_3_ (Mes = 2,4,6-C_6_Me_3_H_2_) found the rate to be very accurately modelled as a single, termolecular step, which formally gives the same rate law.^22^

**Fig. 3:**
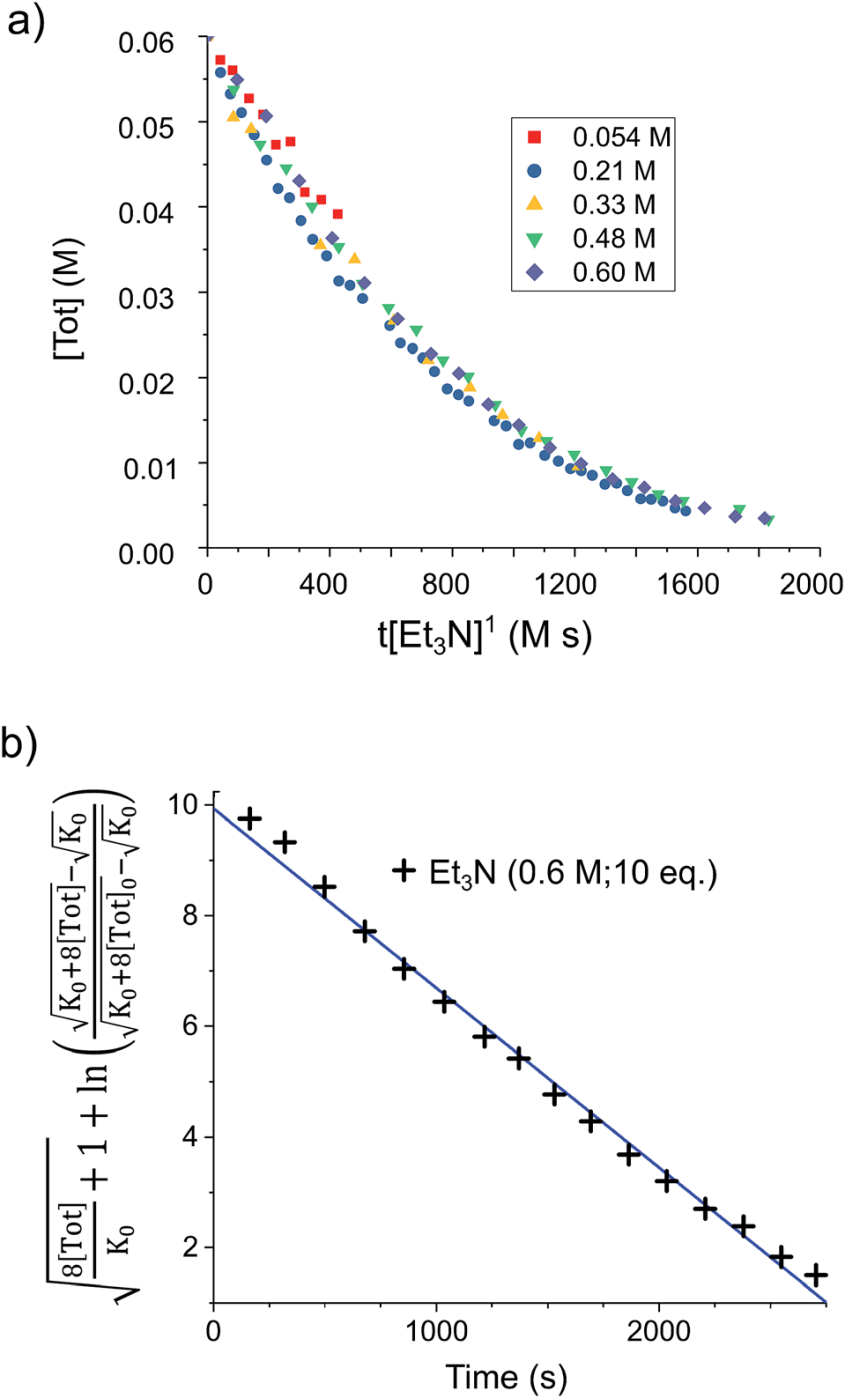
(a) Solutions of **1** (0.03 mmol) in *d*_8_-toluene (0.5 mL) under H_2_ (4 bar) containing various base concentrations were prepared. When the stannylene concentration, [Tot], is plotted against the normalised timescale *t*·[Et_3_N]^1^, all traces overlap, confirming the order in base to be one. (b) Linearised rate data for a similar solution of **1** (0.03 mmol) in *d*_8_-toluene (0.5 mL) under H_2_ (4 bar) containing Et_3_N (0.6 M; 10 eq.).

To confirm the order of catalytic Et_3_N, the method of time (*t*) scale normalisation was used;^23^ normalisation to the scale of *t*·[Et_3_N]^*x*^ resulted in the superposition of all reactant traces only when *x* = 1, confirming the rate to be first order with respect to the amine (Fig. 3a). Determination of reaction order with respect to **1** requires its concentration to be known accurately at any given time in a reaction mixture. However, since the observed ^1^H NMR resonances are a weighted average of the signals from **1** and [**1**]_2_ (Δ*G*_293K_ = 3.1 kcal mol^–1^), with both species present at significant concentrations under reaction conditions, simple observation of the concentration of **1** is not directly possible by ^1^H NMR spectroscopy.18*a* The concentration of **1** can, however, be calculated from the total concentration of “R_2_Sn” species in solution, [Tot], present as either monomer or dimer, which are related to the concentrations of **1** and [**1**]_2_ by:

1 [Tot] = [**1**] + 2[(**1**)_2_]

The dimerisation equilibrium of **1** can be expressed as:
2

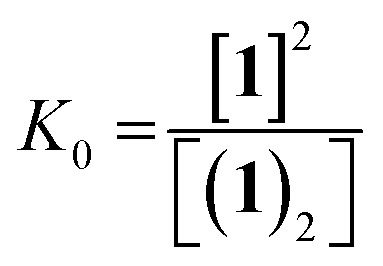




Combining eqn (1) and (2) and solving for [**1**] yields:
3

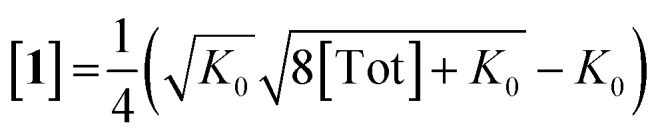




Inserting eqn (3) into the expected rate law (*vide supra*) gives:
4

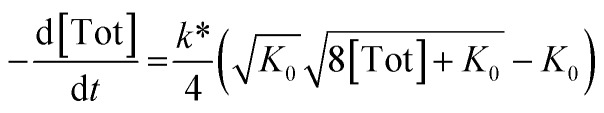

where, if the amount of H_2_ is sufficiently high that its concentration remains approximately constant:
5

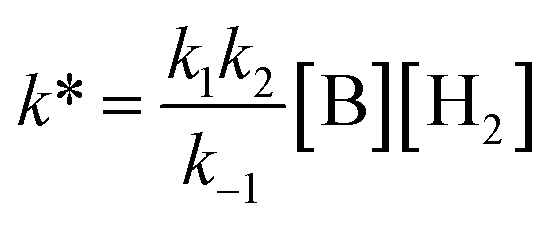




Rearrangement and integration by substitution of eqn (4) (see ESI†) gives:
6

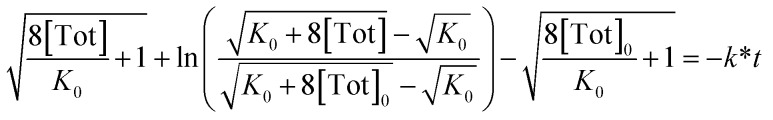




Therefore, plotting the variable portion of the LHS of this expression against *t* gives a straight line of gradient –*k*∗, confirming the proposed first-order dependence on **1** (Fig. 3b).

Using the known value of [H_2_] in toluene at 4 bar (293 K)^24^ provides a value of 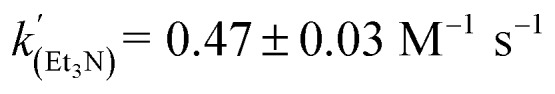.^25^ As well as Et_3_N, 2-*tert*-butyl-1,1,3,3-tetramethylguanidine (Barton's base, TBTMG) and 1,2,2,6,6-pentamethylpiperidine (PMP), were also found to form FLPs with **1**/[**1**]_2_, with corresponding rates of H_2_ cleavage: 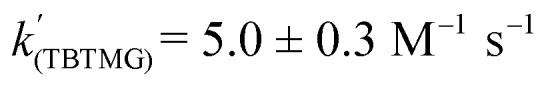, 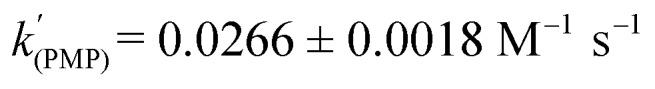.^26^ Despite the similar basicity to Et_3_N, the bulkier Hünig's base (iPr_2_EtN; p*K*_a(MeCN)_: 18.0)^27^ was ineffective for H_2_ heterolysis, as was the weaker base 2,4,6-collidine (p*K*_a(MeCN)_: 14.98).^28^ Clearly H_2_ activation requires that the LB be sufficiently basic and not too sterically encumbered, in line with observations of other FLP systems.^29^

A kinetic analysis of the isotopic systems permitted quantification of the KIE: 
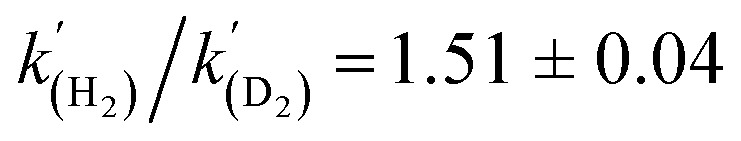
 when Et_3_N was used as the base. In addition, the acceleration in rate from a more polar solvent could also be quantified: 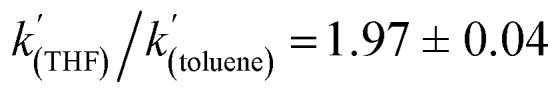 (when Et_3_N was used).

### Coordinating bases

**Scheme 2:**
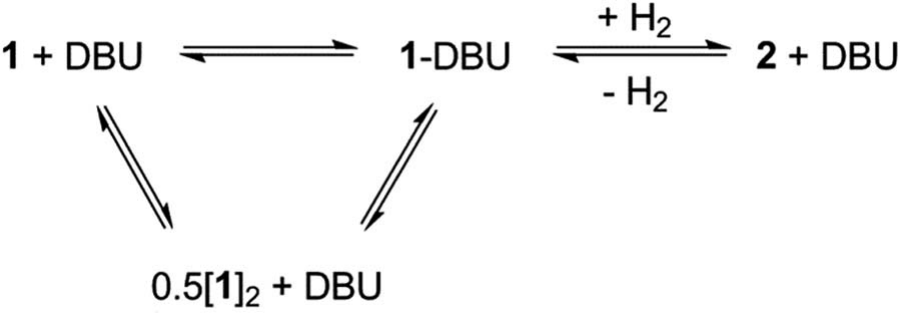
Equilibrium between product **2** + DBU and the dehydrogenated mixture **1**·DBU ↔ **1** ↔ [**1**]_2_.

When the less sterically bulky 1,8-diazabicyclo[5.4.0]undec-7-ene (DBU) is used, an interaction with **1** can be clearly seen in the ^13^C{^1^H} NMR spectrum: upon gradual addition of DBU to **1**/[**1**]_2_, the methine resonance undergoes a substantial upfield shift, reaching a limiting value of *δ* = 18.5 ppm (10-fold excess of DBU). Using the established ^13^C NMR chemical shift values for **1** and [**1**]_2_ (60.0 ppm and 28.7 ppm, respectively),18*a* this is consistent with a fast equilibrium between **1**·DBU, **1** and [**1**]_2_ (Scheme 2; see ESI† for full details). A value of Δ*G* = –3.7 ± 0.2 kcal mol^–1^ for the formation of **1**·DBU from [**1**]_2_ was obtained from a van't Hoff analysis of variable temperature UV-Vis spectra.

While the reaction of **1**/DBU mixtures (containing 0.1–10 equivalents of DBU) with H_2_ proceed rapidly, they do not reach completion, indicative of a reversible process (see Fig. S7 in ESI†).

**Fig. 4:**
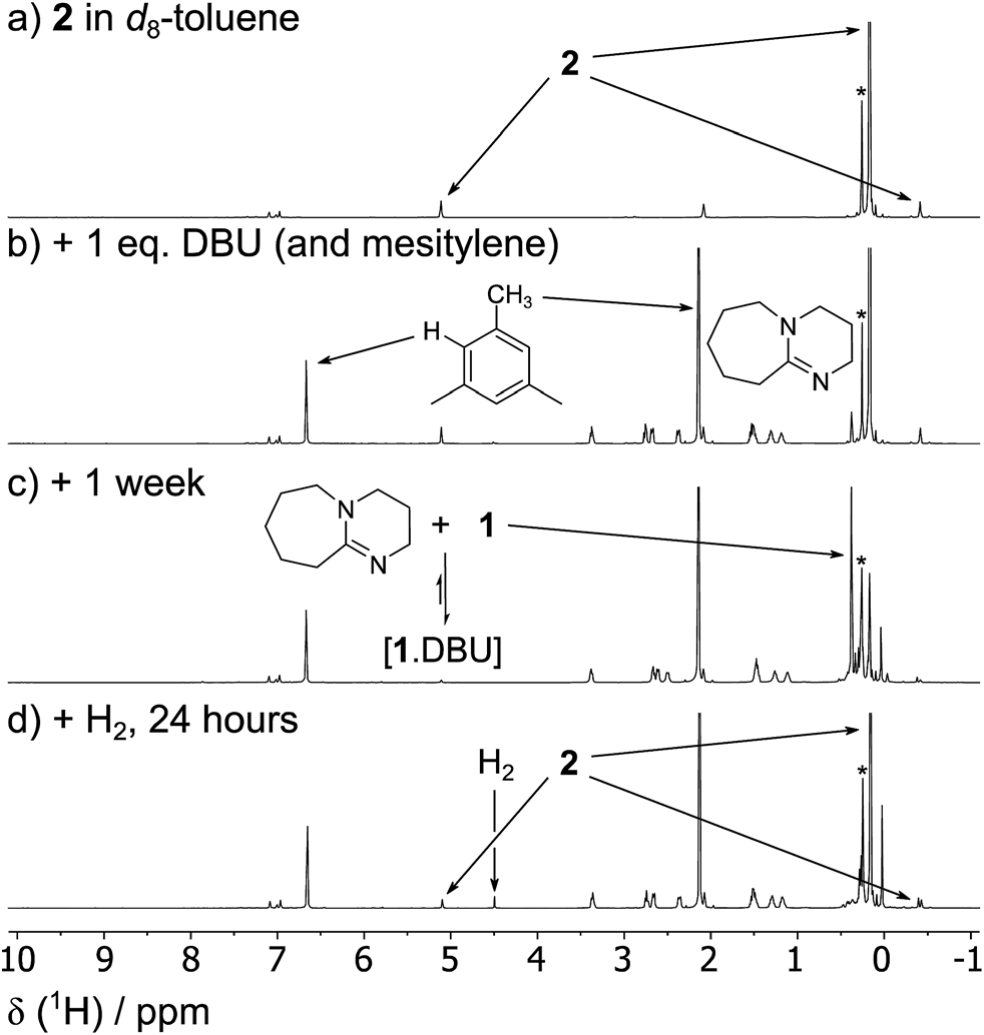
The reversibility of the reaction between **1**/[**1**]_2_, DBU and H_2_ can be shown explicitly by a series of ^1^H NMR spectra depicting: (a) a solution of **2** (0.03 mmol) in *d*_8_-toluene (0.5 mL) (b) the same solution with added DBU (0.03 mmol, 1 equivalent) and mesitylene (2%) as an internal standard; (c) after degassing three times over the course of one week, showing the formation of **1**·DBU ↔ **1** ↔ [**1**]_2_; (d) reformation of **2** after the addition of H_2_ (4 bar). *Small amount of silicone grease from the independent synthesis of **2**.

The reversibility can be explicitly demonstrated by the (C*H*_3_)_3_Si region of the ^1^H NMR spectrum, whereby addition of DBU to a solution of **2** led to the appearance of a signal corresponding to the dehydrogenated mixture **1**·DBU ↔ **1** ↔ [**1**]_2_; this increased in intensity at the expense of the (C*H*_3_)_3_Si peak of **2** (Fig. 4a–c). No H_2_ is observed in the ^1^H NMR spectrum as the solution was degassed multiple times in order to accelerate the reaction – however, the very small amount of H_2_ generated (approx. 0.3 bar) would likely hamper detection. Furthermore, the methine resonance of the **1**·DBU ↔ **1** ↔ [**1**]_2_ mixture is subject to a significant upfield shift compared to [**1**]/[**1**]_2_ (dependent upon the DBU concentration), and so is obscured beneath the relatively intense (C*H*_3_)_3_Si region. Upon charging this reaction with H_2_, restoration of **2** was rapidly observed (Fig. 4d). For the equilibrium involving H_2_ (Scheme 2), an equilibrium constant, *K*_eq_ = 164 ± 5, in favour of **2** can be calculated from the relative intensities of the (C*H*_3_)_3_Si resonances, providing Δ*G* = –3.0 kcal mol^–1^ (1 bar H_2_).

Using the similarly unhindered but less basic 4-(dimethyl amino)pyridine (DMAP) also gave an adduct **1**·DMAP, but no reaction with H_2_ at room temperature. However, heating a solution of **1** with excess DMAP (4 bar H_2_, 2 h, 100 °C) yielded **2** in 31% conversion.

### Computational investigation

**Fig. 5:**
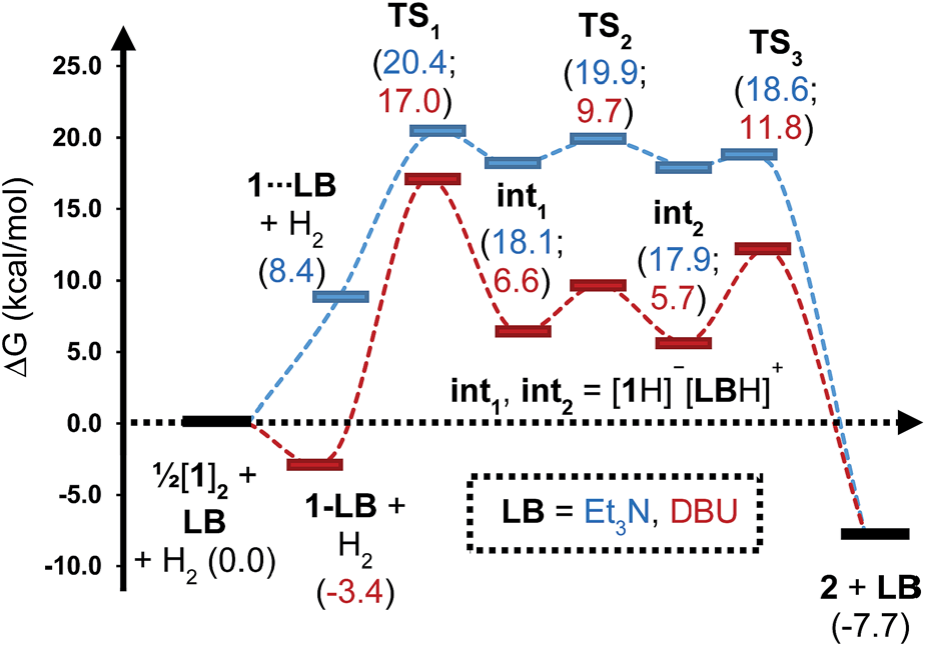
Computed free energy profile for Et_3_N- and DBU-assisted H_2_ activation with **1**. Relative free energies (in kcal mol^–1^) are with respect to 0.5·[**1**]_2_ + LB + H_2_.

**Fig. 6:**
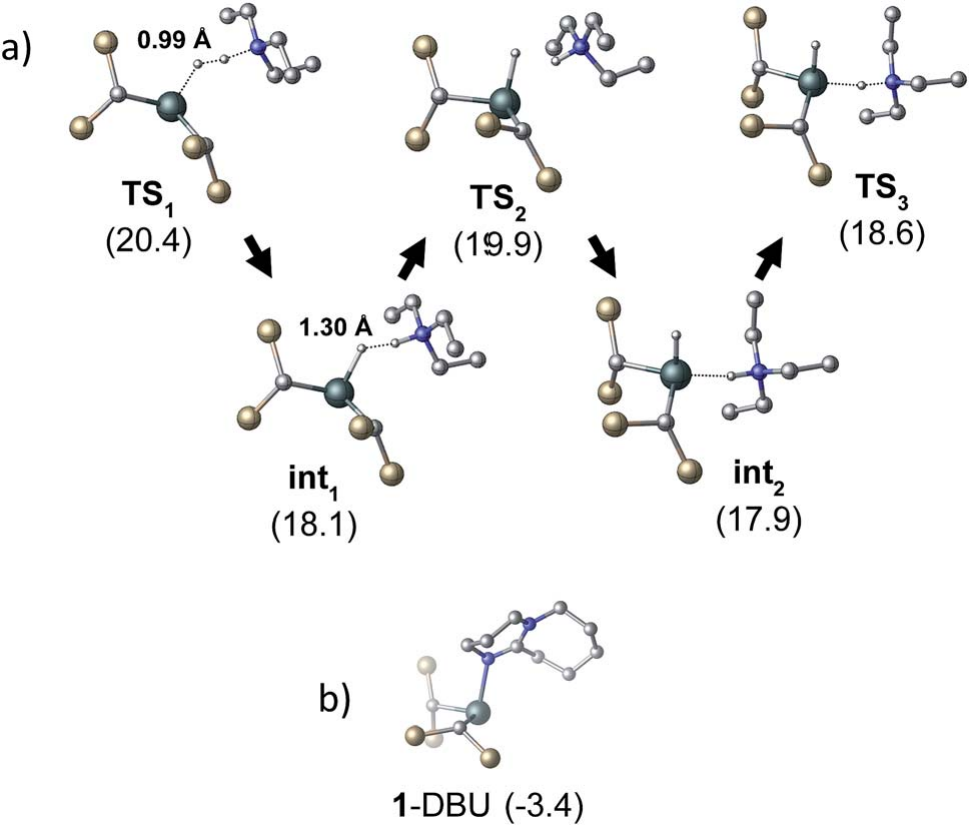
(a) Structural representations of the computed transition states for the heterolysis of H_2_ by **1** and Et_3_N. H–H distances are given for **TS_1_** and **int_1_**. (b) The computed adduct formed between **1** and DBU. All energies (in kcal mol^–1^) are relative to 0.5·[**1**]_2_ + LB + H_2_. Si–*CH*_3_ and C–*H* groups omitted for clarity.

To gain further insight into the mechanism of H_2_ activation, DFT calculations were performed for various **1**/LB pairs;^31^ the computed reaction profiles for both the Et_3_N- and DBU-mediated reactions are depicted in Fig. 5. When LB = Et_3_N, the reaction was found to proceed *via* initial H_2_ heterolysis leading to a tight ion pair intermediate [**1**H]^–^[Et_3_NH]^+^ (**int_1_**). Facile rearrangement to **int_2_** and subsequent delivery of the H^+^ to the lone pair on the [**1**H]^–^ moiety furnishes **2** (Fig. 6a); a very similar mechanism was found when LB = DBU. In support of this polar mechanism, the rate using Et_3_N as the LB was found to be faster in THF 
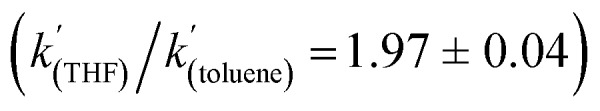
. The low barriers to rearrangement of the intermediates also offer an explanation as to why H/D exchange is not observed upon reaction with an H_2_/D_2_ mixture or HD: collapse of the ion pairs is likely much faster than solvent cage escape.

Although the located transition states (TSs) are energetically close-lying, the overall reaction barrier appears to be determined by the H_2_ splitting step, which is in line with kinetic measurements. Free energy data computed for the H_2_ splitting step for reactions with different bases are compiled in Table 1 alongside other properties. For Et_3_N, TBTMG and PMP, no favourable adduct formation was found with **1**, and the Δ*G*^‡^ values follow the order TBTMG < Et_3_N < PMP, which is consistent with experimental reaction rates. For the coordinating bases DBU (Fig. 6b) and DMAP, adducts favourable relative to free [**1**]_2_ and base were computationally determined. This reduces the absolute value of Δ*G*_reaction_ such that an equilibrium is experimentally observed in the case of DBU. For DMAP, the activation barrier is found to be much higher, paralleling results seen by experiment where elevated temperatures are required to obtain product **2**.

**Table 1:** Computational and p*K*_a_ data for reactions of a series of bases with **1** and H_2_^*a*^

Property	Et_3_N	TBTMG	PMP	DBU	DMAP
**1**·LB^*a*^	—	—	—	–3.4	–3.6
TS_1_^*a*^	20.4	18.3	21.4	17.0	20.1
**int_1_** ^*a*^	18.1	5.4	16.1	6.6	16.8
Δ*G*^‡^^*a*^	20.4	18.3	21.4	20.4	23.7
Δ*G*_reaction_^*a*^	–7.7	–7.7	–7.7	–4.3	–4.1
PA^*a*^ ^,^^*b*^	–270.1	–286.0	–272.7	–283.5	–272.2
p*K*_a_^*c*^	18.8	23.6	18.7	24.3	18.0
*d*(HH)^*d*^/Å	0.99	0.87	0.96	0.88	0.99

^*a*^Free energy data relative to 0.5·[**1**]_2_ + base + H_2_ (kcal mol^–1^); Δ*G*^‡^ is activation free energy.

^*b*^Proton affinity is defined as the free energy of base + H^+^ → baseH^+^.

^*c*^Measured in MeCN.^30^

^*d*^H–H distance in TS_1_ (0.76 Å in free H_2_).

The energies of all intermediates **int_1_** are computed to be well above the reference state, which follows from the weak Lewis acidity of **1**. The stabilities of **int_1_** species correlate very well with the general trend in PA and p*K*_a_, but this is not strictly true for the TSs, where steric factors are more important. Unstable **int_1_** intermediates imply late TSs for the H_2_ activation step, which is shown by significantly elongated H–H distances in the TS structures. The experimentally observed KIE (1.51 ± 0.04) supports this finding, which is commensurate with rate-limiting H_2_/D_2_ activation involving considerable H–H/D–D bond breaking.^32^

## Conclusions

In conclusion, we have demonstrated the ability of FLP-mediated reactivity to enable the formal oxidative addition of H_2_ to an otherwise inert MG centre, and in doing so have also observed the first example of reversible H_2_ addition to a single-site MG complex. We have utilised experimental and computational means to comprehensively explore the mechanism of this transformation and found that H_2_ activation in this system differs from those based on more typical FLPs, due to the high-energy nature of the immediate H_2_ splitting products, resulting in rare examples of late TSs. The development of methods to harness this FLP-promoted OA/RE H_2_ reactivity for hydrogenation catalysis is currently underway.

## Conflicts of interest

There are no conflicts to declare.

## Supplementary Material

Supplementary information
