# Supplementary material for: Base-induced reversible H2 addition to a single Sn(ii) centre
Source: Chem Sci. 2018 Sep 18;9(46):8716–22. doi: 10.1039/c8sc03110j (PMC6289099; doi:10.1039/c8sc03110j)
Supplement: Supplementary file 1 [file SC-009-C8SC03110J-s001.pdf]

Electronic Supplementary Material (ESI) for Chemical Science.
This journal is © The Royal Society of Chemistry 2018

Base-Induced Reversible H_2 Activation at a Single Sn(II) Centre

Electronic Supplementary Information

Roland C. Turnell-Ritson,[a] Joshua S. Sapsford,[a] Robert T. Cooper,[a] Stella S. Lee,[a] Tamás Földes,[b] Patricia A. Hunt,[a] Imre Pápai*,[b] and Andrew E. Ashley*,[a]

[a] Department of Chemistry, Imperial College London, London, UK, SW7 2AZ

[b] Research Center for Natural Sciences, Hungarian Academy of Sciences, P.O. Box 17, H-1525 Budapest, Hungary

Contents **Page**

1. General experimental considerations S3
2. Independent synthesis of R_2SnH_2 (**2**) S3
3. NMR Spectra S4
   3.1. General Procedure of H_2 activation using **1** / base S4
   3.2. H_2 activation with Et_3N S5
      3.2.1. D_2 activation with Et_3N S6
      3.2.2. Reaction with H_2/D_2 (1:1) and HD S7
   3.3. H_2 activation with TBTMG S8
   3.4. H_2 activation with PMP S9
   3.5. H_2 activation with DBU S10
      3.5.1. Explicit reversibility S11
   3.6. H_2 activation with DMAP S12
   3.7. Determination of equilibrium constant for H_2 activation with DBU, $\mathbf{K_{eq}}$ S13
      3.7.1. NMR spectra of **1** / DBU equilibrium S14
      3.7.2. Variable temperature UV-Visible spectroscopy of **1** / DBU equilibrium S15
4. Kinetic Analysis S17
   4.1. Order in base S18
   4.2. Mathematical Treatment S19
      4.2.1. Determination of Et_3N rate constant, $\boldsymbol{k'_{(Et3N)}}$ S21
         4.2.1.1. Determination of kinetic isotope effect, KIE S22
         4.2.1.2. Comparison of toluene and THF rate constants S22
      4.2.2. Determination of TBTMG rate constant, $\boldsymbol{k'_{(TBTMG)}}$ S23
      4.2.3. Determination of PMP rate constant, $\boldsymbol{k'_{(PMP)}}$ S24
5. Computational details S25
   5.1. Computational results S26
      5.1.1. Dimerisation of **1** S26

Page

5.1.2. H_2 addition in the absence of base S27

5.1.3. H_2 addition with the assistance of Et_3N S27

5.1.4. H_2 addition with the assistance of DBU S29

5.1.5. H_2 addition with other bases S31

5.2. Total energy data S32

5.3. Cartesian coordinates of the geometry optimised structures S34

6. References S65

1. General experimental considerations

All reactions were performed under N_2 atmosphere unless stated otherwise. All manipulations were carried out either in an MBraun Labmaster DP glovebox or by using standard Schlenk line techniques. All glassware was dried by heating to 170 °C overnight before use. All solvents were degassed and dried before use: Et_2O was distilled from dark green Na/fluorenone indicator and stored over a K mirror; pentane was dried using an Innovative Technology Pure Solv™ SPS-400 and stored over a K mirror; d_8-toluene was freeze-pump-thaw degassed, dried over 4 Å molecular sieves and stored over a K mirror; d_8-THF was freeze-pump-thaw degassed and dried over 4 Å molecular sieves. All other compounds were purchased from major suppliers: Et_3N, 2-*tert*-butyl-1,1,3,3-tetramethylguanidine (Barton's base, TBTMG), 1,2,2,6,6-pentamethylpiperidine (PMP) and 1,8-diazabicyclo[5.4.0]undec-7-ene (DBU) were freeze-pump-thaw degassed, distilled and dried over 4Å molecular sieves, and 4-dimethylaminopyridine (DMAP) was recrystallised from toluene and dried *in vacuo*. H_2 was purchased from BOC (research grade) and dried by passage through a Matheson Tri-Gas Weldassure™ Purifier drying column. D_2 (99.8% D) was purchased from Cambridge Isotope Laboratories and dried over 3 Å molecular sieves (1 bar), or purchased from Aldrich (99.8 % D) and dried by passing through a Supelco Supelpure®-O oxygen/moisture trap (4 bar). HD was purchased from Aldrich (96 % HD, 98 % D) and dried by standing over 4 Å molecular sieves. Elemental analysis was performed by Stephen Boyer of London Metropolitan University. NMR spectra were recorded on Bruker AV-400, Bruker AV-500 and DRX-400 spectrometers. ^1H, ^2H and $^{13}C\{^1H\}$ spectra were referenced internally to residual solvent signals, while $^{29}Si\{^1H\}$ and $^{119}Sn\{^1H\}$ spectra were referenced externally to $SiMe_4$ and $SnMe_4$ respectively. Chemical shifts are stated in ppm (s = singlet, d = doublet, t = triplet).

Bis[bis(trimethylsilyl)methyl]tin(II) (**1**)[1] and *bis*[bis(trimethylsilyl)methyl]dichlorotin(IV)[2] were prepared according to literature procedures.

2. Independent synthesis of R_2SnH_2 (2) (R = $CH(SiMe_3)_2$)

A solution of *bis*[bis(trimethylsilyl)methyl]dichlorotin (490 mg, 0.964 mmol) in Et_2O (10 mL) was added dropwise to a stirred suspension of $LiAlH_4$ (80.5 mg, 2.12 mmol) in Et_2O (10 mL) at room temperature. The mixture was stirred for three hours, filtered *via* cannula and the solvent removed under reduced pressure. The oily residue was extracted into *n*-pentane, filtered, and the solvent removed *in vacuo*, yielding the product as a viscous, colourless oil (315 mg, 0.716 mmol, 75 %).

^1H NMR (400 MHz, C_6D_6) δ: -0.39 [2H, t, $^3J(^1H-^1H)$ = 2.2 Hz, $^2J(^{117}Sn-^1H)$ = 83 Hz, $^2J(^{119}Sn-^1H)$ = 87 Hz, C*H*], 0.19 [36H, s, CH_3], 5.15 [2H, t, $^3J(^1H-^1H)$ = 2.2 Hz, $^1J(^{115}Sn-^1H)$ = 1566 Hz , $^1J(^{117}Sn-^1H)$ = 1704 Hz, $^1J(^{119}Sn-^1H)$ = 1784 Hz, SnH_2]. $^{13}C\{^1H\}$ NMR (101 MHz, C_6D_6) δ: -0.7 [s, $^1J(^{29}Si-^{13}C)$ = 40 Hz, $^1J(^{117}Sn-^{13}C)$ = 171 Hz, $^1J(^{119}Sn-^{13}C)$ = 179 Hz, *C*H], 2.7 [s, $^1J(^{29}Si-^{13}C)$ = 51 Hz, $^1J(^{117/119}Sn-^{13}C)$ = 19 Hz, *C*H_3]. $^{29}Si\{^1H\}$ NMR (79.5 MHz, C_6D_6) δ: 2.2 (s). $^{119}Sn\{^1H\}$ NMR (149 MHz, C_6D_6) δ: -195 (s). HRMS (EI): m/z found (calculated) for $C_{14}H_{39}Si_4Sn^+$: 439.1155 (439.1145). Anal. calcd. for $C_{14}H_{40}Si_4Sn$: C, 38.26; H, 9.17. Found: C, 38.08; H, 8.95.

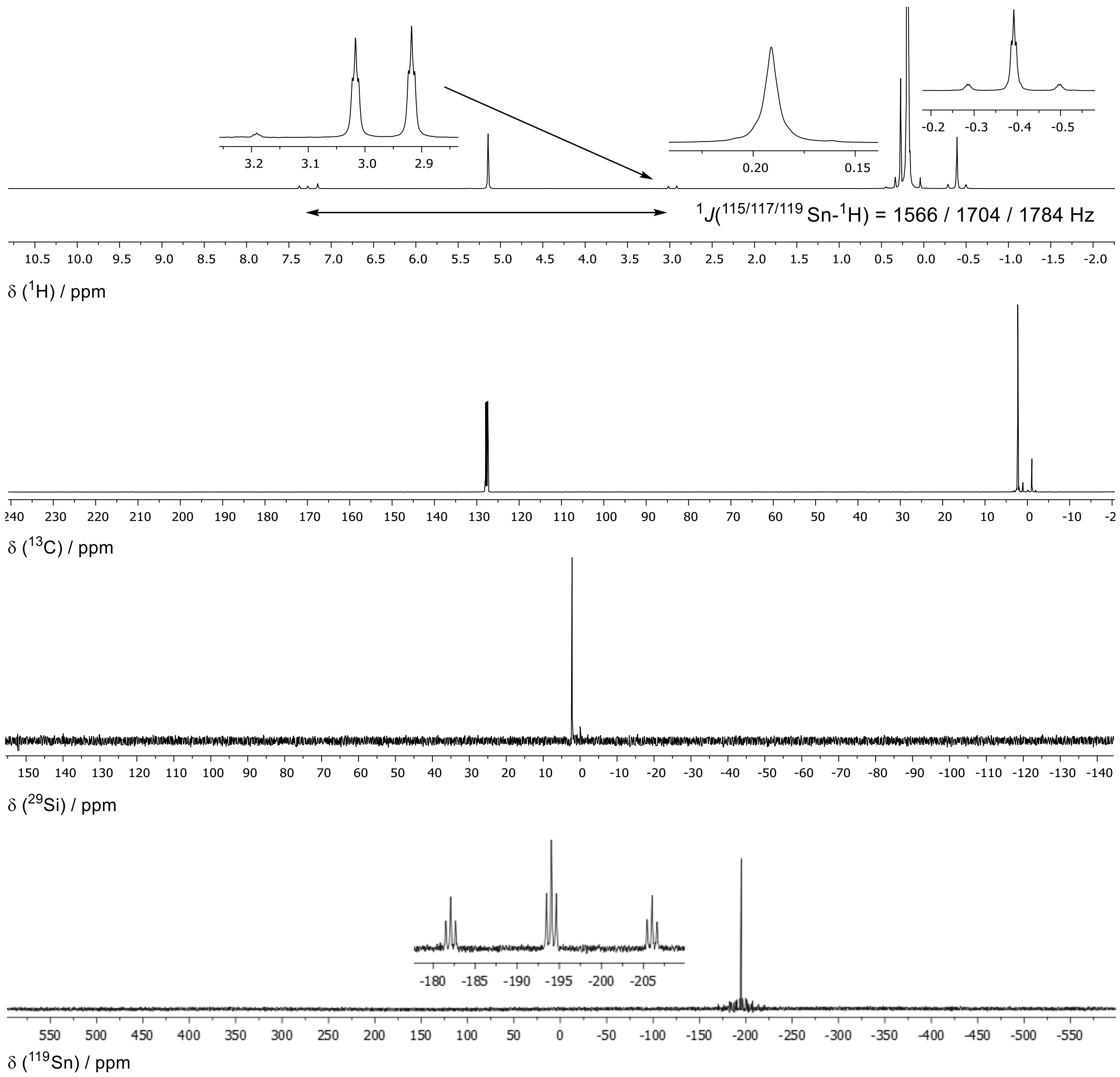

Figure S1 – ^{1}H [with inset showing $^{1}J(^{115/117/119}$Sn-^{1}H) satellites], ^{13}C{^{1}H}, ^{29}Si{^{1}H} and ^{119}Sn{^{1}H} (with ^{119}Sn inset) NMR spectra of **2** (approx. 0.06 M in C_6D_6).

3. NMR spectra

3.1. General procedure for H_2 activation using 1 / base

1 (13.1 mg, 0.03 mmol) was dissolved in d_8-toluene (0.5 mL) in an NMR tube fitted with a J. Young's valve, with mesitylene (10 μL) as an internal standard. An initial ^{1}H NMR spectrum was recorded. A specified amount of base was added, another ^{1}H NMR spectrum was recorded, and subsequently H_2 was added *via* a freeze-pump-thaw method (1 bar at -196 ºC, *ca*. 4 bar at RT). The sample was allowed to stand for several days (with regular agitation), its progress being recorded by ^{1}H NMR spectroscopy.

3.2. H_2 activation with Et_3N

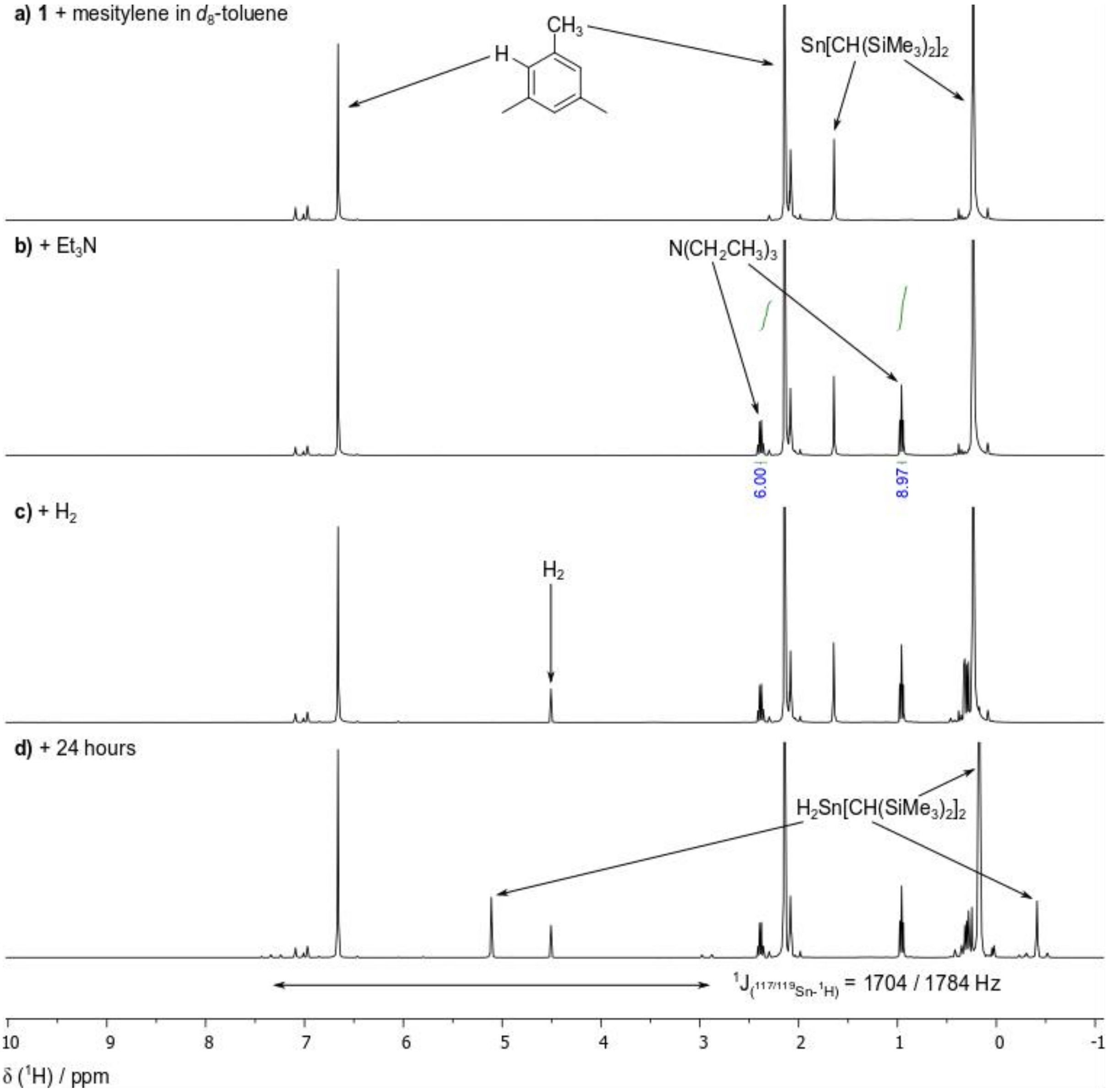

Figure S2 – Series of ^{1}H NMR spectra depicting **a)** a solution of **1** in d_8-toluene (0.06 M) with mesitylene (2%); **b)** Et_3N (0.2 equivalents) added to the solution; **c)** H_2 (4 bar) admitted to the same solution; **d)** subsequent reaction.

3.2.1. D_2 activation with Et_3N

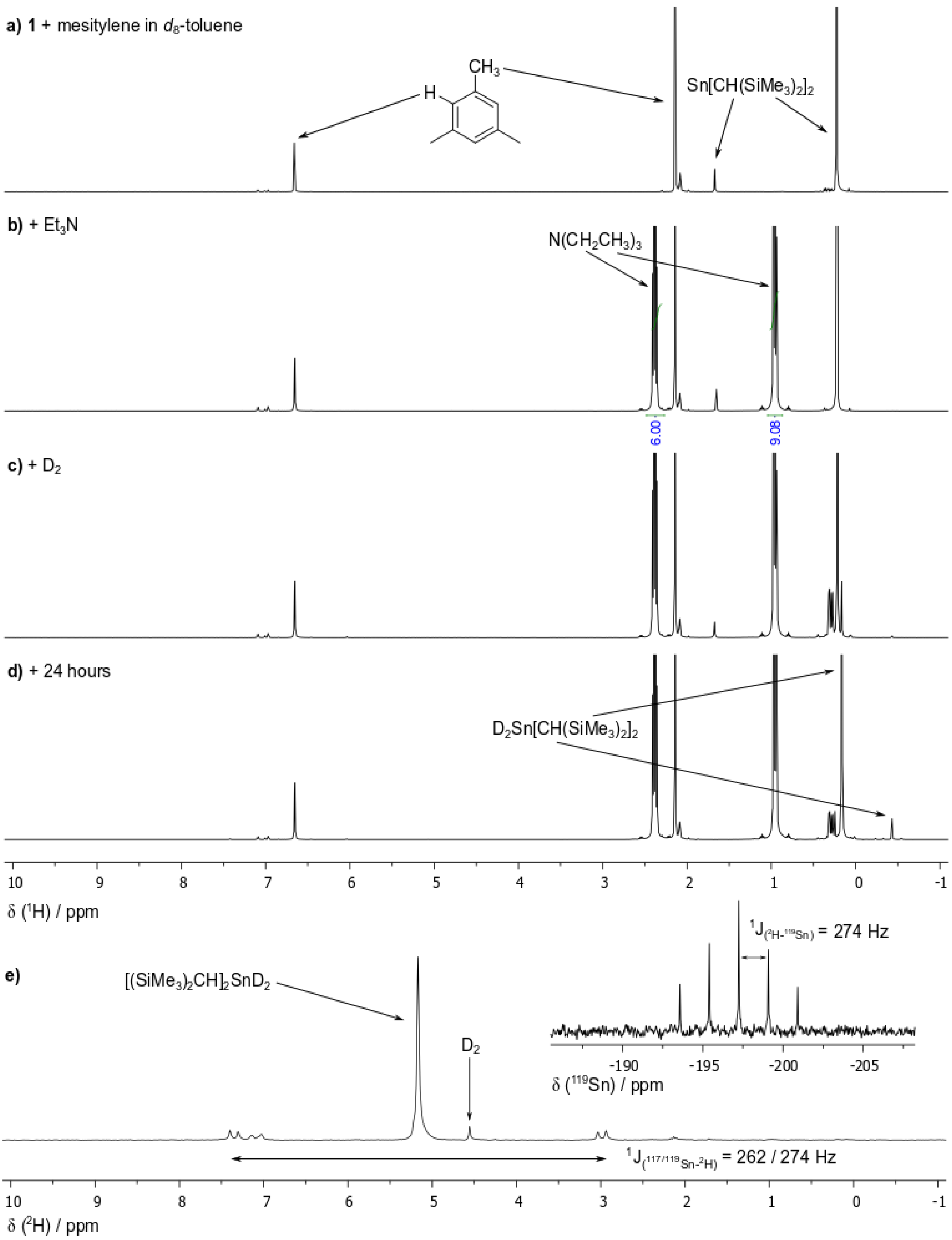

Figure S3 – Series of ^1H NMR spectra depicting **a)** a solution of **1** in d_8-toluene (0.06 M) with mesitylene (2%); **b)** Et_3N (10 equivalents) added to the solution; **c)** D_2 (4 bar) admitted to the same solution; **d)** subsequent reaction. **e)** ^2H NMR spectrum of the end point of the same reaction in *proteo*-toluene, with inset showing $^{119}Sn\{^1H\}$ NMR spectrum. 1:2:3:2:1 quintet caused by coupling to two equivalent I = 1 atoms.

3.2.2. Reaction with H_2/D_2 (1:1) and HD

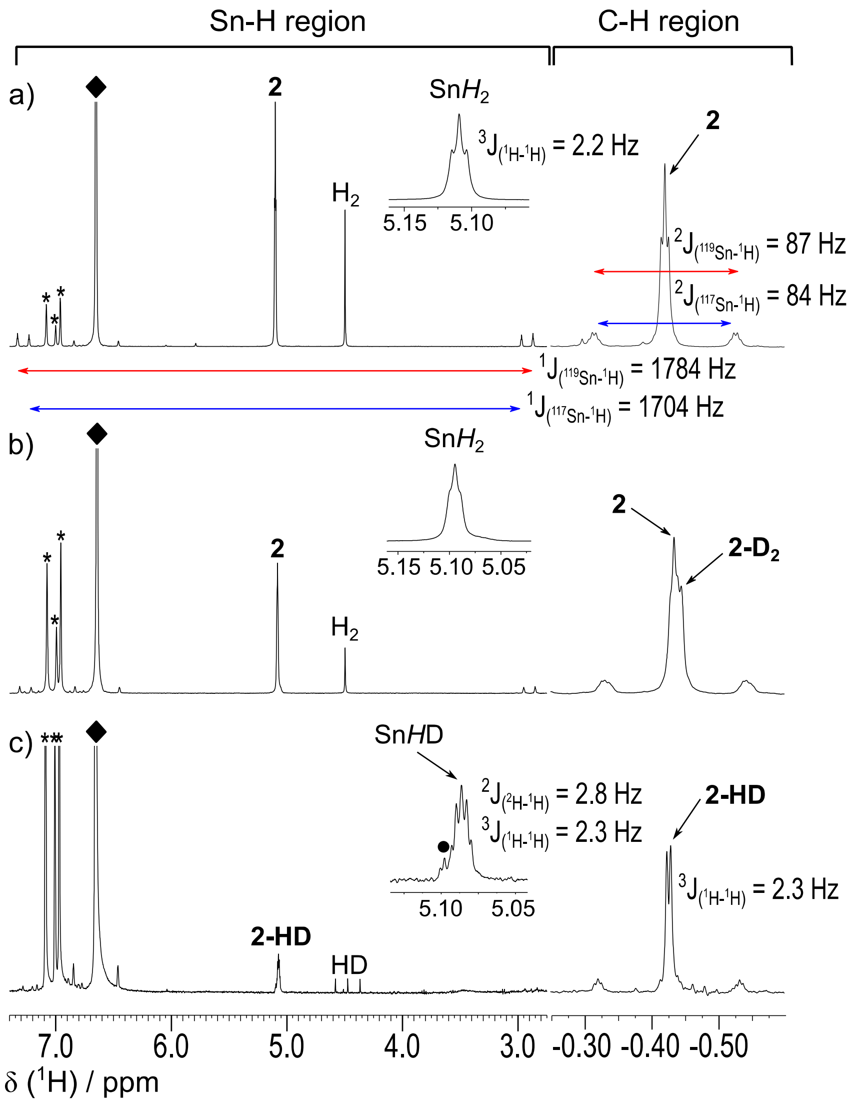

Figure S4– Series of ^1H NMR spectra depicting the Sn-H (insets showing expansions of the Sn-H peak) and C-H methine region of the products of reaction between **a) 1** and Et_3N (10 equivalents) under H_2 (4 bar); **b) 1** and Et_3N (10 equivalents) under H_2:D_2 1:1 (1 bar); **c) 1** and Et_3N (10 equivalents) under HD (1 bar). The very similar values of the $^2J_{(2H-1H)}$ and the $^3J_{(1H-1H)}$ coupling constants (2.8 Hz and 2.3 Hz, respectively) result in an unusual 1:3:4:3:1 *pseudo*-quintet. * - residual solvent peaks, ♦ - mesitylene (internal standard), ● – Sn-H_2 signal, formed from reaction with residual H_2 in commercially obtained HD feed gas.

3.3. H_2 activation with TBTMG

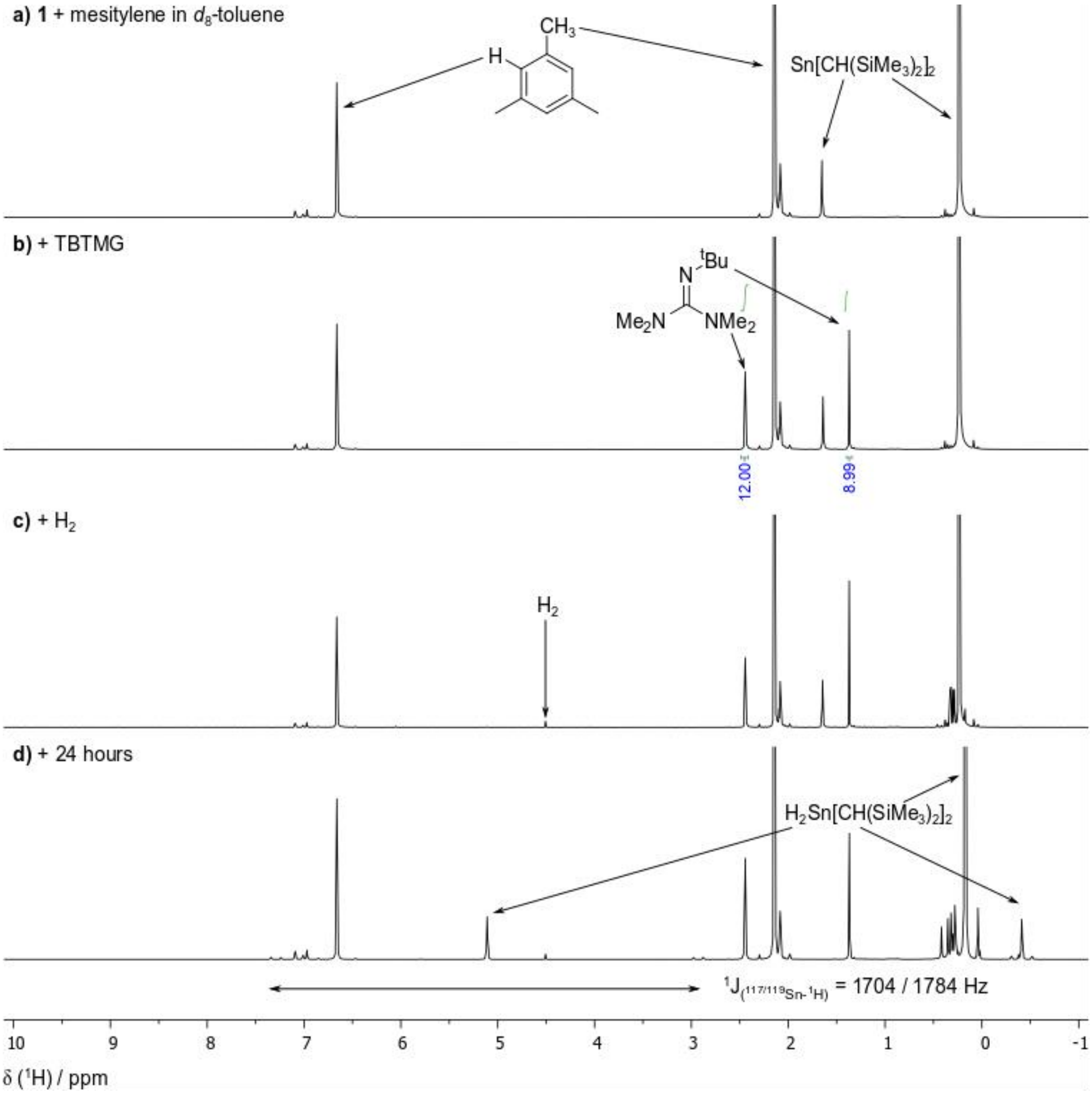

Figure S5 – Series of ^{1}H NMR spectra depicting **a)** a solution of **1** in d_8-toluene (0.06 M) with mesitylene (2%); **b)** TBTMG (0.2 equivalents) added to the solution; **c)** H_2 (4 bar) admitted to the same solution; **d)** subsequent reaction.

3.4. H_2 activation with PMP

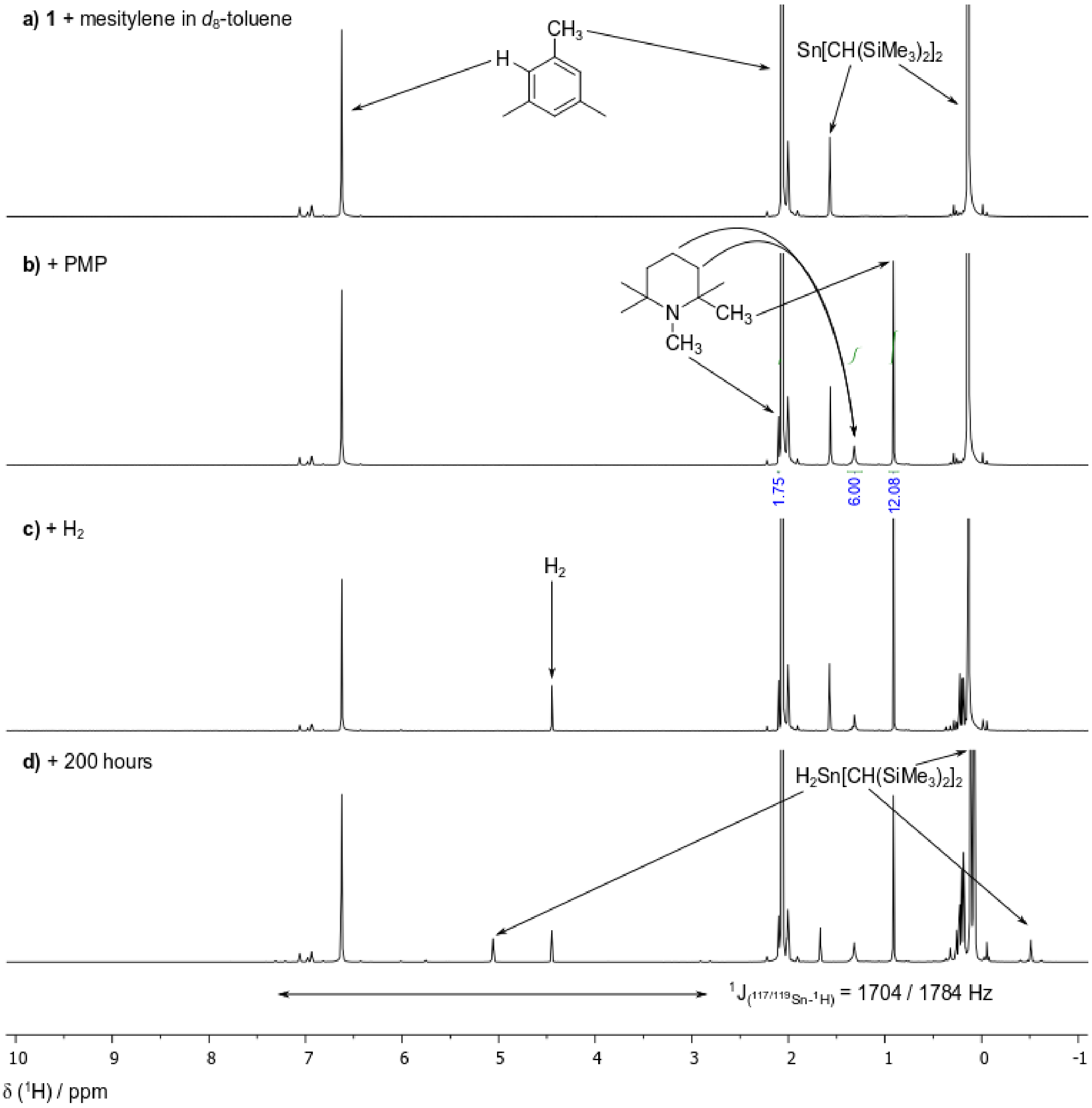

Figure S6 – Series of ^{1}H NMR spectra depicting **a)** a solution of **1** in d_8-toluene (0.06 M) with mesitylene (2%); **b)** PMP (0.2 equivalents) added to the solution; **c)** H_2 (4 bar) admitted to the same solution; **d)** subsequent (incomplete) reaction.

3.5. H_2 activation with DBU

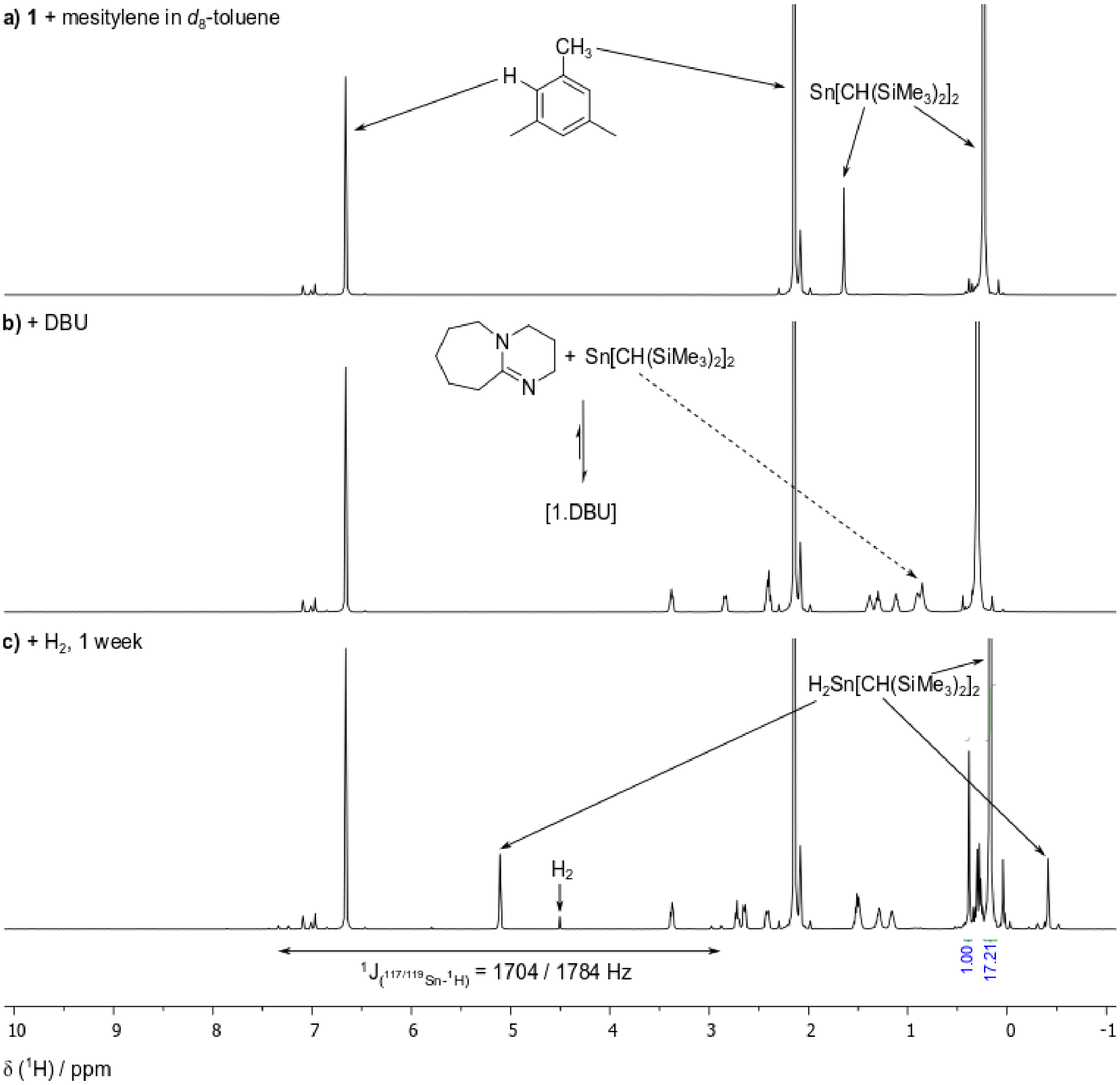

Figure S7 – Series of ^{1}H NMR spectra depicting **a)** a solution of **1** in d_8-toluene (0.06 M) with mesitylene (2%); **b)** DBU (0.5 equivalents) added to the solution; **c)** H_2 (1 bar) admitted to the same solution and left to equilibrate over 1 week.

3.5.1. Explicit reversibility

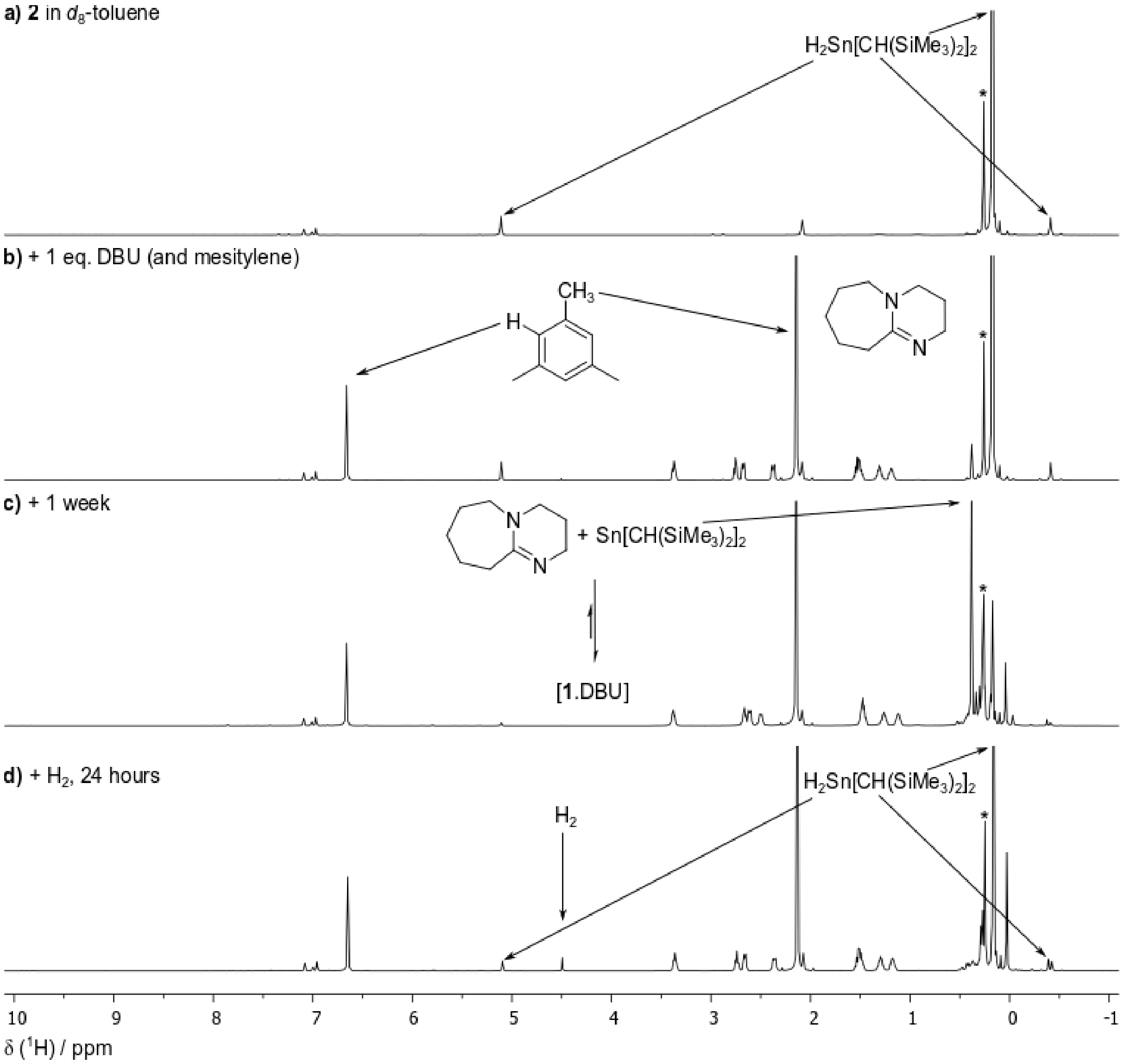

Figure S8 – Series of ^{1}H NMR spectra depicting **a)** a solution of **2** in d_8-toluene (0.06 M) **b)** DBU (1 equivalent) and with mesitylene (2%) added to the solution; **c)** the solution after having been degassed three times over the course of one week, showing the formation of **1**; **d)** reformation of **2** after the addition of H_2 (4 bar). * - small amount of silicone grease from the independent synthesis of **2**.

3.6. H_2 activation with DMAP

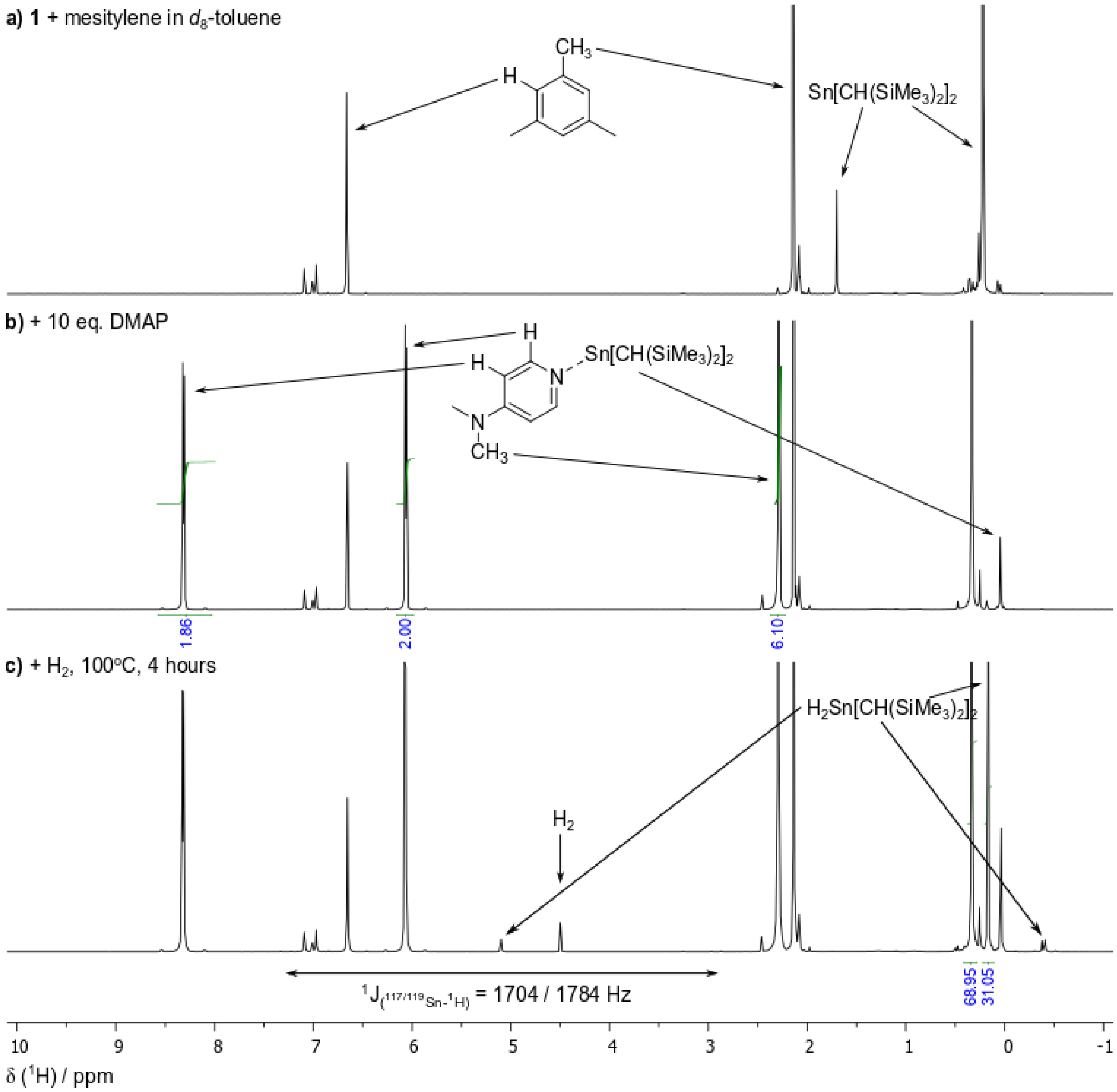

Figure S9 – Series of ^{1}H NMR spectra depicting **a)** a solution of **1** in d_8-toluene (0.06 M) with mesitylene (2%); **b)** DMAP (10 equivalents) added to the solution; **c)** H_2 (4 bar) admitted to the same solution and reacted at 100°C for 4 hours, to 31% completion. Further heating did not increase the extent of reaction.

3.7. Determination of equilibrium constant for H_2 activation with DBU, K_{eq}

$$\mathbf{1}\text{-DBU} + H_2 \xrightleftharpoons{K_{eq}} \mathbf{2} + \text{DBU}$$

$$K_{eq} = \frac{[\mathbf{2}][\text{DBU}]}{[\mathbf{1} \cdot \text{DBU}][H_2]}$$

J. Young's tap NMR tube prepared as outlined in 3.1, with 0.03 mmol **1**, 0.5 equivalents of DBU (0.015 mmol), 0.5 mL d_8-toluene, and H_2 admitted to 1 bar at RT. [1]

Relative integration of the respective ($\underline{Me_3}Si)_2CH$ resonances in the ^1H NMR spectrum provided a ratio of **2**:[**1**·DBU] at equilibrium: 17.21:1.00 (experiment 1); 17.72:1.00 (experiment 2).

Therefore $K_{eq} = 164 \pm 5$, corresponding to $\Delta G = –3.0$ kcal mol^{-1} at 293K.

3.7.1. NMR spectra of 1 / DBU equilibrium

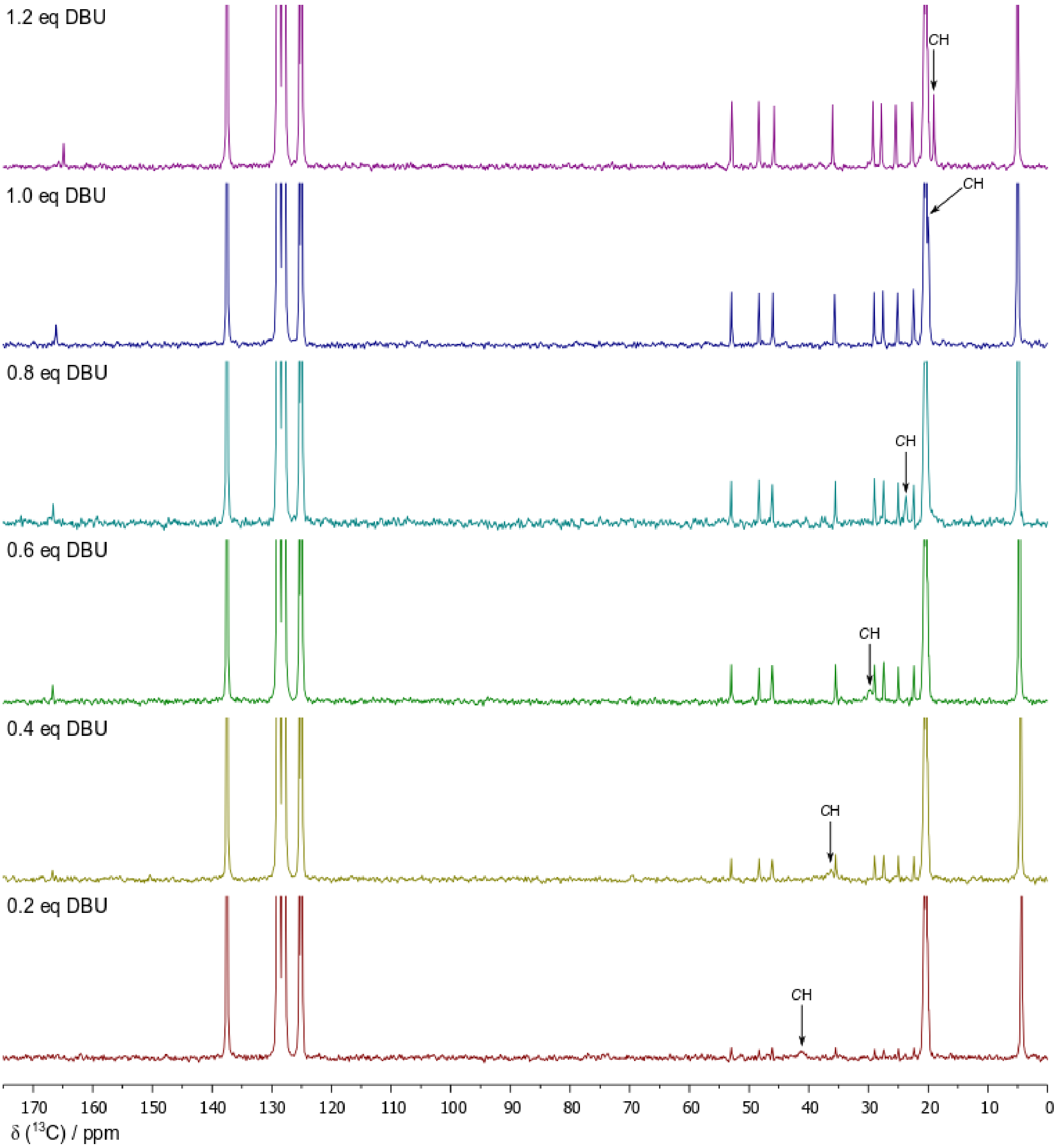

Figure S10 – Change in methine resonance, shown by $^{13}C\{^{1}H\}$ NMR spectroscopy as DBU is added incrementally to a solution of **1**. The limiting shifts of pure **1**, [**1**]$_2$, and **1**·DBU are +60.0 ppm, +28.7 ppm, and +18.5 ppm, respectively.[4] At catalytically relevant concentrations of DBU (< 0.6 eq), the shift of the methine peak is > 28.7 ppm. There must, therefore, be free monomer **1** in solution.

3.7.2. Variable temperature UV-Visible spectroscopy of 1 / DBU equilibrium

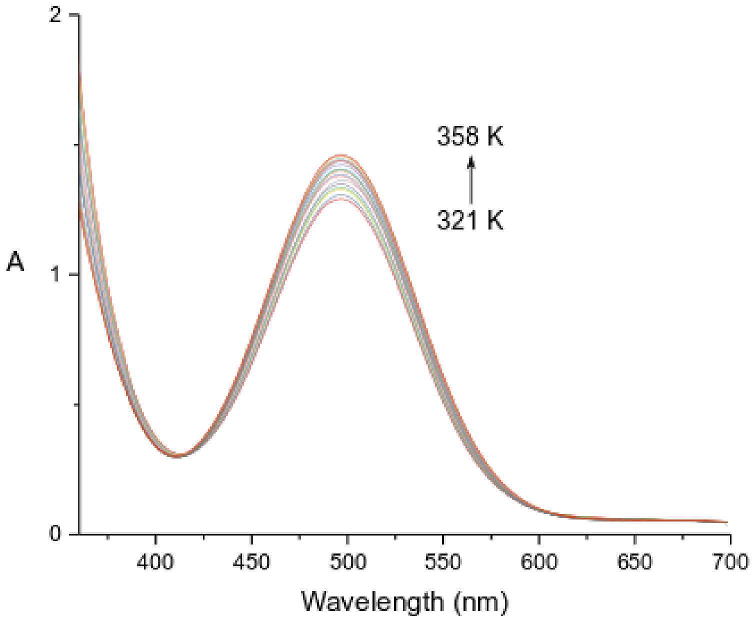

Figure S11 – VT-UV spectra of a 0.003 M solution of **1**/$\mathbf{1}_2$, containing 0.0015 M DBU, from 320.65 K to 358.15 K. Spectra acquired every 2.5 K.

Extinction coefficient of λ = 495 nm peak (due to **1**), ε = 848 M^{-1} cm^{-1}. Previously determined by Lappert *et al.* to be ε = 610 M^{-1} cm^{-1}; however, this does not account for the concentration dependence of **1** due to the dimerisation equilibrium: **1** ↔ 0.5·$[\mathbf{1}]_2$.[1]

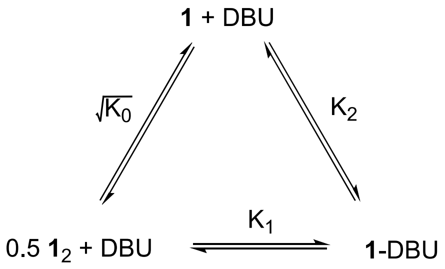

$$K_0 = \frac{[\mathbf{1}]^2}{[\mathbf{1}_2]}$$

$$K_1 = \frac{[\mathbf{1}\cdot \mathrm{DBU}]}{[\mathbf{1}_2]^{0.5}[\mathrm{DBU}]}$$

$$K_2 = \frac{[\mathbf{1}\cdot \mathrm{DBU}]}{[\mathbf{1}][\mathrm{DBU}]}$$

Scheme S1 – Scheme depicting the three equilibria present in solutions of **1** and DBU, and the relations between the concentrations of the species.

Total concentration of "R_2Sn" units in solution, [Tot], can be related to the concentrations of monomer, **1**, and dimer, $\mathbf{1}_2$, in solution by:

$$[\mathrm{Tot}] = [\mathbf{1}] + 2[\mathbf{1_2}] = [\mathbf{1}] + 2\frac{[\mathbf{1}]^2}{\mathrm{K}_0}$$

Table S1 – Relationships between the concentrations of **1**, the total amount of stannylene, **1**·DBU, and DBU in the UV solutions.

Species	**1**	Total	**1** · DBU	DBU
Initial concentration	$[\mathbf{1}]_0$	$[\mathrm{Tot}]_0$	0	$[\mathrm{DBU}]_0$
Change in concentration	$-[\mathbf{1}]_0 + [\mathbf{1}]_\mathrm{f}$	$-[\mathrm{Tot}]_0 + [\mathrm{Tot}]_\mathrm{f}$	$+[\mathrm{Tot}]_0 - [\mathrm{Tot}]_\mathrm{f}$	$-[\mathrm{Tot}]_0 + [\mathrm{Tot}]_\mathrm{f}$
Final concentration	$[\mathbf{1}]_\mathrm{f}$	$[\mathrm{Tot}]_\mathrm{f}$	$+[\mathrm{Tot}]_0 - [\mathrm{Tot}]_\mathrm{f}$	$[\mathrm{DBU}]_0 - [\mathrm{Tot}]_0 + [\mathrm{Tot}]_\mathrm{f}$

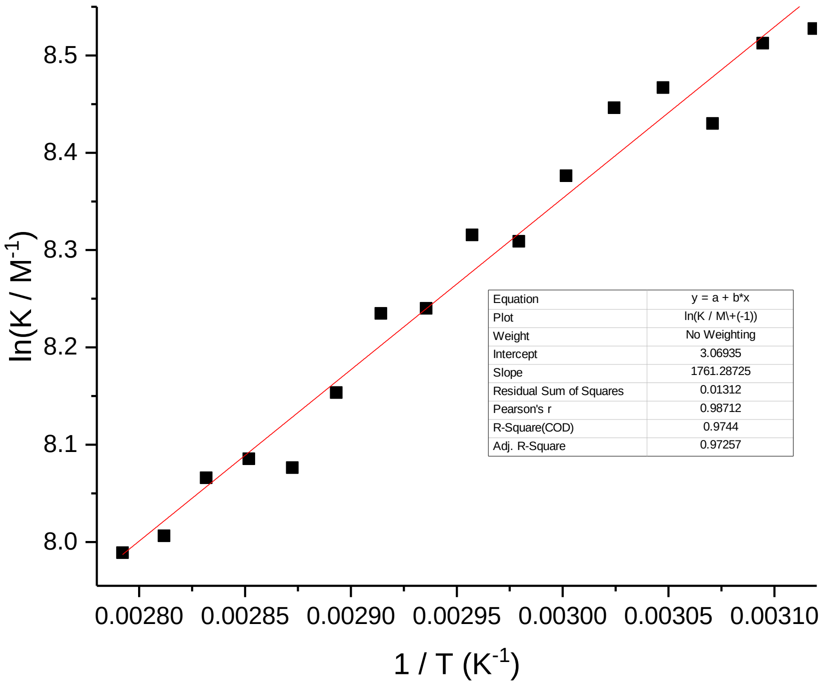

Figure S12 – van't Hoff analysis of VT-UV spectra.

The van't Hoff analysis of the VT-UV spectra gives thermodynamic values of ΔH_2 = -3.50 ± 0.15 kcal mol^{-1} and ΔS_2 = +6.1 ± 0.4 cal mol^{-1} K^{-1}, corresponding to ΔG_2 = -5.3 ± 0.2 kcal mol^{-1} at 293 K. Since ΔG_0 has been previously calculated to be -3.0 kcal mol^{-1},[4] and $\Delta G_1 = 0.5 \times (\Delta G_0) + \Delta G_2$

ΔG_1 = -3.7 ± 0.2 kcal mol^{-1} at 293 K (compared to the computational result of -3.4 kcal mol^{-1}), and corresponding to an equilibrium constant for adduct formation: K_1 ~ 600 $M^{-\frac{1}{2}}$.

4. Kinetic Analysis

All experiments were conducted as specified in Section 3.1. **1** (13.1 mg, 0.03 mmol) was dissolved in d_8-toluene (0.5 mL) in an NMR tube fitted with a J. Young's valve, with mesitylene (10 µL) as an internal standard, and a specified amount of base was added. H_2 was added *via* a freeze-pump-thaw method (1 bar at -196 ºC, *ca*. 4 bar at RT). All signals were manually integrated, and the error found to be approx. ±2% by repeated integration. The amount of stannylene ([Tot]) in solution was found from the integration of the methine peak at approximately 1.7 ppm. Integration values were referenced to the integral of the aromatic C-H peak of mesitylene, the internal standard. Upon admission of H_2 to the NMR tube, the sample was not shaken until just before a ^{1}H spectrum was taken (Bruker AV-400 NMR – 16 scans). T = 0 was counted from the moment the sample was inserted into the spectrometer, and spectra were acquired every 5-10 minutes for up to ~3 hours, or until no more stannylene could be observed. Between spectra, the samples were removed from the spectrometer and shaken regularly, to maintain H_2 concentration in solution. Stannylene concentration ([Tot]) was normalised to the starting concentration (0.06 M) in the T = 0 spectrum.

4.1. Order in base

The method of time scale normalisation was used to find the order in base. Kinetic experiments were performed with solutions containing differing Et_3N concentrations (equivalents to **1**) of 0.054 M (0.95 eq), 0.21 M (3.5 eq), 0.33 M (5.5 eq), 0.48 M (8.0 eq), 0.60 M (10 eq). Plotting the total stannylene ("R_2Sn") concentration ([Tot]) against t•$[Et_3N]^x$ gives overlapping traces if and only if x = reaction order in Et_3N. This occurs when x = 1.

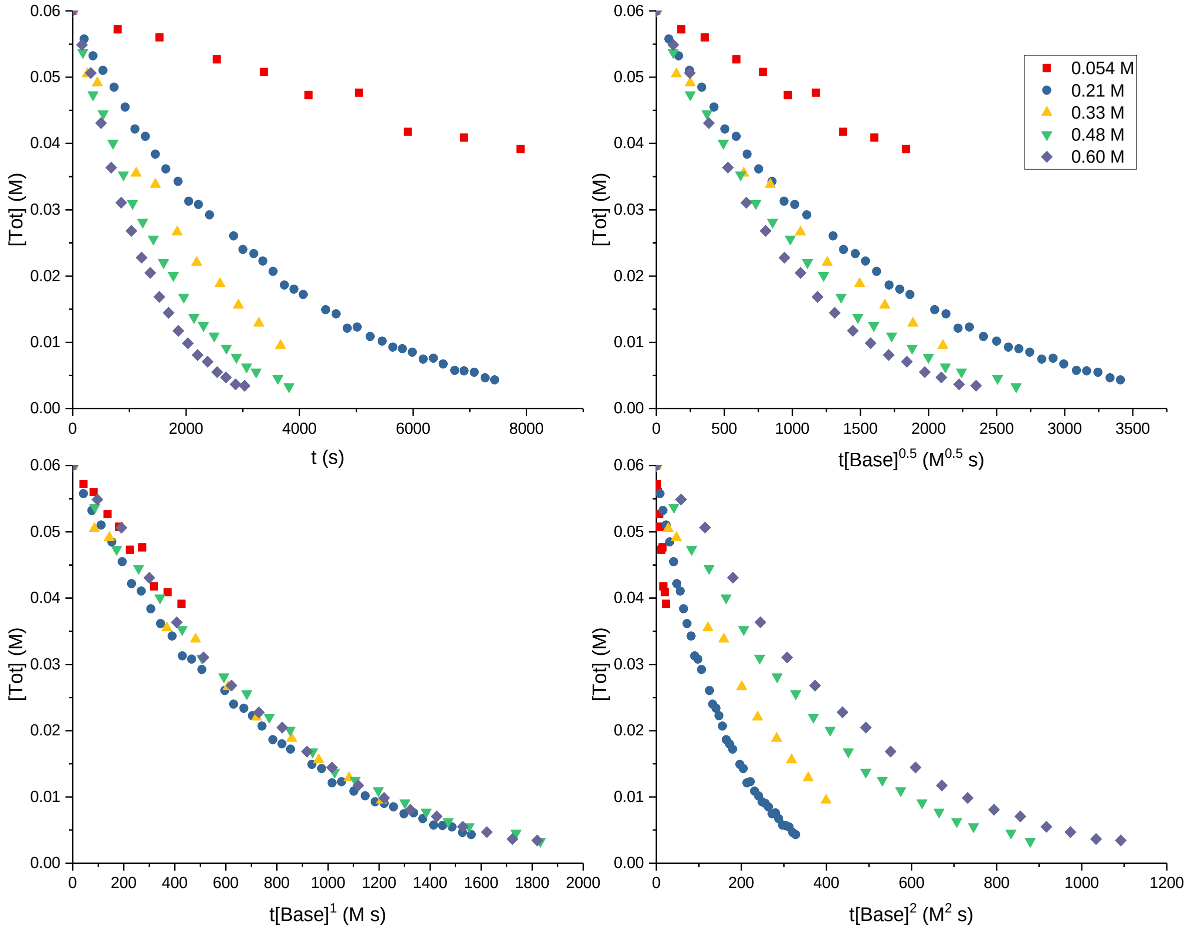

Figure S13 – Stannylene concentrations against normalised timescales. All traces overlap only when the timescale is $t[Et_3N]^1$.

4.2. Mathematical treatment

In order to find rate a constant for the base-catalysed addition of H_2 to stannylene, a linearisation is required. The steps to obtain the desired expression are outlined below (B is a non-coordinating base).

The rate of formation and loss of the encounter complex, enc, can be expressed as a rapid pre-equilibrium:

$$k_1[\mathbf{1}][\mathrm{B}] = k_{-1}[enc]$$

Therefore:

$$[enc] = \frac{k_1}{k_{-1}}[\mathbf{1}][\mathrm{B}]$$

As rate of formation of product can be expressed as:

$$\frac{d[\mathbf{2}]}{d\mathrm{t}} = k_2[enc][\mathrm{H_2}]$$

Combining the above two expressions gives:

$$\frac{d[\mathbf{2}]}{d\mathrm{t}} = \frac{k_1 k_2}{k_{-1}}[\mathbf{1}][\mathrm{B}][\mathrm{H_2}]$$

However, by NMR, we cannot easily measure $[\mathbf{1}]$ or the dimer $[(\mathbf{1})_2]$ directly, as they are in fast equilibrium.[3] We can see the total concentration of "$\mathrm{R_2Sn}$" species in solution, $[\mathrm{Tot}]$, as either monomer or dimer, which can be related to the concentrations of the monomer and dimer by:

$$[\mathrm{Tot}] = [\mathbf{1}] + 2[(\mathbf{1})_2]$$

Due to the dimerisation equilibrium of $\mathrm{R_2Sn}$, which can be expressed as:

$$\mathrm{K_0} = \frac{[\mathbf{1}]^2}{[(\mathbf{1})_2]}$$

Combining the above two equations gives:

$$[\mathrm{Tot}] = [\mathbf{1}] + \frac{2[\mathbf{1}]^2}{\mathrm{K_0}}$$

Solving for $[\mathbf{1}]$ gives:

$$[\mathbf{1}] = \frac{1}{4}\left(\sqrt{\mathrm{K_0}}\sqrt{8[\mathrm{Tot}] + \mathrm{K_0}} - \mathrm{K_0}\right)$$

This provides the rate of reaction as:

$$\frac{d[\mathbf{2}]}{d\mathrm{t}} = \frac{k_1 k_2}{4k_{-1}}\left(\sqrt{\mathrm{K_0}}\sqrt{8[\mathrm{Tot}] + \mathrm{K_0}} - \mathrm{K_0}\right)[\mathrm{B}][\mathrm{H_2}]$$

As the base is catalytic its concentration does not change, and the amount of H_2 is sufficiently high that it should not deplete over the course of the reaction, this expression can be simplified to:

$$\frac{d[\mathbf{2}]}{dt} = \frac{k^*}{4}\left(\sqrt{K_0}\sqrt{8[\mathrm{Tot}] + K_0} - K_0\right)$$

Where:

$$k^* = \frac{k_1 k_2}{k_{-1}}[\mathrm{B}][\mathrm{H_2}]$$

Alternatively, in terms of rate of loss of reactant:

$$-\frac{d[\mathrm{Tot}]}{dt} = \frac{k^*}{4}\left(\sqrt{K_0}\sqrt{8[\mathrm{Tot}] + K_0} - K_0\right)$$

Rearrangement and integration by substitution (see below) gives:

$$\sqrt{\frac{8[\mathrm{Tot}]}{K_0} + 1} + \ln\left(\frac{\sqrt{K_0 + 8[\mathrm{Tot}]} - \sqrt{K_0}}{\sqrt{K_0 + 8[\mathrm{Tot}]_0} - \sqrt{K_0}}\right) - \sqrt{\frac{8[\mathrm{Tot}]_0}{K_0} + 1} = -k^* t$$

Therefore, plotting $\sqrt{\frac{8[\mathrm{Tot}]}{K_0} + 1} + \ln\left(\frac{\sqrt{K_0+8[\mathrm{Tot}]}-\sqrt{K_0}}{\sqrt{K_0+8[\mathrm{Tot}]_0}-\sqrt{K_0}}\right)$ against t should give a straight line of gradient $-k^*$, which is indeed observed.

Integration:

$$\int_{[\mathrm{Tot}]_0}^{[\mathrm{Tot}]} \frac{1}{\left(\sqrt{K_0}\sqrt{8[\mathrm{Tot}] + K_0} - K_0\right)} d[\mathrm{Tot}] = \int_0^{t} -\frac{k^*}{4} dt$$

Make substitution:

$$u = 8[\mathrm{Tot}] + K_0$$

So:

$$\int_{u_0}^{u} \frac{1}{8\sqrt{K_0}\left(\sqrt{u} - \sqrt{K_0}\right)} du = \int_0^{t} -\frac{k^*}{4} dt$$

Make substitution:

$$v = \sqrt{u} - \sqrt{K_0}$$

So:

$$\int_{v_0}^{v} \left(\frac{1}{4\sqrt{K_0}} + \frac{1}{4v}\right) dv = \int_0^{t} -\frac{k^*}{4} dt$$

Which is integrated to provide the given equation above.

4.2.1. Determination of Et_3N rate constant, $k'_{(Et3N)}$

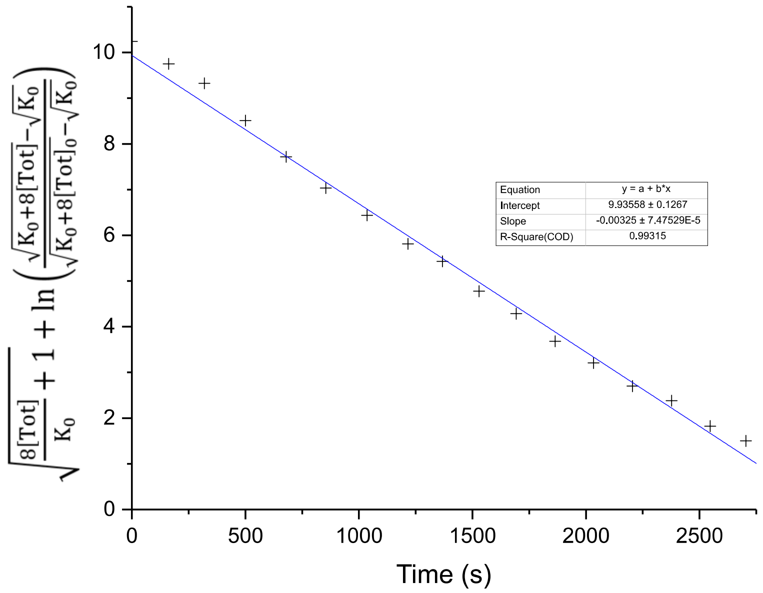

Figure S14 – Linearisation of kinetic data for reaction with 10 eq. Et_3N

Gradient of linear fit: $-k^*$ = -0.00325 ± 0.00007 s^{-1} (Error from regression analysis).

Weighted average of 4 runs: k^* = 0.00322 ± 0.00005 s^{-1}.

[Et_3N] = 0.600 ± 0.015 M (Error in measured volume approx. 2.5%).

pH_2 = 4.0 ± 0.2 bar (Error estimated from pH_2 when admitted at 77 K: pH_2(77 K) = 1.0 bar; pH_2(293 K) = 3.8 bar. pH_2(77 K) = 1.1 bar; pH_2(293 K) = 4.2 bar).

[H_2] = 0.0114 ± 0.0006 M.[3]

Therefore $k'_{(Et3N)}$ = 0.47 ± 0.03 M^{-2} s^{-1}.

4.2.1.1. Determination of kinetic isotope effect, KIE

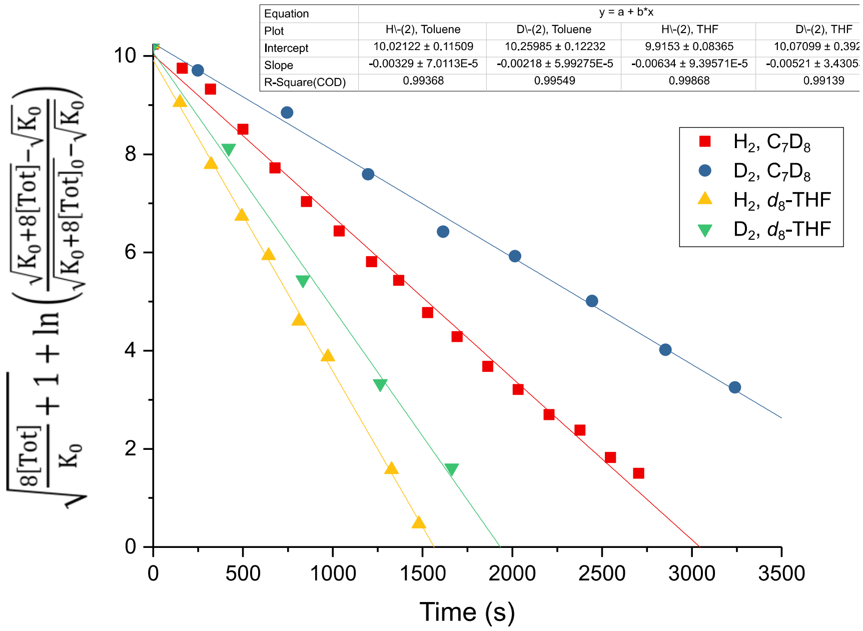

Figure S15 – Linearisation of kinetic data for reactions of **1** with H_2 and D_2 in both d_8-toluene and d_8-THF (all mediated by 10 eq. Et_3N).

In toluene:

Gradient of linear fit: $-k_{H2}{}^{*}$ = -0.00322 ± 0.00005 s^{-1} (weighted average of 4 runs)

Gradient of linear fit: $-k_{D2}{}^{*}$ = -0.00213 ± 0.00004 s^{-1}

$$\mathrm{KIE}_{(\mathrm{Toluene})} = \frac{k_{H2}{}^{*}}{k_{D2}{}^{*}} = 1.51 \pm 0.04$$

In THF:

Gradient of linear fit: $-k_{H2}{}^{*}$ = -0.00634 ± 0.00009 s^{-1}

Gradient of linear fit: $-k_{D2}{}^{*}$ = -0.0052 ± 0.0003 s^{-1}

$$\mathrm{KIE}_{(\mathrm{THF})} = \frac{k_{H2}{}^{*}}{k_{D2}{}^{*}} = 1.22 \pm 0.08$$

4.2.1.2. Comparison of toluene and THF rate constants

$k_{H2}{}^{*}{}_{(\mathrm{toluene})}$ = 0.00322 ± 0.00005 s^{-1}

$k_{H2}{}^{*}{}_{(\mathrm{THF})}$ = 0.00634 ± 0.00009 s^{-1}

$$\text{Relative rate in THF} = \frac{k_{H2}{}^{*}{}_{(\mathrm{THF})}}{k_{H2}{}^{*}{}_{(\mathrm{toluene})}} = 1.97 \pm 0.04$$

4.2.2. Determination of TBTMG rate constant, *k*’(TBTMG)

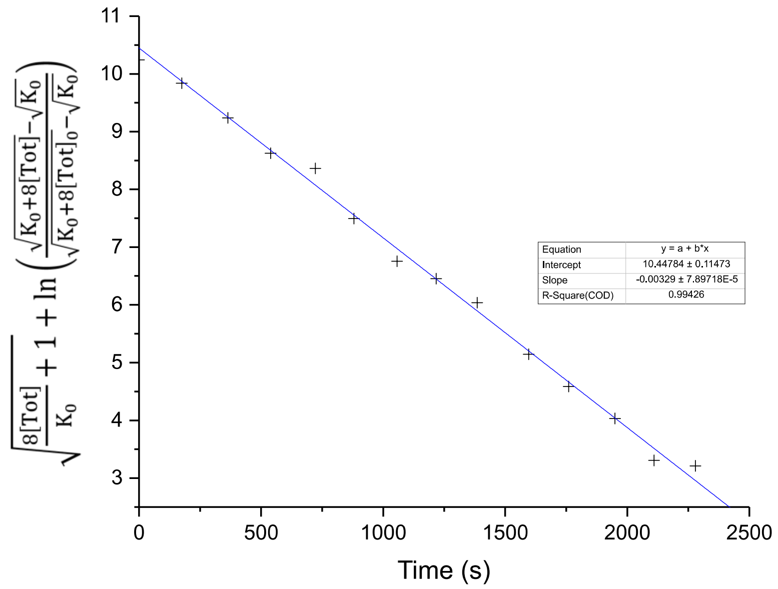

Figure S16 – Linearisation of kinetic data for reaction with 1 eq. TBTMG.

Gradient of linear fit: $-k^*$ = -0.00334 ± 0.00007 s^{-1} (Error from regression analysis).

Weighted average of 2 runs: k^* = 0.00341 ± 0.00005 s^{-1}.

[TBTMG] = 0.06 ± 0.002 M (Error in measured volume approx. 3%).

pH_2 = 4.0 ± 0.2 bar (Error from pH_2 when admitted at 77 K: pH_2(77 K) = 1.0 bar; pH_2(293 K) = 3.8 bar. pH_2(77 K) = 1.1 bar; pH_2(293 K) = 4.2 bar).

[H_2] = 0.0114 ± 0.0006 M.[3]

Therefore $k'_{(TBTMG)}$ = 5.0 ± 0.3 M^{-2} s^{-1}.

4.2.3. Determination of PMP rate constant, *k*'$_{(PMP)}$

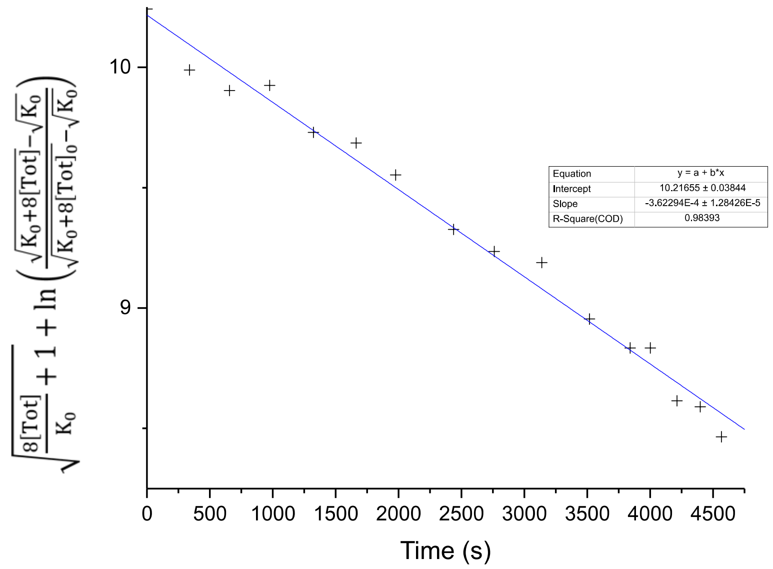

Figure S17 – Linearisation of kinetic data for reaction with 20 eq. PMP.

Gradient of linear fit: $-k^{*}$ = -0.000362 ± 0.000013 s^{-1} (Error from regression analysis).

Weighted average of 2 runs: k^{*} = 0.00341 ± 0.00005 s^{-1}.

[PMP] = 1.20 ± 0.04 M (Error in measured volume approx. 3%).

pH_2 = 4.0 ± 0.2 bar (Error from pH_2 when admitted at 77 K: pH_2(77 K) = 1.0 bar; pH_2(293 K) = 3.8 bar. pH_2(77 K) = 1.1 bar; pH_2(293 K) = 4.2 bar).

[H_2] = 0.0114 ± 0.0006 M.[3]

Therefore *k*'$_{(PMP)}$ = 0.0266 ± 0.0018 M^{-2} s^{-1}.

5. Computational details

In our present computational study we used the following protocol:

Conformational analysis: Due to the conformational complexity of the investigated structures (e.g. various rotational degrees of freedom associated with the $(Me_3Si)_2CH$ group, or the relative position and orientation of the reactants), we carried out an extensive conformational analysis for each species (reactants, intermediates, products and transition states). The conformational space was first explored via an initial Monte-Carlo (MC) search using an empirical force field (slightly modified OPLS_2005), which was then followed by density functional theory (DFT) calculations. The force field modification concerns the partial atomic charges used to estimate the energy contribution of Coulomb interactions. These parameters were obtained from DFT calculations (CHELPG atomic charges[5]). Due to the lack of OPLS_2005 force field parameters for Sn atom, we used the parameters of O atom in cases with two ligands, and parameters of N atom for those of three or four ligands, and we constrained the initial Sn-C atomic distances (obtained from DFT). The MC conformational search was carried out using the *MacroModel* software.[6]

Geometry optimisations: The most favored conformers obtained in the Monte Carlo conformational search were subjected to DFT calculations. The geometry optimisations (including transition state searches as well) were carried out at the ωB97X-D/Def2SVP level of theory.[7,8] Ultra-fine integration grid was employed in all electronic structure calculations. The nature of the stationary points was characterised via vibrational analysis. From transition state structures, we followed intrinsic reaction coordinate (IRC) pathways in both forward and reverse directions, and we identified the related intermediates accordingly.

Single point electronic energy calculations: For each optimised structure (potential energy minimum or transition state), additional single-point energy calculations were performed with the larger Def2TZVPP basis set.[8]

Entropic and thermal contributions: Harmonic frequencies were computed at the ωB97X-D/Def2SVP level. These data were used to estimate the zero-point energies as well as the thermal and entropic contributions to the Gibbs free energies. The thermochemical data were obtained within the ideal gas – rigid rotor – harmonic oscillator approximation for T = 298.15 K and p = 1 atm (ideal gas standard state), but corrections were applied for switching to c = 1 mol dm^{-3} concentrations (0.003019 a.u.).

Solvent effects: Solvent effects were also taken into account by estimating the solvation free energies by using the integral equation formalism variant of the polarisable continuum model (IEFPCM).[9] The atomic radii and non-electrostatic terms in the IEFPCM calculations were those introduced by Truhlar and coworkers (SMD solvation model).[10] Solvation free energies (for toluene as a solvent) calculated at the ωB97X-D/Def2SVP level for the gas-phase optimised geometries.

The energy values reported in the paper correspond to solution phase Gibbs free energies that are based on *ω*B97X-D/Def2TZVPP electronic energies and all additional terms obtained at the ωB97X-D/Def2SVP level (see Table 2 in section 5.2.4.). All DFT calculations were carried out with the *Gaussian09* software.[11]

5.1. Computational results

DFT computations were aimed at exploring the mechanism of base-assisted H_2 activation with stannylene $[(Me_3Si)_2CH]_2Sn$ (**1**) and to compare the reactivity of various bases for this process (Scheme S2). We have explored the full reaction pathway in details for base = Et_3N and examined the H_2 splitting step for other bases. The results are presented below.

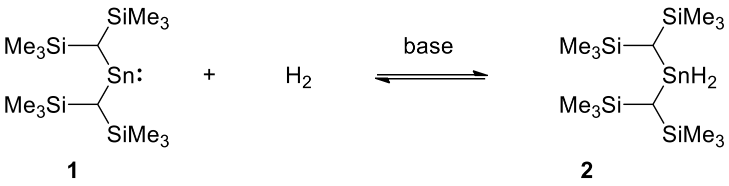

Scheme S2: Reaction examined computationally.

5.1.1. Dimerisation of stannylene, 1

As noted in the main text, Lappert's stannylene **1** dimerises in solution and in solid state as well (Scheme S3). Computations predict ΔG = -1.9 kcal mol^{-1} for the dimerization, i.e. the equilibrium is predicted to be shifted towards the dimeric form (in solution), which is in line with experimental observations.

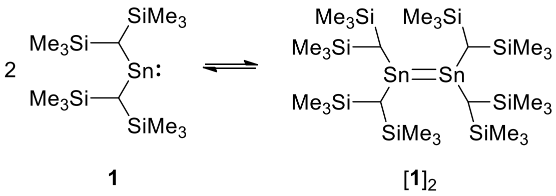

Scheme S3: Dimerisation of **1**.

The most stable form of $[\mathbf{1}]_2$ obtained via the conformational analysis is 2.1 kcal mol^{-1} more favored than that corresponding to the X-ray structure. The two structures differ in the orientation of the $CH(SiMe_3)_2$ groups as illustrated in Figure S18. These groups can easily rotate around the Sn-C bond in the monomeric form allowing fast isomerisation at room temperature.

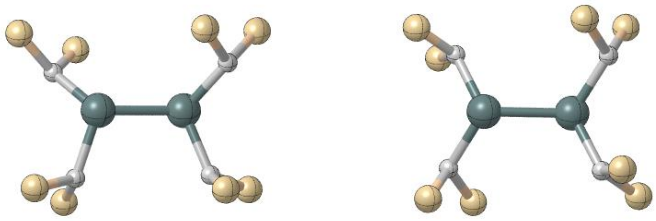

$[\mathbf{1}]_2^{conf}$ (0.0) $[\mathbf{1}]_2^{X\text{-}ray}$ (+2.1)

Figure S18: Two different isomers of $[\mathbf{1}]_2$. All CH_3 units of the $CH(SiMe_3)_2$ groups are omitted for clarity.

5.1.2. H_2 addition to 1 in the absence of base

The addition of H_2 to **1** is predicted to be exergonic by 7.7 kcal mol^{-1}. Herein, the reactant state is defined as 0.5·[**1**]$_2$ + H_2 (rather than **1** + H_2) to take into account the dimerisation equilibrium. The transition state identified for this process in the absence of base is 46.0 kcal mol^{-1} above the reactant state (Figure S19), implying that the reaction is significantly hindered from a kinetic standpoint. The high barrier can be associated with the large spatial extent of the Sn frontier orbitals, which does not allow favorable overlap with the σ and σ* orbitals of H_2 (as opposed to singlet carbenes, for instance).

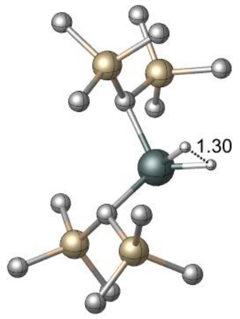

TS$_{HH}$ (+46.0)

Figure S19: Transition state of H_2 splitting by stannylene **1**. Relative stability (in parenthesis; in kcal mol^{-1}) was computed with respect to 0.5·[**1**]$_2$ + H_2; H-H bond distance is in Å.

5.1.3. H_2 addition with the assistance of Et_3N

For the mechanism of base-assisted H_2 addition to **1** we envisaged that the monomeric form of the stannylene and the base can form a frustrated Lewis pair (FLP) and cleave H_2 heterolytically. Analogous reactivity with the [**1**]$_2$ dimer is quite unlikely, because the Sn centers of [**1**]$_2$ are shielded sterically via the methyl groups, preventing approach of the base to a distance characteristic of a LA·LB encounter complex.

For the **1**/Et_3N pair, no datively bound complexes could be identified computationally, but weak association between the components was found. These weakly bound van der Waals complexes are thermodynamically unstable (they are 6-8 kcal mol^{-1} above the separated components in free energy), but the conformers with properly oriented acid/base sites are highly reactive toward H_2. Computations indicate that the **1**/Et_3N pair can indeed act as an FLP and induce H_2 heterolysis. The most favored transition state found for H_2 splitting (**TS$_1$** in Figure S20) represent a barrier of 20.4 kcal mol^{-1} relative to 0.5·[**1**]$_2$ + Et_3N + H_2, which is consistent with the observed reaction rate. The subsequent ion-pair intermediate **int$_1$** is rather high-lying, but it can easily rearrange via **TS$_2$** to another form. In intermediate **int$_2$**, the proton is well prepared for the delivery to Sn, which occurs via **TS$_3$** and it gives the addition product **2**.

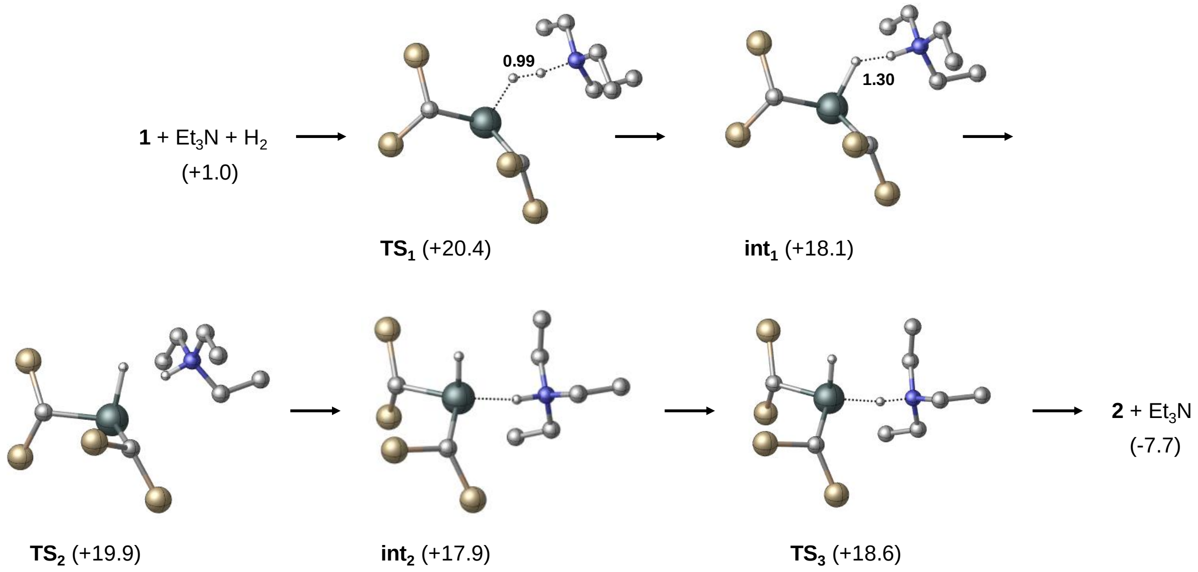

Figure S20: Stationary points identified for the Et_3N-assisted H_2 activation with **1**. Relative free energies are with respect to the 0.5·**[1]**$_2$ + Et_3N + H_2 state (in kcal mol^{-1}). Selected bond distances are in Å. The Me groups of the stannylene are omitted for clarity.

The free energy profile of the reaction sequence is shown in Figure S21. These results point to a 3-step mechanism, however, the involved ion-pair species (**int$_1$** and **int$_2$**) can be regarded as transient (short-lived) intermediates. Although the located transition states are energetically close-lying, the overall reaction barrier appears to be determined by the H_2 splitting step (by **TS$_1$**), which is in line with the results of kinetic measurements.

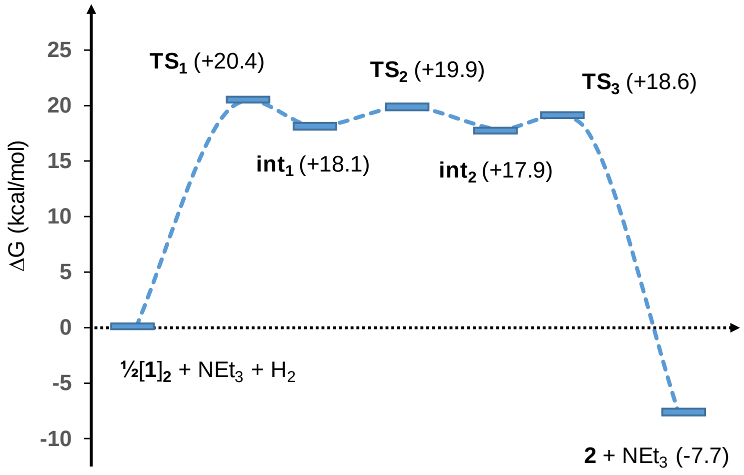

Figure S21: Computed free energy profile for the Et_3N-assisted H_2 activation with **1**. Relative free energies are with respect to the 0.5·**[1]**$_2$ + Et_3N + H_2 state (in kcal mol^{-1}).

The free energy profile was also computed for the more polar solvent THF. As expected from the ionic nature of the reaction intermediates, the reaction is predicted to be kinetically more favored in THF as compared to that in toluene (see Figure S22). The overall barriers of the two reactions differ by

2.1 kcal mol^{-1}, which qualitatively agrees with the measured rates. The overestimation of the observed effect could be related to the polarisable continuum solvent model (specific **1**-THF interactions, especially with large excess of THF, may result in reactant state stabilisation leading to slightly larger reaction barrier).

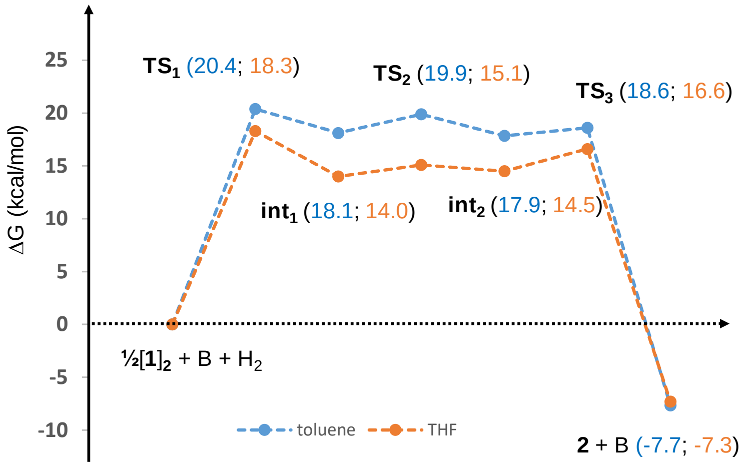

Figure S22: Comparison of free energy profiles for two different solvents (toluene and THF).

5.1.4. H_2 addition with the assistance of DBU

The reaction pathway has been explored for the **1**/DBU pair as well. The identified stationary points are depicted in Figure S23, and the computed free energy profile is shown in Figure S24.

DBU is found to form a datively bound complex with **1**, which is predicted to be 3.4 kcal/mol more stable than the reactant state (0.5·**[1]**$_2$ + DBU + H_2). The most favored transition state found for H_2 splitting (**TS$_1$** in Figure S23) lies at 17.0 kcal/mol in free energy, however, the overall barrier is 20.4 kcal/mol, which is consistent with the observed reaction rate. Similarly to the reaction with **1**/NEt_3, the H_2-activation process occurs in 3 elemantary steps, but the ion-pair intermediates (**int$_1$** and **int$_2$**) as well as the related transition states (**TS$_2$** and **TS$_3$**) are predicted to be well below **TS$_1$**. Consequently, the H_2 splitting step can be clearly classified as the rate-determining step of the reaction sequence.

Reactant state stabilisation arising from the dative bond formation (complex **1**-DBU) implies that the overall reaction becomes less exergonic, and in fact reversible. These results are in line with our experimental observations.

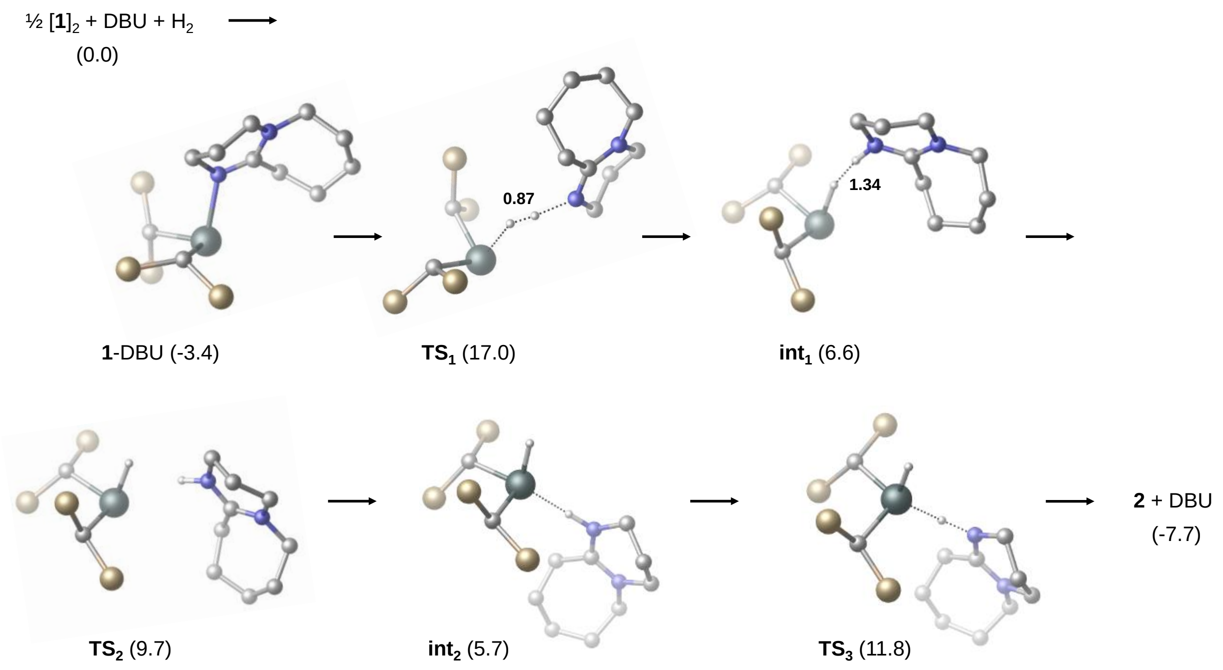

Figure S23: Stationary points identified for the DBU-assisted H_2 activation with **1**. Relative free energies are with respect to the 0.5·**[1]**$_2$ + DBU + H_2 state (in kcal/mol). Selected bond distances are in Å. The Me groups of the stannylene are omitted for clarity.

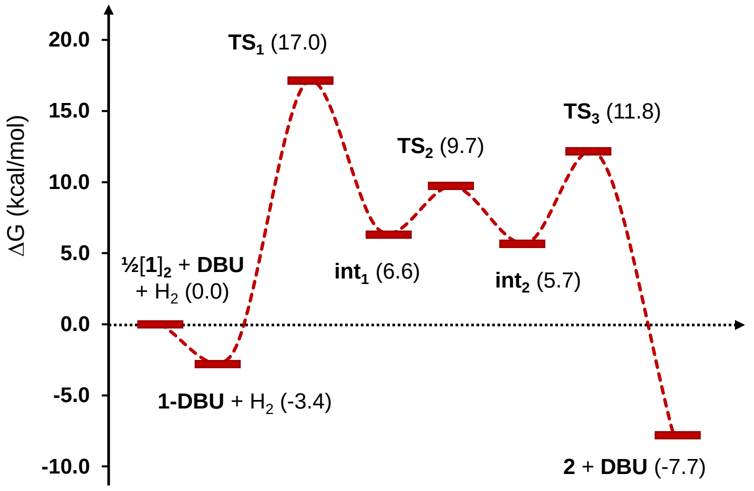

Figure S24: Computed free energy profile for the DBU-assisted H_2 activation with **1**. Relative free energies are with respect to the 0.5·**[1]**$_2$ + DBU + H_2 state (in kcal/mol).

5.1.5. H_2 addition with other bases

Transition states for the base-assisted H_2 splitting step (**TS₁**) and the related intermediates (**int₁**) were computed for additional bases (base = **DBU**, **DMAP**, **PMP**, **TBTMG** (Barton's base) – see Figure S25). We have also examined the possibility of dative bond formation between **1** and the base and found this feasible only for **DBU** and **DMAP**. In these cases, the overall barrier of H_2 activation and the overall thermodynamics (free energy of 0.5·[**1**]$_2$ + base + H_2 = **1**-H_2 + base) is influenced by the strength of the **1**-base dative bond. The basicity of base molecules were estimated in terms of proton affinities. The results are summarised in Table S2.

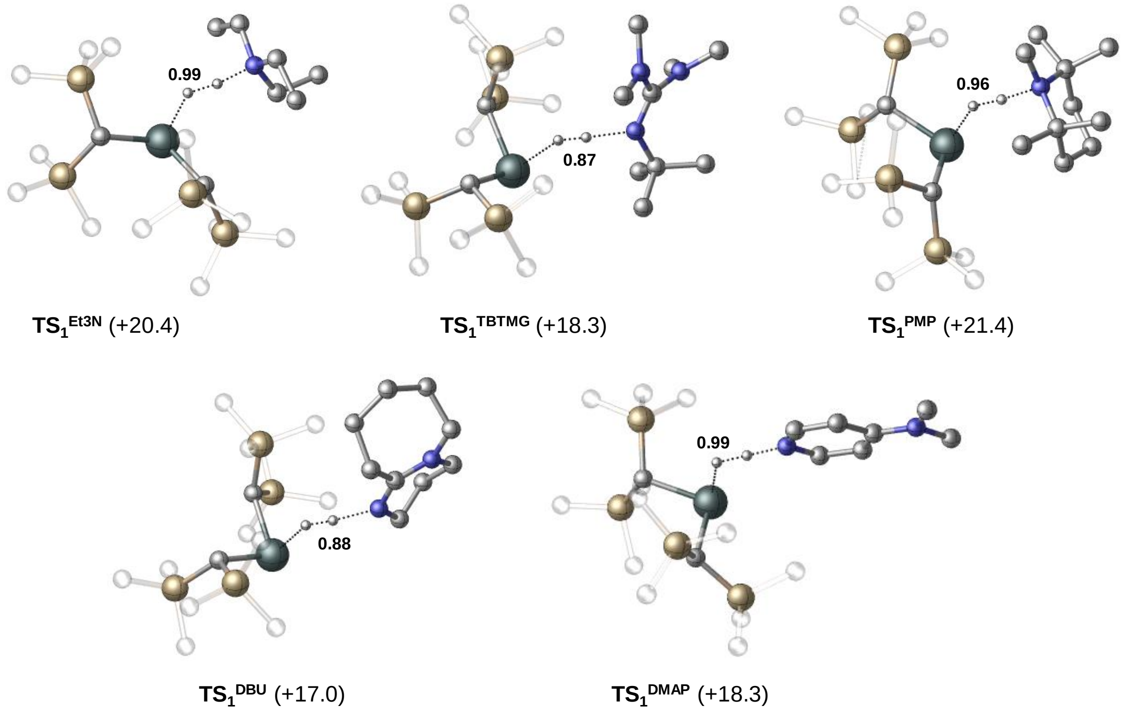

Figure S25: Transition states of H_2 splitting. Relative free energies are with respect to the 0.5·**[1]₂** + base + H_2 state (in kcal mol^{-1}). H-H bond distances are in Å. The Me groups of the stannylene are transparent for clarity.

Table S2: Calculated free energy data for the series of reactions.[a]

Property	Et_3N	TBTMG	PMP	DBU	DMAP
**1·LB**^{a}	-	-	-	-3.4	-3.6
**TS₁**^{a}	20.4	18.3	21.4	17.0	20.1
**int₁**^{a}	18.1	5.4	16.1	6.6	16.8
$\Delta G^{\ddagger\, a}$	20.4	18.3	21.4	20.4	23.7
$\Delta G_{reaction}{}^a$	-7.7	-7.7	-7.7	-4.3	-4.1
**PA** ^{a,b}	-270.1	-286.0	-272.7	-283.5	-272.2
**pK_a** ^{c}	18.8	23.6	18.7	24.3	18.0
**d(HH)** ^{d}	0.99	0.87	0.96	0.88	0.99

^{a}Free energy data are with respect to 0.5·[**1**]$_2$ + base + H_2 (in kcal mol^{-1}); $\Delta G^{\ddagger}$ is activation free energy. ^{b}Proton affinity is defined as the free energy of base + $H^+ \rightarrow$ baseH^+. ^{c}measured in MeCN.[30] ^{d}H-H distance in TS_1 (in Å; 0.76 Å in free H_2).

Comments:

- The overall barriers ($\Delta G^{\ddagger}$ data) are consistent with the measured rates.

- The stability of **int₁** intermediates correlates very well with the proton affinities (see Figure S26), but this is not strictly true for the transition states (steric factors are more important here).

- The H-H distances measured in TS structures are in line with the Hammond principle. These distances are unusually large pointing to late transition states, which is not surprising given that the H_2-splitting products (**int₁** species) are fairly unstable.

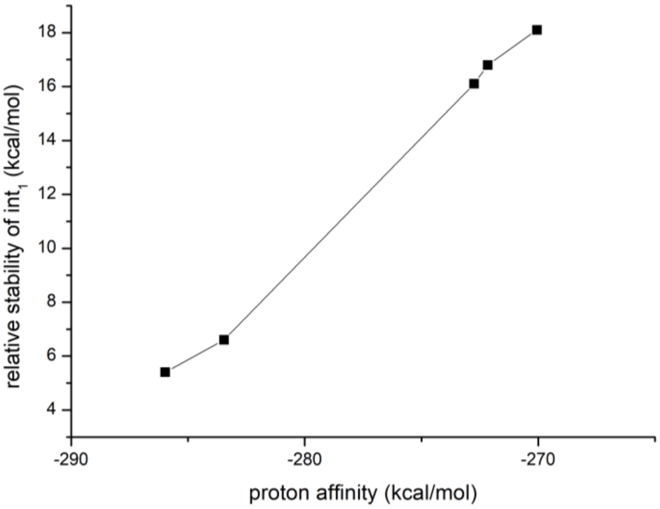

Figure S26: Correlation between the relative stability of **int₁** and PA of the base.

5.2. Total energy data

The total energy data of all species discussed in the main text or SI are collected in Table S3.

Table S3: Total energy data (in a.u.) computed for ωB97X-D/Def2SVP optimised structures.

structures	E_0	$G^0_{298.15\ K}$	$G^{sol}_{toluene}$	E_0'	$G^0_{sol,298.15\ K}$
H_2	-1.1718	-1.1732	-1.1713	-1.1765	-1.1745
**1**	-1927.9825	-1927.5674	-1927.9874	-1929.0188	-1928.6057
[**1**]$_2^{conf}$	-3856.0017	-3855.1391	-3856.0096	-3858.0722	-3857.2145
[**1**]$_2^{X\text{-}ray}$	-3856.0006	-3855.1363	-3856.0078	-3858.0712	-3857.2111
**2**	-1929.1842	-1928.7532	-1929.1884	-1930.2237	-1929.7939
**TS$_{HH}$** (no base)	-1929.0946	-1928.6667	-1929.0992	-1930.1347	-1929.7083
Et_3N	-292.1220	-291.9492	-292.1283	-292.4395	-292.2701
Et_3NH^+	-292.5133	-292.3246	-292.5731	-292.8324	-292.7005
**TS₁**	-2221.2833	-2220.6554	-2221.2960	-2222.6374	-2222.0193
**int₁**	-2221.2850	-2220.6530	-2221.3024	-2222.6406	-2222.0229

structures	E_0	$G^0_{298.15\ K}$	$G^{sol}_{toluene}$	E_0'	$G^0_{sol,298.15\ K}$
**TS_2**	-2221.2796	-2220.6466	-2221.2992	-2222.6366	-2222.0201
**int_2**	-2221.2901	-2220.6554	-2221.3059	-2222.6452	-2222.0234
**TS_3**	-2221.2892	-2220.6588	-2221.3018	-2222.6431	-2222.0222
**DMAP**	-381.8577	-381.7279	-381.8682	-382.2692	-382.1469
**DMAP**H^+	-382.2615	-382.1168	-382.3180	-382.6718	-382.5807
**1-DMAP**	-2309.8773	-2309.3060	-2309.8911	-2311.3204	-2310.7599
**TS_1^{DMAP}**	-2311.0147	-2310.4348	-2311.0322	-2312.4620	-2311.8966
**int_1^{DMAP}**	-2311.0172	-2310.4331	-2311.0402	-2312.4660	-2311.9018
**DBU**	-461.6349	-461.4220	-461.6486	-462.1266	-461.9243
**DBU**H^+	-462.0578	-461.8296	-462.1168	-462.5483	-462.3760
**1-DBU**	-2389.6553	-2389.0009	-2389.6712	-2391.1786	-2390.5370
**TS_1^{DBU}**	-2390.8055	-2390.1380	-2390.8221	-2392.3327	-2391.6789
**int_1^{DBU}**	-2390.8144	-2390.1449	-2390.8393	-2392.3430	-2391.6955
**TS_2^{DBU}**	-2390.8128	-2390.1397	-2390.8373	-2392.3421	-2391.6906
**int_2^{DBU}**	-2390.8190	-2390.1495	-2390.8417	-2392.3467	-2391.6969
**TS_3^{DBU}**	-2390.8147	-2390.1469	-2390.8320	-2392.3407	-2391.6872
**PMP**	-448.0250	-447.7613	-448.0327	-448.5031	-448.2440
**PMP**H^+	-448.4242	-448.1450	-448.4819	-448.9033	-448.6787
**TS_1^{PMP}**	-2377.1883	-2376.4669	-2377.2013	-2378.7030	-2377.9917
**int_1^{PMP}**	-2377.1923	-2376.4684	-2377.2109	-2378.7086	-2378.0001
**TBTMG**	-519.3114	-519.0516	-519.3229	-519.8724	-519.6211
**TBTMGH+**	-519.7426	-519.4653	-519.7977	-520.3020	-520.0769
**TS_1^{TBTMG}**	-2448.4828	-2447.7667	-2448.4954	-2450.0802	-2449.3737
**int_1^{TBTMG}**	-2448.5000	-2447.7772	-2448.5218	-2450.0982	-2449.3943

Notation: E_0 and E_0' refer to electronic energies computed at *ω*B97X-D/Def2SVP and *ω*B97X-D/Def2TZVPP level of DFT; G^0 and G^{sol} denote gas-phase and solution-phase Gibbs free energies obtained from *ω*B97X-D/Def2SVP calculations (T = 298.15 K). The last column is obtained as $G_0^{sol} = E_0' + (G^0 - E_0) + (G^{sol} - E_0) + \Delta G_{conc}$. The relative stabilities discussed in the manuscript and in the SI are obtained from these values. The value ΔG_{conc} corresponds to concentration correction to the free energy when switching from p = 1 atm (ideal gas standard state) to c = 1 mol/dm^3 (standard concentration in solution phase, where ΔG_{conc} = 0.00302 a.u.).

5.3. Cartesian coordinates of the geometry optimised structures

Cartesian coordinates of ωB97X-D/Def2SVP optimised geometries are given below in standard XYZ format (units are in Å). First line indicates total number of atoms; second line is molecule name (as defined in the paper or in the SI).

```
 2
H2
 H    0.000000    0.000000    0.378902
 H    0.000000    0.000000   -0.378902

 57
1
 C   -1.929295   -1.187130   -2.148014
Si   -2.210888    0.522865   -1.405278
 C   -3.909749    0.664968   -0.614527
Si   -5.402161    0.776874   -1.757051
 C   -5.237359   -0.281173   -3.310913
 C   -1.981140    1.847130   -2.728899
 C   -0.861903    0.777080   -0.098311
 C   -5.696704    2.565521   -2.297468
 C   -6.938635    0.184046   -0.833561
Sn   -3.932541    2.365520    0.821508
 C   -5.128074    1.284074    2.356148
Si   -6.408221    2.443033    3.097575
 C   -5.605613    3.616682    4.337146
 C   -7.810985    1.492359    3.924541
 C   -7.195339    3.499734    1.734901
Si   -3.978044    0.375404    3.537761
 C   -4.820980   -0.154885    5.140554
 C   -3.332589   -1.186516    2.695704
 C   -2.491701    1.454819    3.988445
 H   -0.898521   -1.279901   -2.526901
 H   -2.616118   -1.395763   -2.981198
 H   -2.077386   -1.968926   -1.385572
 H   -0.964169    1.805914   -3.150254
 H   -2.127858    2.855570   -2.308665
 H   -2.692431    1.723576   -3.560671
 H   -0.876401    1.796617    0.324567
 H    0.137571    0.627366   -0.537747
 H   -0.963483    0.063001    0.735421
 H   -5.981801    3.214646   -1.450864
 H   -6.517577    2.618841   -3.030532
 H   -4.799656    3.001938   -2.764139
 H   -7.164171    0.819193    0.037087
 H   -6.806525   -0.847058   -0.467820
 H   -7.820841    0.196110   -1.493527
 H   -6.155772   -0.207796   -3.915717
 H   -5.086610   -1.341990   -3.055451
 H   -4.394351    0.037027   -3.943792
 H   -2.739212   -0.951866    1.798293
 H   -4.163719   -1.839328    2.383550
 H   -2.690130   -1.763883    3.379690
 H   -1.844858    1.657201    3.116711
 H   -1.863592    0.953884    4.742501
 H   -2.805083    2.425941    4.402637
 H   -4.114900   -0.721930    5.768674
 H   -5.687558   -0.803929    4.937900
 H   -5.174007    0.706412    5.728813
 H   -8.273821    0.789543    3.212994
 H   -8.596181    2.183867    4.270870
 H   -7.464197    0.912459    4.792314
 H   -6.465472    4.176163    1.257057
 H   -7.995606    4.134158    2.149081
 H   -7.646798    2.876432    0.945636
 H   -4.790762    4.191186    3.867026
 H   -5.181856    3.076821    5.198550
 H   -6.342132    4.338262    4.724870
 H   -4.073635   -0.238975    0.001224
 H   -5.668063    0.494112    1.801725
```

114

[**1**]$_2$^{conf}

Sn	4.898366	-8.382302	4.982292
Si	8.286969	-7.633908	4.967643
Si	7.416680	-10.696277	4.726967
Si	3.225014	-10.001360	7.390292
Si	4.676878	-7.274428	8.337842
C	9.999818	-8.258207	5.467876
C	8.299771	-7.170196	3.140321
C	8.032186	-6.017310	5.907250
C	8.352878	-10.710276	3.089577
C	5.882567	-11.761864	4.442384
C	8.459544	-11.556712	6.041689
C	4.600790	-11.069211	8.118939
C	1.789902	-9.918635	8.612426
C	2.500379	-10.851859	5.864281
C	3.620854	-7.285158	9.906009
C	4.789822	-5.471683	7.796402
C	6.381564	-7.897846	8.852324
C	6.989328	-8.957702	5.361376
C	3.889440	-8.286118	6.955834
H	10.754035	-7.475946	5.285378
H	10.309974	-9.156440	4.913362
H	10.025089	-8.501222	6.542565
H	9.046358	-6.378113	2.967881
H	7.319788	-6.769037	2.836985
H	8.537758	-8.012825	2.476906
H	7.971775	-6.145376	6.997975
H	7.124663	-5.493536	5.572284
H	8.890126	-5.357016	5.699045
H	7.762011	-10.254306	2.280582
H	8.551700	-11.756487	2.806026
H	9.320901	-10.190446	3.139340
H	6.192440	-12.768597	4.117987
H	5.232482	-11.350390	3.652053
H	5.280638	-11.884168	5.355527
H	8.728540	-12.576703	5.723394
H	7.904360	-11.637720	6.990258
H	9.390956	-11.007618	6.245623
H	4.299795	-12.127874	8.162966
H	4.844715	-10.749936	9.144593
H	5.525383	-11.005048	7.523477
H	1.015875	-9.219098	8.257158
H	2.094537	-9.601138	9.618889
H	1.322542	-10.913116	8.699129
H	3.213133	-11.001171	5.039495
H	1.657402	-10.263999	5.468125
H	2.112989	-11.845030	6.144697
H	3.670455	-8.253280	10.428086
H	2.562243	-7.067293	9.694429
H	3.991144	-6.515587	10.602966
H	5.282078	-4.856893	8.566796
H	3.779890	-5.059006	7.635717
H	5.354102	-5.353140	6.860188
H	6.718833	-7.342512	9.742421
H	7.140439	-7.760274	8.070687
H	6.358309	-8.966261	9.114941
H	7.010175	-9.069858	6.460325
H	2.979555	-7.716172	6.675555
Sn	4.418658	-5.964050	3.688864
C	2.522705	-5.149112	4.504262
C	4.216274	-5.985621	1.496485
Si	2.648512	-3.265101	4.586805
Si	0.886522	-5.894051	3.937242
H	2.622234	-5.464400	5.563508
Si	4.167604	-7.703253	0.697279
Si	5.355756	-4.740544	0.625696
H	3.206454	-5.563564	1.345444
C	1.478189	-2.530979	5.871954
C	2.269215	-2.511478	2.898087
C	4.371485	-2.726859	5.151106
C	0.576563	-5.713137	2.085087
C	0.836479	-7.716079	4.420694
C	-0.590807	-5.084720	4.795562
C	2.772397	-8.799517	1.339670

C	5.758169	-8.672084	0.991001
C	3.875398	-7.529246	-1.162339
C	5.966897	-3.381850	1.787712
C	6.909292	-5.509489	-0.117459
C	4.366494	-3.900908	-0.742952
H	1.609030	-3.028380	6.846729
H	1.709016	-1.462335	6.012195
H	0.419283	-2.612536	5.591614
H	2.805816	-3.032858	2.089487
H	1.192442	-2.572141	2.673807
H	2.556625	-1.448682	2.864399
H	4.417285	-1.626572	5.199351
H	4.576960	-3.114581	6.161349
H	5.192569	-3.058951	4.498329
H	-0.444906	-6.054766	1.852035
H	0.659081	-4.664371	1.762123
H	1.270181	-6.308013	1.476215
H	1.682845	-8.280780	4.003871
H	0.870373	-7.819094	5.518112
H	-0.092474	-8.193415	4.069979
H	-1.496741	-5.684262	4.608581
H	-0.450734	-5.016601	5.885805
H	-0.783032	-4.070076	4.413707
H	2.931374	-9.081111	2.391195
H	1.776009	-8.341751	1.253704
H	2.761203	-9.729057	0.747052
H	5.672640	-9.667053	0.524854
H	6.652265	-8.180781	0.583588
H	5.921241	-8.828279	2.069158
H	4.682194	-6.985897	-1.676515
H	3.795917	-8.525800	-1.625654
H	2.931901	-6.994452	-1.358330
H	6.584615	-2.666521	1.220738
H	5.140227	-2.812860	2.239417
H	6.595419	-3.782993	2.600479
H	7.541395	-5.973506	0.655000
H	6.692501	-6.269757	-0.882163
H	7.502158	-4.714346	-0.597775
H	4.983925	-3.158317	-1.273448
H	3.997173	-4.626448	-1.483063
H	3.492895	-3.373430	-0.326773

114

[**1**]$_2^{\text{X-ray}}$

Sn	-0.211110	0.612436	1.214681
Si	2.581625	2.640408	2.073493
Si	-0.383512	3.872350	1.987604
Si	-1.431159	-1.048967	3.820812
Si	1.500894	-1.956623	2.926936
C	2.746401	1.852124	3.781378
C	3.791771	1.835187	0.874976
C	3.172996	4.426646	2.266760
C	0.026108	5.568709	1.272413
C	-0.364056	3.935966	3.871348
C	-2.156178	3.522738	1.427815
C	-1.236992	-2.136645	5.350501
C	-2.324071	0.516369	4.384884
C	-2.534232	-1.954036	2.590886
C	0.724963	-3.544512	2.264075
C	2.213843	-2.286033	4.644701
C	2.990796	-1.571414	1.839504
C	0.831655	2.558154	1.363406
C	0.221338	-0.564137	3.035714
H	3.703979	2.162214	4.229722
H	2.742863	0.754454	3.742017
H	1.941314	2.171839	4.460753
H	4.774668	1.686601	1.350579
H	3.938863	2.485120	-0.001840
H	3.439947	0.858584	0.512747
H	4.230039	4.421809	2.579770
H	2.605544	4.985168	3.027205
H	3.109444	4.980624	1.317104
H	-0.759081	6.289659	1.552519
H	0.057674	5.523551	0.171746
H	0.988343	5.965071	1.623581

H	-1.131001	4.632351	4.245517
H	0.610624	4.269340	4.260116
H	-0.576619	2.943792	4.299768
H	-2.805706	4.357264	1.738832
H	-2.586932	2.600710	1.849479
H	-2.218096	3.443328	0.331402
H	-2.226416	-2.299715	5.807802
H	-0.592021	-1.662686	6.106983
H	-0.814378	-3.125004	5.113540
H	-3.306827	0.268111	4.816599
H	-2.498245	1.224181	3.558873
H	-1.743255	1.042453	5.159735
H	-3.465177	-2.283955	3.079059
H	-2.041210	-2.843346	2.169710
H	-2.813521	-1.298839	1.752416
H	1.497340	-4.322208	2.152025
H	0.271958	-3.384009	1.273999
H	-0.054986	-3.941575	2.931435
H	3.018827	-3.035888	4.578612
H	1.464193	-2.657852	5.356575
H	2.651451	-1.366777	5.066591
H	3.655330	-2.450962	1.824154
H	3.575079	-0.724697	2.228785
H	2.711981	-1.342755	0.800038
H	0.967048	2.794391	0.288912
H	0.661989	0.185120	3.717053
Sn	0.211031	-0.612241	-1.214406
C	-0.831684	-2.557928	-1.363088
C	-0.221378	0.563929	-3.035708
Si	-2.581617	-2.640072	-2.073221
Si	0.383496	-3.872063	-1.987363
H	-0.967127	-2.794224	-0.288616
Si	1.431126	1.048388	-3.821055
Si	-1.500815	1.956567	-2.927073
H	-0.662167	-0.185455	-3.716830
C	-2.746333	-1.851775	-3.781098
C	-3.791831	-1.834696	-0.874842
C	-3.173075	-4.426279	-2.266447
C	-0.025775	-5.568405	-1.271939
C	0.363705	-3.935933	-3.871095
C	2.156245	-3.522150	-1.428005
C	1.237034	2.136077	-5.350753
C	2.323589	-0.517180	-4.385166
C	2.534534	1.953316	-2.591335
C	-0.724767	3.544420	-2.264273
C	-2.213757	2.285883	-4.644865
C	-2.990750	1.571595	-1.839615
H	-3.704087	-2.161614	-4.229247
H	-2.742485	-0.754103	-3.741776
H	-1.941466	-2.171738	-4.460616
H	-4.774352	-1.685169	-1.350932
H	-3.939891	-2.484988	0.001543
H	-3.439564	-0.858520	-0.511888
H	-4.230179	-4.421397	-2.579257
H	-2.605786	-4.984800	-3.027017
H	-3.109358	-4.980276	-1.316814
H	0.759463	-6.289269	-1.552134
H	-0.057172	-5.523189	-0.171270
H	-0.988011	-5.964907	-1.622935
H	1.130865	-4.632101	-4.245232
H	-0.610904	-4.269766	-4.259656
H	0.575821	-2.943764	-4.299741
H	2.805834	-4.356535	-1.739274
H	2.586722	-2.600018	-1.849722
H	2.218460	-3.442808	-0.331602
H	2.226435	2.298893	-5.808193
H	0.591843	1.662281	-6.107146
H	0.814707	3.124544	-5.113736
H	3.306435	-0.269229	-4.816851
H	2.497504	-1.225071	-3.559170
H	1.742616	-1.043054	-5.160041
H	3.465256	2.283462	-3.079780
H	2.041507	2.842489	-2.169868
H	2.814212	1.298055	-1.753047
H	-1.497020	4.322311	-2.152712

H	-0.272232	3.384017	-1.273966
H	0.055555	3.941185	-2.931366
H	-3.018884	3.035586	-4.578801
H	-1.464153	2.657849	-5.356704
H	-2.651180	1.366550	-5.066778
H	-3.655284	2.451150	-1.824503
H	-3.575024	0.724787	-2.228700
H	-2.711988	1.343203	-0.800079

59

2

C	-2.529655	2.638092	-0.752383
Si	-3.241699	1.279386	0.346305
C	-2.657511	-0.443002	-0.168988
Si	-2.564742	-0.816868	-2.017528
C	-4.158666	-0.311215	-2.886224
C	-5.123647	1.397749	0.277583
C	-2.675173	1.622074	2.111946
C	-2.289066	-2.661044	-2.324324
C	-1.100582	0.077993	-2.799616
Sn	-3.772203	-1.967297	0.895671
C	-2.548002	-3.723882	1.234934
Si	-3.617523	-5.282458	1.229849
C	-2.669261	-6.805654	1.813700
C	-4.209439	-5.621506	-0.528154
C	-5.122373	-5.066929	2.347703
Si	-1.373119	-3.476251	2.692379
C	0.087964	-4.662548	2.572737
C	-0.646263	-1.732002	2.689329
C	-2.270920	-3.733654	4.329102
H	-2.832203	3.625040	-0.366567
H	-2.888136	2.563658	-1.790863
H	-1.428718	2.607975	-0.769762
H	-5.448340	2.419655	0.530979
H	-5.605471	0.714155	0.995130
H	-5.510766	1.161167	-0.725171
H	-3.101355	0.897256	2.823232
H	-2.989105	2.628450	2.432379
H	-1.577345	1.571215	2.192133
H	-1.359306	-3.010378	-1.846458
H	-2.196130	-2.850705	-3.405782
H	-3.116886	-3.285255	-1.952518
H	-1.204389	1.171601	-2.758344
H	-0.999122	-0.211733	-3.858059
H	-0.161527	-0.191115	-2.289382
H	-4.127446	-0.602814	-3.948036
H	-4.320558	0.777287	-2.844005
H	-5.032783	-0.801526	-2.428743
H	-1.411681	-0.949212	2.809837
H	-0.098002	-1.529834	1.754774
H	0.067738	-1.622668	3.521437
H	-1.601329	-3.510954	5.174969
H	-2.620787	-4.771448	4.445037
H	-3.146705	-3.070122	4.409991
H	0.812946	-4.455107	3.376428
H	0.612448	-4.539019	1.611362
H	-0.216176	-5.715552	2.657087
H	-4.823283	-6.535890	-0.561735
H	-3.357274	-5.763755	-1.212124
H	-4.822551	-4.793639	-0.917565
H	-5.709022	-5.999084	2.376945
H	-4.830926	-4.820743	3.380071
H	-5.792560	-4.269967	1.987153
H	-1.763950	-6.974223	1.209329
H	-3.306807	-7.699596	1.719595
H	-2.363573	-6.725449	2.868532
H	-1.625179	-0.546906	0.214387
H	-1.908109	-3.784509	0.334752
H	-4.309722	-1.364830	2.417240
H	-5.168828	-2.377412	-0.024787

59

TS_{HH}

C	0.033465	0.232312	0.046219
Si	-0.011782	0.057785	1.923747

C	1.722620	0.013448	2.681191
Si	3.051912	-1.011349	1.811268
C	2.417846	-2.744616	1.437364
C	-0.978287	-1.506958	2.338075
C	-0.932940	1.552355	2.615751
C	4.637620	-1.148035	2.822555
C	3.547546	-0.164264	0.196579
Sn	1.532203	-0.329281	4.834551
C	3.330904	0.302377	5.901132
Si	3.811583	-0.887899	7.292338
C	5.046753	-0.103963	8.486194
C	4.641080	-2.417843	6.571709
C	2.298776	-1.405506	8.291735
Si	3.155998	2.135395	6.326143
C	4.843844	2.915288	6.634323
C	2.392247	3.080278	4.874986
C	2.046318	2.365856	7.830697
H	-0.991818	0.356649	-0.338309
H	0.464268	-0.657404	-0.438865
H	0.619834	1.109648	-0.268943
H	-1.964159	-1.483232	1.846684
H	-1.151219	-1.604899	3.421573
H	-0.453338	-2.411509	1.995340
H	-1.054409	1.487451	3.709052
H	-1.939347	1.629825	2.174051
H	-0.394738	2.487144	2.389311
H	5.056144	-0.153858	3.049230
H	5.395825	-1.699672	2.243486
H	4.480996	-1.684134	3.770138
H	2.729598	-0.127321	-0.536498
H	4.390327	-0.702330	-0.267146
H	3.878875	0.870225	0.384060
H	3.220710	-3.362324	1.004748
H	1.585894	-2.725764	0.715813
H	2.066304	-3.243424	2.354541
H	1.356416	2.773010	4.653955
H	2.988435	2.948478	3.957117
H	2.367632	4.158506	5.100648
H	1.887884	3.436085	8.037558
H	2.483525	1.911584	8.733564
H	1.058405	1.905425	7.667582
H	4.738812	4.004293	6.766516
H	5.513530	2.747063	5.775485
H	5.337446	2.513693	7.530379
H	4.925198	-3.114851	7.376476
H	5.557905	-2.146988	6.023857
H	3.975905	-2.953573	5.877160
H	2.606650	-2.020354	9.152614
H	1.753339	-0.531245	8.679588
H	1.591225	-2.003055	7.695614
H	5.960638	0.222995	7.965610
H	5.342563	-0.845189	9.246264
H	4.626918	0.764892	9.016430
H	2.081307	1.060807	2.661248
H	4.127585	0.232236	5.139442
H	0.892986	-1.884826	5.266161
H	2.036703	-2.199439	4.724624

22

Et_3N

C	2.012936	2.426164	5.880093
C	1.844310	1.422202	7.012915
N	1.504931	0.086128	6.550075
C	1.950102	-0.959223	7.457564
C	3.440469	-1.253942	7.350094
C	0.106703	-0.046444	6.173380
H	-0.534789	-0.235179	7.064485
H	-0.232027	0.917114	5.763995
C	-0.132990	-1.120353	5.120318
H	1.095071	1.796948	7.747301
H	2.794929	1.363807	7.564018
H	1.683320	-0.718275	8.512061
H	1.399769	-1.881080	7.216557
H	3.725298	-2.070070	8.031233
H	3.694450	-1.548103	6.321255

H	4.055021	-0.378773	7.610107
H	2.299038	3.412872	6.274684
H	2.793617	2.084168	5.185012
H	1.085087	2.558908	5.303319
H	-1.197613	-1.163752	4.844927
H	0.456443	-0.902895	4.217636
H	0.155778	-2.120819	5.476525

23

Et_3NH^+

H	-0.074989	-1.003624	7.731146
C	0.373927	-0.644661	6.793897
N	1.659381	0.040703	7.180208
C	2.469450	-0.782220	8.148698
C	3.945220	-0.439624	8.120069
C	1.423417	1.440191	7.687200
C	1.140477	2.428539	6.573986
H	2.219384	0.126290	6.322724
H	-0.287311	0.126816	6.380326
C	0.581417	-1.759196	5.788481
H	0.594377	1.369514	8.405903
H	2.322848	1.734529	8.242024
H	2.020376	-0.619078	9.138927
H	2.314576	-1.834663	7.880607
H	4.471155	-1.090971	8.830872
H	4.382654	-0.620213	7.126246
H	4.152139	0.597978	8.416883
H	1.002588	3.424802	7.015050
H	1.984243	2.496789	5.870389
H	0.226146	2.193572	6.011611
H	-0.395488	-2.195423	5.540565
H	1.017822	-1.381449	4.851200
H	1.212043	-2.573264	6.172055

81

TS_1

C	-0.282166	-2.321649	2.718785
Si	0.048599	-0.608561	1.988425
C	0.012003	-0.796806	0.110311
C	1.620943	0.181665	2.652722
Sn	2.992461	-1.254564	3.764511
C	4.966611	-1.020016	2.689671
Si	6.405166	-0.841823	3.879393
C	6.238722	-2.009943	5.358348
C	-1.429578	0.464859	2.495240
Si	2.361539	1.569907	1.635654
C	3.655991	2.532950	2.622873
C	1.038451	2.843353	1.157271
C	3.168955	1.001029	0.026514
Si	5.077354	-2.428353	1.451874
C	3.399779	-2.739693	0.628331
C	5.581959	-4.042189	2.294104
C	6.288185	-2.043472	0.054593
C	8.085372	-1.188249	3.078130
C	6.520267	0.920066	4.558586
H	-1.261130	-2.692056	2.372736
H	-0.297962	-2.300646	3.820422
H	0.478975	-3.058934	2.417500
H	-1.349793	1.485147	2.090100
H	-1.492464	0.545781	3.593928
H	-2.379160	0.031740	2.141014
H	-0.932910	-1.273817	-0.196246
H	0.837435	-1.431231	-0.247413
H	0.078485	0.172955	-0.406729
H	2.460544	0.457253	-0.615844
H	4.034280	0.346132	0.202325
H	3.524129	1.879437	-0.536402
H	4.471523	1.887328	2.975593
H	3.215190	3.017667	3.508258
H	4.094237	3.327486	1.997028
H	0.522012	3.237963	2.047867
H	0.272091	2.414392	0.492403
H	1.495707	3.697313	0.631038
H	7.341601	0.991528	5.290373
H	5.587579	1.226215	5.056675

H	6.726461	1.644971	3.754791
H	6.106122	-3.055202	5.039593
H	5.374239	-1.743924	5.987557
H	7.140135	-1.959204	5.990468
H	8.895207	-1.019665	3.806788
H	8.260433	-0.517646	2.221463
H	8.173390	-2.224468	2.716219
H	7.317118	-1.917161	0.421958
H	6.000518	-1.115145	-0.465030
H	6.290421	-2.855630	-0.690673
H	3.027110	-1.827125	0.140082
H	2.632500	-3.075974	1.346177
H	3.487241	-3.523292	-0.141490
H	5.577252	-4.873186	1.570656
H	4.876686	-4.296233	3.102209
H	6.590134	-3.986395	2.733376
H	1.235226	0.705384	3.550966
H	4.927614	-0.087738	2.100702
H	3.449231	0.330063	4.916907
H	2.782911	0.562782	5.615058
N	2.062933	0.946959	6.657451
C	0.755845	0.268439	6.600298
C	2.940057	0.532427	7.771928
C	2.021446	2.419966	6.565107
C	-0.165392	0.548531	7.778122
H	0.277710	0.561010	5.655482
H	0.954568	-0.807954	6.504152
H	1.763182	2.841149	7.553029
H	3.054875	2.735522	6.346325
C	1.088814	2.967698	5.499379
H	3.932979	0.951242	7.539769
H	2.602004	1.014046	8.706482
C	3.055807	-0.971631	7.952110
H	3.873302	-1.186729	8.653806
H	2.139339	-1.416385	8.365315
H	3.283799	-1.475182	7.000827
H	1.258711	4.048493	5.398368
H	1.280780	2.509303	4.519899
H	0.028463	2.822064	5.749335
H	-0.400814	1.619347	7.871047
H	0.270600	0.211017	8.730103
H	-1.113841	0.010990	7.638832

81

int$_1$

C	-0.287064	-2.173313	2.987771
Si	0.057543	-0.529477	2.115779
C	-1.401260	0.597886	2.571116
C	-0.028960	-0.869948	0.260968
C	1.653848	0.285012	2.671081
Si	2.369558	1.594079	1.541979
C	3.127732	0.920774	-0.050850
Sn	3.078624	-1.111300	3.806234
C	5.017792	-0.956246	2.648616
Si	5.094306	-2.431009	1.492934
C	6.301115	-2.146560	0.065795
Si	6.472323	-0.716060	3.803838
C	6.618420	1.086239	4.359767
C	6.314120	-1.776120	5.365586
C	8.141753	-1.141402	3.014874
C	3.693882	2.609967	2.431824
C	1.044518	2.848024	1.013135
C	3.411181	-2.777673	0.695443
C	5.590232	-3.998555	2.425230
N	2.017117	0.834362	6.701170
C	1.991184	2.322186	6.753163
C	1.257292	2.972696	5.596731
C	0.696236	0.188405	6.472000
C	-0.311183	0.413851	7.582895
C	2.792510	0.293568	7.855099
C	2.861213	-1.221107	7.905800
H	-1.278500	-2.554179	2.691727
H	-0.278207	-2.065777	4.084657
H	0.461410	-2.940501	2.734824
H	-1.325858	1.581142	2.082417

H	-1.436466	0.770920	3.660950
H	-2.363391	0.145867	2.279521
H	-0.979856	-1.369026	0.013933
H	0.790244	-1.531972	-0.059608
H	0.027768	0.054540	-0.334393
H	2.399462	0.337880	-0.634111
H	3.997432	0.276455	0.141154
H	3.465211	1.760251	-0.680247
H	4.488254	1.974083	2.845515
H	3.268739	3.190595	3.266605
H	4.154682	3.329934	1.735942
H	0.550947	3.300796	1.889167
H	0.260460	2.385378	0.392748
H	1.495673	3.664174	0.425017
H	7.456408	1.201759	5.066764
H	5.696321	1.426066	4.855764
H	6.809057	1.753776	3.504053
H	6.167072	-2.839830	5.122863
H	5.457701	-1.453576	5.980059
H	7.222961	-1.689960	5.983280
H	8.961104	-0.940783	3.724712
H	8.319890	-0.529644	2.115765
H	8.208926	-2.199728	2.718055
H	7.334077	-2.010976	0.418722
H	6.020792	-1.246477	-0.505382
H	6.288888	-3.000651	-0.631039
H	3.049290	-1.899147	0.140978
H	2.644455	-3.046134	1.441160
H	3.487682	-3.617158	-0.014511
H	5.569323	-4.872451	1.754471
H	4.888361	-4.191670	3.252914
H	6.603025	-3.927548	2.851810
H	1.315008	0.880715	3.542576
H	4.970209	-0.051848	2.017822
H	3.542484	0.383253	4.905378
H	2.650140	0.589912	5.823308
H	0.322226	0.564893	5.511171
H	0.895545	-0.879196	6.311653
H	1.563941	2.624342	7.721941
H	3.046667	2.632588	6.748142
H	3.804369	0.710870	7.740902
H	2.365469	0.710505	8.780468
H	3.612265	-1.513452	8.652107
H	1.904817	-1.674848	8.201352
H	3.161991	-1.642214	6.934079
H	1.424730	4.057459	5.639158
H	1.638832	2.613329	4.631291
H	0.172124	2.803371	5.632302
H	-0.552704	1.478660	7.716333
H	0.036363	0.012754	8.546302
H	-1.244520	-0.105657	7.326410

81

TS_2

C	0.008167	-0.036085	0.006259
Si	0.019549	-0.008267	1.895113
C	-1.738287	0.410196	2.458052
C	1.215511	1.217668	2.671234
Sn	3.045218	0.426782	3.699968
C	4.806803	1.114605	2.500907
Si	6.212955	1.715539	3.592488
C	5.785746	3.361917	4.406810
C	0.313094	-1.796462	2.448383
Si	1.427237	2.844232	1.752959
C	2.344870	2.690438	0.107080
C	2.336909	4.093731	2.833276
C	-0.259642	3.611904	1.356195
Si	5.263961	-0.118606	1.167509
C	6.189466	0.692126	-0.266552
C	3.728552	-0.899932	0.389103
C	6.345313	-1.503753	1.871055
C	6.609039	0.474323	4.963599
C	7.818736	2.007447	2.631787
H	-0.732243	-0.775486	-0.339928
H	0.984922	-0.321336	-0.411421

H	-0.273924	0.938669	-0.420283
H	-0.436196	-2.464273	1.993398
H	0.217603	-1.876445	3.542400
H	1.312882	-2.158998	2.164365
H	-2.470157	-0.299766	2.039432
H	-2.031751	1.423218	2.143264
H	-1.818556	0.367755	3.556823
H	-0.129609	4.606017	0.898009
H	-0.860881	3.741208	2.270826
H	-0.845173	2.997533	0.654179
H	2.409540	3.683067	-0.367704
H	1.821948	2.021346	-0.592055
H	3.370440	2.313876	0.227817
H	2.405168	5.069942	2.326480
H	3.356296	3.764864	3.079974
H	1.804038	4.242274	3.786269
H	8.584186	2.426584	3.305348
H	7.666757	2.726668	1.811074
H	8.228977	1.082439	2.197454
H	6.595758	3.676484	5.084878
H	4.854584	3.293221	4.989166
H	5.657248	4.154401	3.651970
H	7.494941	0.798446	5.533073
H	6.813370	-0.529977	4.561243
H	5.768887	0.401666	5.669850
H	6.468120	-2.319661	1.140844
H	5.900656	-1.929260	2.783643
H	7.348953	-1.134008	2.133309
H	6.386613	-0.043808	-1.063238
H	7.152946	1.120811	0.044408
H	5.586940	1.503454	-0.706373
H	4.006481	-1.617217	-0.399756
H	3.109952	-0.114555	-0.070545
H	3.095702	-1.428348	1.118532
H	0.669599	1.537979	3.583451
H	4.449488	2.019817	1.978338
H	3.106526	1.624147	4.994256
H	2.949526	-1.473536	4.565491
N	2.914904	-2.527966	5.308563
C	1.742439	-2.305472	6.172801
C	2.746087	-3.704672	4.431762
C	4.228054	-2.509631	5.989038
C	1.467366	-3.391372	7.201427
H	1.874024	-1.327687	6.656492
H	0.881474	-2.189896	5.497983
H	4.453768	-3.520643	6.370408
H	4.971240	-2.278726	5.209181
C	4.352721	-1.477420	7.097080
H	2.792732	-4.621095	5.045894
H	1.731396	-3.639891	4.015089
C	3.752829	-3.782044	3.297467
H	3.481709	-4.616404	2.635881
H	3.751513	-2.860698	2.700220
H	4.777319	-3.962402	3.652055
H	5.404122	-1.422098	7.410443
H	4.056742	-0.477650	6.747739
H	3.752939	-1.730450	7.982493
H	0.606537	-3.098328	7.818904
H	1.221418	-4.354146	6.731080
H	2.321776	-3.548322	7.876320

81

int$_2$

C	0.009258	-0.060759	0.039539
Si	0.001463	0.035425	1.926626
C	0.268652	-1.742046	2.536267
C	-1.760576	0.480874	2.457676
C	1.207003	1.265841	2.669321
Si	1.429265	2.865411	1.712446
C	-0.250763	3.634373	1.281523
Sn	3.033503	0.455433	3.725723
C	4.795965	1.160674	2.506775
Si	5.259547	-0.108171	1.215664
C	6.300481	-1.492377	1.986309
Si	6.188031	1.783621	3.598412

C	7.823334	2.013670	2.668366
C	5.773466	3.465623	4.343884
C	6.539211	0.595474	5.030721
C	2.350589	2.670003	0.071841
C	2.336056	4.141819	2.762938
C	6.235448	0.635049	-0.222792
C	3.733773	-0.892297	0.416541
N	2.921453	-2.594716	5.295324
C	4.266831	-2.609832	5.932564
C	4.439425	-1.599748	7.052314
C	1.778455	-2.406015	6.221414
C	1.566588	-3.528202	7.221799
C	2.710523	-3.744616	4.377232
C	3.687427	-3.795091	3.217499
H	-0.725982	-0.812037	-0.291879
H	0.992779	-0.357300	-0.353948
H	-0.267040	0.899243	-0.422923
H	-0.496466	-2.412184	2.111757
H	0.187757	-1.782720	3.634225
H	1.260510	-2.123618	2.248620
H	-2.493044	-0.238290	2.055875
H	-2.044601	1.484689	2.106868
H	-1.851219	0.476173	3.556524
H	-0.111159	4.617090	0.801807
H	-0.860417	3.787668	2.186921
H	-0.832747	3.006996	0.587820
H	2.417034	3.650601	-0.427238
H	1.826971	1.984669	-0.611054
H	3.375255	2.294582	0.203198
H	2.413440	5.103270	2.229780
H	3.350532	3.812678	3.027527
H	1.795967	4.318625	3.707033
H	8.580573	2.443347	3.344707
H	7.703381	2.705545	1.819263
H	8.226927	1.067102	2.275985
H	6.577646	3.795256	5.021833
H	4.833139	3.427043	4.914293
H	5.664579	4.231042	3.558686
H	7.436640	0.912843	5.585894
H	6.703601	-0.435406	4.679658
H	5.694210	0.594408	5.735452
H	6.419101	-2.339039	1.291129
H	5.828719	-1.869659	2.906729
H	7.306526	-1.133383	2.254383
H	6.424843	-0.125877	-0.997706
H	7.205199	1.044322	0.095029
H	5.664028	1.452161	-0.692665
H	4.021669	-1.679897	-0.298530
H	3.173618	-0.123266	-0.136651
H	3.040313	-1.332824	1.149514
H	0.670111	1.612339	3.577468
H	4.436975	2.053347	1.964989
H	3.096048	1.720031	4.965773
H	2.928154	-1.645054	4.664415
H	1.931808	-1.441993	6.724065
H	0.892640	-2.277413	5.583496
H	4.476574	-3.634325	6.279148
H	4.976983	-2.374292	5.126027
H	2.759849	-4.671044	4.971069
H	1.685899	-3.644564	3.994416
H	3.376644	-4.594493	2.530928
H	3.691281	-2.849433	2.659157
H	4.715602	-4.014303	3.536713
H	5.503735	-1.551107	7.318894
H	4.133445	-0.594970	6.728183
H	3.877348	-1.868201	7.957525
H	0.733260	-3.261186	7.886407
H	1.304471	-4.477560	6.733858
H	2.451555	-3.696358	7.852668

81

TS_3

C	0.008167	-0.036085	0.006259
Si	0.019549	-0.008267	1.895113
C	-1.738287	0.410196	2.458052

C	1.215511	1.217668	2.671234
Sn	3.045218	0.426782	3.699968
C	4.806803	1.114605	2.500907
Si	6.212955	1.715539	3.592488
C	5.785746	3.361917	4.406810
C	0.313094	-1.796462	2.448383
Si	1.427237	2.844232	1.752959
C	2.344870	2.690438	0.107080
C	2.336909	4.093731	2.833276
C	-0.259642	3.611904	1.356195
Si	5.263961	-0.118606	1.167509
C	6.189466	0.692126	-0.266552
C	3.728552	-0.899932	0.389103
C	6.345313	-1.503753	1.871055
C	6.609039	0.474323	4.963599
C	7.818736	2.007447	2.631787
H	-0.732243	-0.775486	-0.339928
H	0.984922	-0.321336	-0.411421
H	-0.273924	0.938669	-0.420283
H	-0.436196	-2.464273	1.993398
H	0.217603	-1.876445	3.542400
H	1.312882	-2.158998	2.164365
H	-2.470157	-0.299766	2.039432
H	-2.031751	1.423218	2.143264
H	-1.818556	0.367755	3.556823
H	-0.129609	4.606017	0.898009
H	-0.860881	3.741208	2.270826
H	-0.845173	2.997533	0.654179
H	2.409540	3.683067	-0.367704
H	1.821948	2.021346	-0.592055
H	3.370440	2.313876	0.227817
H	2.405168	5.069942	2.326480
H	3.356296	3.764864	3.079974
H	1.804038	4.242274	3.786269
H	8.584186	2.426584	3.305348
H	7.666757	2.726668	1.811074
H	8.228977	1.082439	2.197454
H	6.595758	3.676484	5.084878
H	4.854584	3.293221	4.989166
H	5.657248	4.154401	3.651970
H	7.494941	0.798446	5.533073
H	6.813370	-0.529977	4.561243
H	5.768887	0.401666	5.669850
H	6.468120	-2.319661	1.140844
H	5.900656	-1.929260	2.783643
H	7.348953	-1.134008	2.133309
H	6.386613	-0.043808	-1.063238
H	7.152946	1.120811	0.044408
H	5.586940	1.503454	-0.706373
H	4.006481	-1.617217	-0.399756
H	3.109952	-0.114555	-0.070545
H	3.095702	-1.428348	1.118532
H	0.669599	1.537979	3.583451
H	4.449488	2.019817	1.978338
H	3.106526	1.624147	4.994256
H	2.949526	-1.473536	4.565491
N	2.914904	-2.527966	5.308563
C	1.742439	-2.305472	6.172801
C	2.746087	-3.704672	4.431762
C	4.228054	-2.509631	5.989038
C	1.467366	-3.391372	7.201427
H	1.874024	-1.327687	6.656492
H	0.881474	-2.189896	5.497983
H	4.453768	-3.520643	6.370408
H	4.971240	-2.278726	5.209181
C	4.352721	-1.477420	7.097080
H	2.792732	-4.621095	5.045894
H	1.731396	-3.639891	4.015089
C	3.752829	-3.782044	3.297467
H	3.481709	-4.616404	2.635881
H	3.751513	-2.860698	2.700220
H	4.777319	-3.962402	3.652055
H	5.404122	-1.422098	7.410443
H	4.056742	-0.477650	6.747739
H	3.752939	-1.730450	7.982493

H	0.606537	-3.098328	7.818904
H	1.221418	-4.354146	6.731080
H	2.321776	-3.548322	7.876320

19
DMAP

C	1.668179	0.400849	6.044396
N	2.012000	-0.819949	5.637034
C	2.415182	-1.659030	6.589886
C	2.495643	-1.345867	7.941176
C	2.131360	-0.051672	8.374838
C	1.704208	0.836923	7.363124
N	2.188691	0.318170	9.692478
C	2.635496	-0.629212	10.687086
C	1.804911	1.655105	10.081962
H	2.700005	-2.665864	6.262459
H	2.839143	-2.107832	8.639748
H	1.401343	1.858475	7.589601
H	1.335218	1.097373	5.265914
H	1.913231	1.763983	11.167634
H	2.436896	2.423483	9.602822
H	0.753442	1.874606	9.826083
H	2.616307	-0.156105	11.676057
H	1.986680	-1.521718	10.729342
H	3.668445	-0.970915	10.498435

20
DMAPH^+

C	1.698688	0.852162	7.355578
C	1.653952	0.447867	6.052553
N	2.015951	-0.807510	5.698219
C	2.433956	-1.701921	6.624477
C	2.503680	-1.366419	7.945769
C	2.132757	-0.051864	8.379057
N	2.188394	0.309559	9.662123
C	1.802047	1.657383	10.067852
C	2.637009	-0.637553	10.678123
H	2.708103	-2.692364	6.259845
H	2.846151	-2.122882	8.648037
H	1.397159	1.871466	7.585319
H	1.328957	1.109020	5.248593
H	1.913866	1.751599	11.152168
H	2.443698	2.415014	9.592782
H	0.750498	1.862298	9.815717
H	2.612521	-0.151084	11.657856
H	1.978119	-1.518198	10.719373
H	3.670131	-0.965553	10.487284
H	1.973228	-1.082724	4.721511

76
1-DMAP

C	0.505133	1.353156	0.925268
Si	-0.307289	1.856914	2.559646
C	-0.006940	0.551537	3.887622
Sn	2.013528	-0.432855	3.557015
C	3.387148	1.385001	3.654825
Si	4.968918	0.907127	2.757559
C	5.780358	-0.621844	3.526296
C	-2.133263	2.184940	2.174534
C	0.359729	3.552861	3.059998
Si	-1.308663	-0.806287	3.953080
C	-1.569237	-1.545700	2.233612
C	-0.837132	-2.266740	5.069814
C	-2.959829	-0.190980	4.641084
Si	3.602815	2.408879	5.211742
C	4.151448	4.169887	4.777807
C	4.902404	1.670379	6.372958
C	2.036749	2.658197	6.243984
C	4.570906	0.513769	0.952036
C	6.295496	2.256568	2.721668
N	2.299763	-0.964738	5.881409
H	0.262390	2.084216	0.137291
H	0.149899	0.366764	0.587216
H	1.604498	1.304802	0.994121
H	-2.201119	2.962294	1.395642

H	-2.665594	2.559201	3.063511
H	-2.671459	1.298901	1.806075
H	1.438328	3.556357	3.267630
H	-0.157832	3.927440	3.958040
H	0.171572	4.273239	2.247305
H	0.153853	-2.686219	4.834473
H	-1.578457	-3.070076	4.926162
H	-0.849807	-2.002629	6.138750
H	-2.816472	0.274606	5.630238
H	-3.658835	-1.033945	4.768207
H	-3.441211	0.551724	3.989554
H	-2.309895	-2.360345	2.274334
H	-0.626344	-1.969301	1.850523
H	-1.925884	-0.806039	1.500992
H	4.149095	1.396936	0.444483
H	3.842095	-0.307614	0.860530
H	5.480558	0.217644	0.404875
H	7.153731	1.913917	2.120533
H	6.671070	2.505834	3.726397
H	5.914647	3.183197	2.263719
H	5.953733	-0.488202	4.605907
H	6.755269	-0.816923	3.051280
H	5.157172	-1.519135	3.379546
H	4.620220	0.661356	6.711665
H	5.010248	2.305028	7.267524
H	5.891695	1.601303	5.893667
H	1.747839	1.757267	6.799847
H	1.183116	2.971483	5.626767
H	2.224246	3.453442	6.984071
H	4.239730	4.777901	5.693323
H	3.407955	4.659717	4.127793
H	5.119377	4.199807	4.258420
H	0.008625	1.090496	4.853562
H	2.869484	2.064370	2.949348
C	1.451320	-0.675296	6.873563
C	1.653827	-1.043445	8.188933
C	2.816958	-1.769186	8.543594
C	3.696434	-2.084604	7.478450
C	3.393221	-1.671103	6.197202
H	0.557411	-0.114056	6.594811
H	0.906839	-0.758058	8.926863
N	3.072558	-2.142482	9.824817
H	4.614340	-2.646631	7.638160
H	4.070060	-1.909803	5.372453
C	4.281477	-2.876032	10.137507
C	2.141781	-1.795810	10.878667
H	5.186338	-2.305042	9.869725
H	4.317105	-3.074722	11.214521
H	4.318611	-3.846392	9.614388
H	2.517891	-2.176270	11.834956
H	2.020618	-0.703615	10.974091
H	1.146529	-2.239336	10.707517

78

TS_1^{DMAP}

C	-4.351721	-1.483220	-0.854014
C	-2.996155	-1.337154	-0.648255
N	-2.467024	-1.175343	0.568596
C	-3.273340	-1.153086	1.633428
C	-4.641300	-1.291230	1.533487
C	-5.236791	-1.459503	0.255070
N	-6.578614	-1.588050	0.102809
C	-7.146673	-1.744228	-1.221736
C	-7.448390	-1.550354	1.261517
C	0.907660	4.081684	2.145101
Si	0.327300	2.344526	1.660669
C	0.678179	1.986476	-0.142190
Sn	0.783844	-0.182099	-0.822175
C	2.715117	-0.735916	0.208518
Si	4.131636	-0.365226	-0.964107
C	3.899061	1.294800	-1.847664
C	-1.528797	2.234953	2.025438
C	1.207688	1.190510	2.868120
Si	-0.375174	2.904760	-1.392360
C	0.478273	2.899045	-3.078975

C	-0.703451	4.719265	-0.962178
C	-2.070679	2.081373	-1.603827
Si	2.651438	-2.475379	0.909096
C	1.794650	-3.667410	-0.285074
C	1.701834	-2.531801	2.543525
C	4.356793	-3.207677	1.289102
C	4.267938	-1.689035	-2.305972
C	5.785186	-0.226556	-0.058538
H	5.724984	0.531625	0.739302
H	6.581436	0.083572	-0.755077
H	6.090753	-1.176078	0.404000
H	3.319933	-1.781417	-2.860740
H	5.058219	-1.431122	-3.029107
H	4.508020	-2.678568	-1.886282
H	4.751318	1.483071	-2.520803
H	3.853086	2.128460	-1.128280
H	2.983952	1.331846	-2.461033
H	2.172956	-1.885610	3.301150
H	1.689702	-3.561079	2.938039
H	0.658956	-2.202744	2.418294
H	4.978227	-3.315392	0.386412
H	4.244149	-4.208633	1.737155
H	4.910052	-2.579718	2.005772
H	2.263427	-3.650320	-1.281204
H	0.730294	-3.412603	-0.416889
H	1.847271	-4.699533	0.098016
H	2.302185	1.291544	2.790679
H	0.954090	0.135191	2.697456
H	0.925430	1.450819	3.901630
H	0.759895	4.250808	3.224607
H	0.374758	4.875476	1.602371
H	1.983851	4.198125	1.935748
H	-1.939074	1.271253	1.688552
H	-1.720721	2.331418	3.106234
H	-2.089859	3.032557	1.513105
H	0.236894	5.272587	-0.809679
H	-1.308653	4.826468	-0.048143
H	-1.251811	5.207404	-1.784636
H	-2.727785	2.705150	-2.231427
H	-2.574158	1.923472	-0.637239
H	-1.967590	1.102483	-2.098536
H	-0.122345	3.440579	-3.827795
H	0.631860	1.872565	-3.449787
H	1.464889	3.386758	-3.020946
H	2.821731	-0.037751	1.057395
H	1.720484	2.321254	-0.309711
H	-0.210635	-0.744347	0.807766
H	-2.787720	-1.009684	2.602636
H	-5.237461	-1.260280	2.443012
H	-4.712464	-1.606220	-1.872874
H	-2.284690	-1.341125	-1.479423
H	-6.774750	-2.654837	-1.719926
H	-8.236055	-1.827591	-1.141124
H	-6.920743	-0.879072	-1.866982
H	-8.488127	-1.670821	0.938040
H	-7.371455	-0.590614	1.799281
H	-7.219946	-2.364140	1.969655
H	-1.176364	-0.940038	0.683922

78

int$_1$^{DMAP}

C	-5.221961	-1.380741	-0.030391
C	-4.197956	-1.662325	-0.976661
C	-2.878804	-1.546020	-0.614078
N	-2.526615	-1.183455	0.629210
C	-3.455731	-0.915298	1.557120
C	-4.796331	-1.004164	1.275588
N	-6.529994	-1.462001	-0.353723
C	-7.547323	-1.150369	0.634201
C	-6.925537	-1.832357	-1.701222
Sn	0.698715	-0.261750	-0.557175
C	2.759700	-0.745926	0.243351
Si	2.790114	-2.453543	1.011351
C	4.531346	-3.176129	1.213704
C	0.634664	1.953989	0.011361

Si	-0.572014	2.790660	-1.145766
C	-2.261431	1.917677	-1.154576
Si	0.487540	2.369528	1.825048
C	1.558112	1.305503	2.957062
C	1.015526	4.153681	2.191334
C	-1.304829	2.172104	2.415706
Si	4.021353	-0.402201	-1.095726
C	5.777649	-0.253521	-0.405580
C	3.692251	1.243259	-1.975336
C	3.997219	-1.756638	-2.414946
C	0.089736	2.754634	-2.915195
C	-0.929703	4.607568	-0.741029
C	1.810770	-3.700438	-0.025381
C	2.037464	-2.443779	2.745772
H	5.817780	0.516503	0.382314
H	6.480923	0.045107	-1.200499
H	6.139375	-1.196320	0.029641
H	2.986012	-1.863215	-2.840346
H	4.688631	-1.515659	-3.238370
H	4.293864	-2.734464	-2.003292
H	4.457745	1.417727	-2.749049
H	3.735850	2.087006	-1.267374
H	2.707845	1.271018	-2.469404
H	2.601598	-1.779431	3.419737
H	2.054786	-3.457797	3.177997
H	0.993363	-2.095622	2.722440
H	5.038247	-3.320849	0.246568
H	4.477247	-4.158101	1.712194
H	5.166230	-2.520465	1.831073
H	2.162451	-3.721947	-1.068608
H	0.736964	-3.451803	-0.043312
H	1.915018	-4.715514	0.391563
H	2.629482	1.443634	2.739783
H	1.325303	0.237029	2.849590
H	1.393744	1.593695	4.008502
H	1.000749	4.346099	3.277023
H	0.368855	4.899113	1.706527
H	2.045778	4.326408	1.838511
H	-1.648291	1.148732	2.201032
H	-1.383564	2.339736	3.501789
H	-1.986915	2.875240	1.911817
H	-0.001636	5.199789	-0.700129
H	-1.443073	4.722361	0.227032
H	-1.577009	5.048430	-1.517189
H	-3.006878	2.521592	-1.697342
H	-2.639694	1.747189	-0.133811
H	-2.183637	0.943415	-1.662680
H	-0.610257	3.241257	-3.613899
H	0.249867	1.719483	-3.256948
H	1.054745	3.282937	-2.980943
H	2.951010	-0.020296	1.053398
H	1.639739	2.298465	-0.299918
H	-0.151923	-0.768212	1.056802
H	-3.085211	-0.615318	2.538672
H	-5.507300	-0.771027	2.064614
H	-4.426455	-1.953195	-1.999085
H	-2.047553	-1.713365	-1.305060
H	-6.560895	-2.837429	-1.967571
H	-8.018659	-1.843285	-1.766441
H	-6.548085	-1.113185	-2.446047
H	-8.537845	-1.273123	0.183559
H	-7.463239	-0.110226	0.988850
H	-7.486716	-1.824145	1.504102
H	-1.470982	-1.026698	0.840640

27

DBU

N	-0.284584	0.651916	-0.373045
N	-1.452504	-1.395682	-0.144069
C	0.931649	1.351356	-0.745210
C	2.031062	1.324074	0.319998
C	-0.377422	-0.726058	-0.343737
C	2.806286	0.008956	0.370134
C	-1.422951	1.476888	0.000389
C	0.907480	-1.505364	-0.513515

C	1.954457	-1.242906	0.579422
C	-2.451274	0.664713	0.769416
C	-2.680738	-0.659616	0.048175
H	0.646933	2.393656	-0.951436
H	1.326016	0.959528	-1.698848
H	1.568845	1.535668	1.299675
H	2.738312	2.146153	0.121082
H	3.566818	0.067660	1.165582
H	3.365064	-0.103208	-0.576944
H	-1.886602	1.920105	-0.902307
H	-1.064707	2.317921	0.618718
H	0.597081	-2.556732	-0.495813
H	1.348316	-1.315614	-1.507880
H	1.444901	-1.198321	1.557317
H	2.629028	-2.112309	0.627452
H	-3.382847	1.240811	0.873257
H	-2.075511	0.457980	1.785091
H	-3.387606	-1.287741	0.613080
H	-3.159501	-0.467834	-0.931984

28
DBUH^+

C	-2.739034	-0.663292	0.121845
N	-1.457862	-1.318911	-0.114044
C	-0.320448	-0.676385	-0.348633
N	-0.275559	0.644297	-0.378417
C	-1.461870	1.461633	-0.081396
C	-2.475100	0.694064	0.749102
C	0.961261	1.373459	-0.712088
C	2.032967	1.298037	0.372381
C	2.819106	-0.011371	0.379992
C	1.978232	-1.273968	0.557574
C	0.922190	-1.497245	-0.540832
H	0.658804	2.414147	-0.881798
H	1.347022	1.006139	-1.675888
H	1.561165	1.479256	1.352894
H	2.732753	2.131157	0.208967
H	3.573247	0.026576	1.179684
H	3.383361	-0.092373	-0.565139
H	-1.899142	1.805248	-1.033357
H	-1.119706	2.351271	0.464637
H	0.626675	-2.554800	-0.568880
H	1.346950	-1.272127	-1.532719
H	1.482173	-1.275550	1.542589
H	2.645876	-2.147371	0.551240
H	-3.406757	1.270513	0.816740
H	-2.096022	0.560863	1.774146
H	-3.335129	-1.302367	0.785691
H	-3.285049	-0.565998	-0.830382
H	-1.420975	-2.330909	-0.100637

84
1-DBU

C	2.419249	1.585394	7.394551
C	3.628889	1.370232	6.498060
N	3.748071	-0.031499	6.133364
C	3.585630	-0.892652	7.099466
N	3.204656	-0.538205	8.356205
C	2.628525	0.771987	8.656311
C	3.810408	-2.360252	6.836901
C	2.553920	-3.227850	6.999193
C	2.241172	-3.641069	8.435808
C	2.006503	-2.481412	9.401243
C	3.169873	-1.489767	9.461562
C	6.954892	-1.224519	5.001678
Si	6.770472	0.215873	3.790344
C	8.355716	0.280174	2.751659
C	5.203220	0.082394	2.768163
Si	5.371869	-1.002972	1.232076
C	6.241979	-2.631475	1.638794
Sn	3.363019	-0.731597	3.821229
C	1.841284	0.975732	3.622835
Si	2.239114	2.472151	2.564406
C	0.794548	3.690955	2.416954
Si	0.152210	0.174602	3.439296

C	-0.058715	-1.214350	4.715425
C	-0.078666	-0.546584	1.712035
C	-1.281971	1.357845	3.797607
C	6.812357	1.825939	4.779146
C	2.708014	2.059425	0.782725
C	3.636895	3.473799	3.354471
C	6.298940	-0.103093	-0.145872
C	3.702410	-1.514216	0.487075
H	-1.033260	-1.090364	1.630174
H	0.733666	-1.247495	1.461062
H	-0.080658	0.248822	0.949337
H	-1.105351	-1.558542	4.739923
H	0.198130	-0.854504	5.726895
H	0.581202	-2.082209	4.494938
H	-2.223007	0.788571	3.874331
H	-1.415295	2.121240	3.018708
H	-1.131284	1.882143	4.755832
H	1.153765	4.631820	1.967826
H	0.350420	3.932040	3.395635
H	-0.006830	3.302346	1.769146
H	2.806029	2.992683	0.204877
H	1.929654	1.449443	0.297768
H	3.659546	1.516537	0.703185
H	3.949645	4.292171	2.685989
H	4.523796	2.865485	3.580251
H	3.294792	3.935610	4.295959
H	9.225606	0.402191	3.418087
H	8.340780	1.141901	2.065048
H	8.523137	-0.625720	2.149844
H	7.711135	1.854777	5.416631
H	5.934606	1.947692	5.426319
H	6.855760	2.696439	4.104994
H	7.957433	-1.221490	5.458909
H	6.811331	-2.197305	4.506184
H	6.213062	-1.138185	5.809648
H	6.286032	-3.272016	0.743326
H	5.677472	-3.178482	2.411851
H	7.270344	-2.495032	2.005408
H	6.350715	-0.732880	-1.049016
H	7.325914	0.161497	0.143607
H	5.776513	0.827969	-0.419575
H	3.879977	-2.005910	-0.483646
H	3.032789	-0.660801	0.305784
H	3.168968	-2.238196	1.124174
H	1.803347	1.386980	4.649828
H	4.987692	1.103652	2.409905
H	3.105239	-0.891191	10.381306
H	4.128688	-2.028651	9.522058
H	1.090658	-1.930455	9.125752
H	1.836477	-2.883727	10.412682
H	1.358534	-4.299686	8.439674
H	3.079172	-4.251209	8.818460
H	3.299217	1.306487	9.351978
H	1.672474	0.617321	9.183989
H	4.185290	-2.447720	5.811248
H	4.619574	-2.722615	7.493233
H	1.694997	-2.699921	6.554133
H	2.692463	-4.135975	6.393286
H	2.284995	2.646299	7.648319
H	1.513190	1.252682	6.863189
H	3.573500	1.976774	5.587981
H	4.541482	1.684961	7.036844

86

TS_1^{DBU}

C	4.914431	-3.595770	2.374609
Si	5.782716	-2.151390	3.229298
C	5.810644	-2.538428	5.082582
C	4.878616	-0.542495	2.873371
Sn	2.788077	-0.710312	3.749511
C	1.792305	0.691791	2.280670
Si	1.179525	-0.331404	0.828104
C	-0.398009	-1.267686	1.280846
C	7.579306	-2.182788	2.635041
Si	5.866892	1.027656	3.132070

C	4.847428	2.616365	3.048182
C	7.185536	1.236911	1.790006
C	6.733338	1.010892	4.814945
Si	0.501299	1.793940	3.086662
C	1.301353	3.288123	3.923902
C	-0.473715	0.852427	4.404181
C	-0.757708	2.524405	1.874612
C	0.869537	0.725938	-0.706695
C	2.448191	-1.631950	0.294902
H	5.446599	-4.539239	2.577872
H	4.883841	-3.454269	1.282523
H	3.877640	-3.717147	2.728923
H	8.010710	-3.182823	2.805230
H	8.209496	-1.453534	3.167999
H	7.650417	-1.965289	1.557435
H	6.421740	-3.434874	5.275963
H	4.794026	-2.741984	5.456088
H	6.232861	-1.709497	5.670624
H	7.315999	1.934545	4.963853
H	7.427647	0.161775	4.912017
H	5.986426	0.939134	5.620569
H	4.051721	2.648685	3.804917
H	5.507167	3.485653	3.205809
H	4.374627	2.737049	2.060445
H	7.705570	2.201780	1.909538
H	6.719441	1.233509	0.790910
H	7.943003	0.440585	1.807043
H	3.399140	-1.170018	-0.016033
H	2.675133	-2.361852	1.088337
H	2.058087	-2.200311	-0.565104
H	-1.231962	-0.589490	1.519330
H	-0.230786	-1.912552	2.159152
H	-0.718973	-1.912656	0.447253
H	0.608915	0.085593	-1.565333
H	1.777546	1.290369	-0.974774
H	0.054304	1.449361	-0.564433
H	-1.457986	3.177576	2.420769
H	-1.354121	1.754583	1.361265
H	-0.261844	3.136473	1.104309
H	0.525471	3.954484	4.334317
H	1.911090	3.869624	3.214740
H	1.951374	2.975599	4.753416
H	-0.928644	-0.062730	3.994163
H	-1.283532	1.481377	4.808086
H	0.169427	0.552751	5.247741
H	4.646865	-0.548237	1.789913
H	2.594419	1.342240	1.887158
H	3.101307	0.967819	4.931233
H	3.351777	0.940199	5.773251
C	4.210455	-0.336526	7.746752
N	3.880225	0.956242	7.189105
C	4.360899	2.036605	7.714327
N	5.190330	2.054902	8.791364
C	5.706669	0.811660	9.357555
C	5.602397	-0.331507	8.362404
C	5.638785	3.295178	9.411797
C	6.651686	4.084160	8.579608
C	6.020009	4.907341	7.458546
C	5.188970	4.108537	6.456560
C	4.000861	3.349251	7.062448
H	6.085390	3.016097	10.376152
H	4.771904	3.931357	9.657398
H	7.392030	3.376305	8.169133
H	7.202516	4.761706	9.251557
H	6.812145	5.449095	6.918130
H	5.376797	5.682262	7.913391
H	5.156687	0.569425	10.285317
H	6.759273	0.973253	9.638457
H	3.275885	3.110735	6.276471
H	3.469449	3.988378	7.787834
H	5.825590	3.395103	5.910955
H	4.793444	4.799834	5.696952
H	5.821361	-1.285517	8.862164
H	6.350813	-0.197337	7.565672
H	4.138986	-1.085585	6.943741

H	3.456174	-0.617247	8.503912

86

int_1^{DBU}

C	-0.322498	1.412729	5.070267
C	0.948809	2.183615	4.758677
N	2.049430	1.249899	4.621612
C	2.092652	0.076092	5.203991
N	1.129031	-0.332980	6.033506
C	-0.080451	0.468980	6.236968
C	3.245404	-0.825493	4.868322
C	2.813656	-2.087200	4.106369
C	2.245123	-3.200145	4.983764
C	1.001997	-2.825841	5.790701
C	1.199485	-1.626374	6.717925
C	0.427889	1.062620	1.397945
Si	1.586989	2.125796	0.327430
C	0.567867	2.749444	-1.144387
C	1.971200	3.698765	1.311937
C	3.075073	1.139575	-0.198133
Si	2.767427	-0.537190	-0.966564
C	2.368253	-1.857579	0.334561
Sn	4.695279	0.887179	1.411276
C	5.544898	3.010535	1.390306
Si	6.197875	3.503912	-0.291901
C	7.613751	4.766510	-0.255221
Si	6.686559	3.164275	2.857103
C	5.873545	2.487986	4.438191
C	8.282992	2.188193	2.591522
C	7.131434	4.959316	3.266963
C	4.309576	-1.098082	-1.902172
C	1.333902	-0.576151	-2.207005
C	4.833152	4.343398	-1.301642
C	6.852131	2.012964	-1.254504
H	2.562962	4.399791	0.702728
H	2.545910	3.490956	2.226669
H	1.032244	4.210328	1.585349
H	-0.278150	3.369515	-0.803802
H	0.164227	1.930174	-1.756663
H	1.194589	3.376660	-1.799768
H	-0.388850	1.671629	1.821014
H	0.985507	0.590522	2.223108
H	-0.033515	0.259038	0.802113
H	1.473655	-1.590816	0.919421
H	3.212518	-1.982514	1.030921
H	2.178730	-2.829969	-0.148580
H	4.161228	-2.091388	-2.356054
H	4.553937	-0.391721	-2.712433
H	5.182204	-1.156281	-1.232099
H	1.281834	-1.561256	-2.699479
H	1.459784	0.187042	-2.991662
H	0.362672	-0.397042	-1.718063
H	5.732252	1.396239	4.383102
H	6.502487	2.702972	5.317712
H	4.885534	2.949169	4.601946
H	8.849288	2.565758	1.725493
H	8.940405	2.247684	3.473960
H	8.056869	1.126065	2.403786
H	7.705601	5.013500	4.206805
H	6.220034	5.566512	3.394198
H	7.735765	5.426270	2.475676
H	7.943674	4.985666	-1.284227
H	8.490448	4.404711	0.304711
H	7.290895	5.715851	0.201190
H	5.202213	4.623076	-2.301828
H	4.503817	5.267200	-0.797567
H	3.948277	3.703697	-1.434415
H	7.635168	1.491496	-0.680001
H	7.290381	2.327202	-2.215605
H	6.060177	1.278783	-1.472618
H	3.583131	1.726022	-0.985990
H	4.686856	3.674811	1.594970
H	3.510051	1.416623	2.723806
H	2.753912	1.409285	3.834370
H	0.418501	-1.607085	7.489398

H	2.157425	-1.706006	7.255958
H	0.152559	-2.613691	5.119057
H	0.703750	-3.687985	6.407533
H	2.012933	-4.070621	4.351501
H	3.030398	-3.533465	5.685468
H	0.009816	1.026746	7.185164
H	-0.926831	-0.224708	6.343380
H	3.778788	-1.090640	5.795640
H	3.945081	-0.248851	4.249758
H	2.096554	-1.803968	3.318688
H	3.697943	-2.464756	3.572830
H	-1.144753	2.098692	5.313907
H	-0.619823	0.837501	4.179785
H	0.865205	2.736930	3.813633
H	1.179205	2.912755	5.552999

86

TS_2^{DBU}

C	-0.100361	0.164925	0.344483
C	-0.237207	-0.154876	1.822436
N	0.696548	-1.212484	2.166725
C	1.132593	-2.122599	1.321877
N	0.687539	-2.163318	0.067459
C	-0.186540	-1.114387	-0.470642
C	2.155055	-3.106050	1.816689
C	3.502954	-3.050667	1.084329
C	3.531037	-3.717293	-0.288013
C	2.545656	-3.144066	-1.304692
C	1.086980	-3.227998	-0.856580
C	2.215991	3.496992	2.136074
Si	3.475378	3.316796	3.543587
C	4.693206	4.765050	3.420053
C	2.536386	3.592064	5.157581
C	4.363538	1.673933	3.427490
Si	5.028323	1.178382	1.758766
C	3.631765	0.665964	0.569542
Sn	3.200498	-0.088135	4.287619
C	3.325383	0.323639	6.509855
Si	5.094197	0.519047	7.094045
C	5.364754	0.099599	8.924341
Si	2.283765	-0.972883	7.363829
C	0.603763	-1.197605	6.508550
C	3.143942	-2.658055	7.350229
C	1.868885	-0.515371	9.153217
C	6.211206	-0.288224	1.927468
C	5.986179	2.539739	0.855861
C	5.670546	2.314066	6.929951
C	6.274176	-0.594388	6.119443
H	3.208422	3.532402	6.026795
H	1.738959	2.845043	5.286798
H	2.079111	4.595343	5.153210
H	4.165716	5.729591	3.504887
H	5.253684	4.766458	2.473483
H	5.427173	4.716556	4.241477
H	1.579336	4.381622	2.297692
H	1.562564	2.610333	2.094450
H	2.707251	3.613544	1.156747
H	3.082128	1.548682	0.208673
H	2.915607	0.008044	1.084533
H	4.032607	0.131463	-0.307890
H	6.570641	-0.631093	0.942991
H	7.093418	-0.001162	2.522895
H	5.728796	-1.135107	2.440379
H	6.432579	2.151428	-0.074630
H	6.802172	2.934201	1.482422
H	5.333382	3.385575	0.587374
H	0.710752	-1.637879	5.503714
H	-0.042327	-1.865893	7.101247
H	0.088192	-0.230625	6.394847
H	4.093367	-2.637456	7.908102
H	2.505305	-3.433724	7.803080
H	3.372035	-2.960952	6.315315
H	1.171099	-1.249130	9.589610
H	1.384860	0.474099	9.196337
H	2.763155	-0.478495	9.791990

H	6.425006	0.250157	9.186928
H	5.107636	-0.943803	9.165003
H	4.763481	0.753604	9.575984
H	6.718579	2.412174	7.256980
H	5.059526	2.971318	7.570314
H	5.599881	2.694316	5.900790
H	5.955816	-1.647668	6.180650
H	7.298128	-0.524610	6.521017
H	6.311723	-0.323949	5.051951
H	5.255769	1.740035	4.077734
H	2.819998	1.291541	6.674968
H	1.577954	0.725895	4.073081
H	1.241033	-1.087014	3.036532
H	0.414678	-3.148387	-1.720765
H	0.879648	-4.207453	-0.396291
H	2.785830	-2.090522	-1.526863
H	2.643944	-3.696673	-2.252034
H	4.550818	-3.646192	-0.696120
H	3.323902	-4.796153	-0.169760
H	-1.221441	-1.496579	-0.503561
H	0.127640	-0.923854	-1.507008
H	1.718830	-4.118059	1.762457
H	2.330204	-2.872428	2.876708
H	3.826759	-2.002335	1.015035
H	4.240059	-3.541218	1.736700
H	-0.888350	0.859007	0.023783
H	0.868352	0.654736	0.168533
H	0.014295	0.705389	2.458576
H	-1.261851	-0.475941	2.071901

86

int_2^{DBU}

C	0.479142	0.196759	-0.072660
C	0.134878	0.080794	1.402349
N	1.027421	-0.877095	2.016913
C	1.493267	-1.921319	1.380710
N	1.142574	-2.177408	0.113886
C	0.428944	-1.186919	-0.698813
C	2.455242	-2.818365	2.107542
C	3.811195	-3.009271	1.412618
C	3.822792	-3.993668	0.246490
C	2.928006	-3.607633	-0.927917
C	1.460045	-3.447164	-0.540459
C	3.581420	5.099361	4.323220
Si	4.692186	3.660798	4.846061
C	6.497198	4.209346	4.654054
C	4.426049	3.413811	6.699019
C	4.347274	2.137691	3.804929
Si	4.181607	2.408857	1.968331
C	2.523246	3.185536	1.481300
Sn	2.588405	0.901761	4.531192
C	3.418670	-0.069564	6.404934
Si	5.069715	-0.905147	6.140846
C	5.469108	-2.279014	7.386616
Si	2.047021	-1.110782	7.136223
C	0.404724	-0.173219	7.181147
C	1.763955	-2.678741	6.105419
C	2.379527	-1.626927	8.926911
C	4.291640	0.741253	1.065806
C	5.530924	3.501558	1.214383
C	6.513715	0.305030	6.287978
C	5.153858	-1.750805	4.445152
H	5.067768	2.615345	7.099589
H	3.378648	3.158138	6.920052
H	4.674170	4.345717	7.233230
H	6.702102	5.097334	5.274652
H	6.749287	4.458969	3.612553
H	7.183738	3.411546	4.982118
H	3.710646	5.951368	5.009837
H	2.521816	4.798851	4.352579
H	3.811695	5.454509	3.305983
H	2.463269	4.235074	1.805249
H	1.678402	2.655478	1.948270
H	2.386704	3.163906	0.387116
H	4.207639	0.861603	-0.027433

H	5.250337	0.240855	1.279867
H	3.482854	0.081258	1.412647
H	5.429772	3.548135	0.117441
H	6.535345	3.112343	1.446378
H	5.476075	4.532221	1.598962
H	0.034109	0.068490	6.172448
H	-0.366236	-0.772104	7.693233
H	0.514372	0.778768	7.724776
H	2.677234	-3.289175	6.018880
H	0.979385	-3.308845	6.554569
H	1.435698	-2.404817	5.089346
H	1.490087	-2.118152	9.354992
H	2.601278	-0.741964	9.545587
H	3.226798	-2.321021	9.020500
H	6.463706	-2.702134	7.168236
H	4.740691	-3.104663	7.352959
H	5.490052	-1.891827	8.417731
H	7.467222	-0.216916	6.105526
H	6.556310	0.727262	7.305220
H	6.445161	1.144851	5.582830
H	4.408976	-2.562405	4.408036
H	6.146837	-2.198656	4.277459
H	4.944792	-1.056406	3.615593
H	5.218613	1.464762	3.912093
H	3.588639	0.759502	7.115736
H	1.724232	2.220143	5.329561
H	1.519266	-0.522368	2.916565
H	0.821669	-3.497404	-1.432897
H	1.142791	-4.279542	0.108490
H	3.277668	-2.668645	-1.389779
H	2.994718	-4.384703	-1.705447
H	4.857691	-4.107792	-0.110780
H	3.514912	-4.989544	0.612685
H	-0.615476	-1.518396	-0.832374
H	0.899029	-1.174667	-1.694080
H	1.965335	-3.792026	2.281211
H	2.611527	-2.370195	3.098428
H	4.187387	-2.026778	1.088145
H	4.517233	-3.358311	2.179097
H	-0.219696	0.864239	-0.594370
H	1.488201	0.626392	-0.170621
H	0.268900	1.031256	1.939685
H	-0.911284	-0.238368	1.543515

86

TS_3^{DBU}

C	-0.025614	-0.012264	-0.046487
C	-0.029385	0.014664	1.474583
N	0.946836	-0.913304	1.992047
C	1.172947	-2.036719	1.379638
N	0.533785	-2.395556	0.240626
C	-0.279628	-1.437490	-0.506567
C	2.209710	-2.959833	1.965283
C	3.355867	-3.354569	1.024007
C	3.031846	-4.449339	0.010869
C	1.924720	-4.097913	-0.978602
C	0.600210	-3.747859	-0.302600
C	4.187164	5.101405	2.506433
Si	5.280182	3.669041	3.075092
C	7.053372	3.999818	2.501490
C	5.312090	3.731511	4.962174
C	4.666268	2.031731	2.380028
Si	4.154920	2.025367	0.579770
C	2.479267	2.856635	0.296345
Sn	3.053742	1.094987	3.607446
C	4.067651	0.322388	5.442556
Si	5.603750	-0.683556	5.060198
C	6.100326	-1.890333	6.432593
Si	2.753656	-0.476666	6.518834
C	1.222175	0.623184	6.663624
C	2.183516	-2.123003	5.777948
C	3.345330	-0.767489	8.290956
C	3.993284	0.234792	-0.016131
C	5.402279	2.872393	-0.560973
C	7.110928	0.425589	4.803713

C	5.369820	-1.754801	3.515996
H	5.953477	2.945566	5.386931
H	4.302194	3.617801	5.385458
H	5.711008	4.705283	5.290262
H	7.423058	4.953798	2.911861
H	7.132585	4.049525	1.405245
H	7.731747	3.204231	2.850629
H	4.500001	6.035881	2.999221
H	3.134147	4.916213	2.772468
H	4.243436	5.264136	1.418519
H	2.520458	3.935120	0.507534
H	1.698900	2.426330	0.943493
H	2.160127	2.731043	-0.751499
H	3.703991	0.184031	-1.078882
H	4.944181	-0.309931	0.102840
H	3.228049	-0.287635	0.577192
H	5.101084	2.762122	-1.615595
H	6.406701	2.433598	-0.448421
H	5.482398	3.949905	-0.347609
H	0.699768	0.743540	5.701336
H	0.508071	0.188475	7.381985
H	1.491148	1.630773	7.019040
H	3.021880	-2.815446	5.599788
H	1.469312	-2.626572	6.449093
H	1.672426	-1.945805	4.817716
H	2.504686	-1.100007	8.921825
H	3.737539	0.166924	8.724488
H	4.137661	-1.526315	8.357744
H	7.018062	-2.427121	6.140854
H	5.321698	-2.644116	6.629832
H	6.306904	-1.363964	7.377838
H	7.997125	-0.186499	4.570056
H	7.332966	0.994387	5.721239
H	6.974630	1.149463	3.988030
H	4.612014	-2.529344	3.713514
H	6.309439	-2.268256	3.255655
H	5.043190	-1.178123	2.635743
H	5.511974	1.320924	2.437544
H	4.405199	1.221413	5.990547
H	2.289961	2.537779	4.240791
H	1.860307	-0.262285	2.711865
H	-0.224098	-3.830625	-1.024678
H	0.377909	-4.478076	0.493504
H	2.230314	-3.250402	-1.615621
H	1.755992	-4.953898	-1.651322
H	3.947659	-4.703007	-0.545485
H	2.740971	-5.366460	0.554424
H	-1.347501	-1.696647	-0.390241
H	-0.034428	-1.544087	-1.575472
H	1.704747	-3.860769	2.355691
H	2.616150	-2.437369	2.839873
H	3.719893	-2.454680	0.504687
H	4.191942	-3.694227	1.652749
H	-0.789825	0.656515	-0.466478
H	0.954673	0.336301	-0.407132
H	0.228586	1.013376	1.866681
H	-1.031068	-0.235593	1.865802

32

PMP

C	-6.110731	-1.208278	1.108526
C	-4.974141	-0.183014	1.272355
N	-4.206691	-0.087638	0.009066
C	-4.918059	0.038245	-1.283979
C	-6.056654	-0.995717	-1.348364
C	-6.960336	-0.978368	-0.128509
C	-3.009594	0.719185	0.105166
C	-4.055216	-0.731059	2.379994
C	-5.550879	1.163697	1.762765
C	-3.949456	-0.314530	-2.428092
C	-5.478483	1.451196	-1.560148
H	-5.658302	-2.212398	1.042034
H	-6.727916	-1.194765	2.021085
H	-6.633511	-0.823401	-2.271085
H	-5.603722	-1.998018	-1.435607

H	-7.730357	-1.761064	-0.213208
H	-7.503398	-0.021458	-0.057635
H	-2.369917	0.567869	-0.772855
H	-2.409957	0.418329	0.972851
H	-3.188705	1.811992	0.194640
H	-3.481533	-1.591256	2.004089
H	-3.351884	0.021433	2.764603
H	-4.664714	-1.064188	3.233392
H	-5.947683	1.056516	2.784076
H	-4.774413	1.942616	1.791098
H	-6.369043	1.530124	1.129201
H	-3.389152	-1.228414	-2.180665
H	-4.519714	-0.494215	-3.351812
H	-3.233122	0.490452	-2.646588
H	-5.828783	1.523048	-2.601377
H	-6.325900	1.705191	-0.910186
H	-4.705199	2.220928	-1.418769

33

PMPH^{+}

C	-6.960000	-0.967062	-0.134278
C	-6.118463	-1.218389	1.105670
C	-4.986208	-0.203516	1.320973
N	-4.168236	-0.120667	-0.003641
C	-4.933416	0.034018	-1.353230
C	-6.069466	-0.999023	-1.365071
C	-4.044253	-0.747695	2.401941
C	-5.520579	1.161657	1.747784
C	-2.994756	0.790629	0.100180
C	-3.947481	-0.313898	-2.475181
C	-5.456234	1.454619	-1.553258
H	-5.681385	-2.231064	1.046294
H	-6.737789	-1.209747	2.014230
H	-6.652584	-0.828008	-2.281514
H	-5.631550	-2.007827	-1.468054
H	-7.732192	-1.744242	-0.218634
H	-7.500740	-0.011073	-0.060069
H	-2.369580	0.683103	-0.790134
H	-2.402786	0.524736	0.979869
H	-3.333370	1.826464	0.186440
H	-3.492822	-1.639796	2.063336
H	-3.328101	-0.001464	2.770548
H	-4.652690	-1.057120	3.262468
H	-5.961613	1.044923	2.747165
H	-4.730179	1.918539	1.838095
H	-6.306682	1.549008	1.091577
H	-3.405329	-1.252683	-2.276936
H	-4.520474	-0.466373	-3.399779
H	-3.221253	0.484011	-2.678919
H	-5.851136	1.519307	-2.576388
H	-6.273740	1.719585	-0.874930
H	-4.667349	2.213946	-1.470685
H	-3.775518	-1.065696	-0.079873

91

TS$_1$^{PMP}

C	0.082751	0.002136	-0.125967
C	0.047723	-0.045719	1.393556
C	0.221495	1.322498	2.078084
N	1.446867	1.989513	1.496253
C	1.558805	2.084944	-0.008724
C	1.364589	0.675420	-0.592123
C	0.467242	1.054482	3.572478
C	-1.049446	2.175188	1.951544
C	1.844578	3.223765	2.179255
C	2.975953	2.562619	-0.357526
C	0.563341	3.067832	-0.647545
C	2.694669	-1.248446	5.546634
Si	4.343735	-0.355611	5.287527
C	5.308275	-0.516830	6.908147
C	5.269511	-1.025118	3.803186
Si	6.166603	-2.656246	4.066983
C	5.088807	-3.906650	4.985331
Sn	3.949702	-1.267860	1.978917
C	5.259937	-0.445903	0.316908

Si	6.668686	0.720200	0.722224
C	7.303261	1.585374	-0.841801
Si	5.529494	-1.635201	-1.118144
C	4.376836	-3.133635	-1.062978
C	7.291048	-2.300581	-1.265474
C	5.118744	-0.724373	-2.727689
C	4.011720	1.497739	5.086155
C	8.159238	-0.119461	1.520625
C	6.105624	2.120641	1.867644
C	7.777795	-2.417393	5.023100
C	6.630600	-3.452185	2.413153
H	7.359196	-2.967980	-2.139821
H	7.583154	-2.883739	-0.378902
H	8.029981	-1.496093	-1.403069
H	4.515154	-3.742728	-1.971378
H	3.318284	-2.831141	-1.016685
H	4.571473	-3.782481	-0.194296
H	5.243300	-1.380722	-3.604256
H	5.761203	0.158140	-2.869740
H	4.071361	-0.378351	-2.716065
H	8.071394	2.333834	-0.586594
H	6.484169	2.109752	-1.361130
H	7.751980	0.877982	-1.556852
H	8.963677	0.619895	1.664911
H	8.558030	-0.927276	0.889142
H	7.920890	-0.548361	2.504096
H	6.978897	2.690418	2.224943
H	5.555637	1.753347	2.745363
H	5.452396	2.829756	1.334060
H	4.747307	-0.050770	7.734868
H	6.285823	-0.012898	6.839666
H	5.490605	-1.568099	7.180710
H	3.370242	1.869873	5.901781
H	3.524863	1.727686	4.127655
H	4.957593	2.062296	5.120392
H	2.089416	-0.730220	6.308264
H	2.860662	-2.279067	5.895122
H	2.096520	-1.314293	4.624307
H	5.594186	-4.884799	5.029019
H	4.124606	-4.048756	4.470860
H	4.876713	-3.594672	6.019848
H	8.304669	-3.378803	5.137771
H	7.601394	-2.005019	6.027503
H	8.453929	-1.728695	4.491589
H	7.241327	-4.354868	2.575353
H	7.213122	-2.756782	1.791822
H	5.743826	-3.761855	1.834711
H	4.503347	0.228545	-0.130212
H	6.049540	-0.282355	3.561337
H	3.230629	0.634107	2.156096
H	0.853049	-0.713862	1.742557
H	-0.898157	-0.482436	1.749216
H	1.392100	0.753633	-1.689867
H	2.224838	0.049585	-0.302632
H	0.034204	-1.021156	-0.526909
H	-0.800827	0.527641	-0.523994
H	2.924355	3.378786	2.055930
H	1.658289	3.134373	3.252862
H	1.311687	4.110785	1.805717
H	1.469031	0.645285	3.753078
H	0.335940	1.949721	4.196451
H	-0.262509	0.310806	3.922235
H	-1.825053	1.751588	2.606219
H	-0.876281	3.211567	2.276904
H	-1.458785	2.198523	0.935501
H	3.734949	2.027988	0.226171
H	3.169889	2.363232	-1.420978
H	3.106318	3.642111	-0.195872
H	0.881822	3.259623	-1.682445
H	-0.461486	2.683763	-0.690165
H	0.544019	4.036951	-0.129611
H	2.475101	1.145540	1.852153

91

int$_1^{PMP}$

C	1.387584	-0.830093	8.044423
C	2.860677	-1.201795	7.969233
C	3.830242	-0.048574	8.278152
N	3.424788	1.145018	7.403043
C	1.953537	1.585765	7.418955
C	1.099745	0.343913	7.120211
C	5.236903	-0.491057	7.850773
C	3.851444	0.302714	9.767006
C	4.368922	2.282164	7.460454
C	1.763720	2.602625	6.287596
C	1.534415	2.237284	8.740131
C	6.686832	-2.149617	4.855303
Si	6.688577	-0.683614	3.653639
C	8.283323	-0.831997	2.641778
C	5.134787	-0.630999	2.618989
Si	5.067596	-1.825053	1.175223
C	5.639467	-3.555188	1.678854
Sn	3.264935	-0.918965	3.892677
C	1.829354	0.637239	3.012978
Si	2.531245	2.125272	2.126473
C	1.218046	3.481471	1.916447
Si	0.205748	-0.048274	2.363008
C	-0.106139	-1.843657	2.866994
C	-0.000506	0.040220	0.486311
C	-1.210744	0.953852	3.133027
C	6.888206	0.917242	4.651722
C	3.204636	1.762130	0.400072
C	3.921534	2.947345	3.122161
C	6.132638	-1.232824	-0.271818
C	3.309529	-2.006370	0.501274
H	-0.988066	-0.360247	0.205148
H	0.761893	-0.557566	-0.035853
H	0.059212	1.073203	0.109608
H	-1.109646	-2.158145	2.535804
H	-0.051141	-1.971879	3.959978
H	0.628565	-2.533243	2.422884
H	-2.194642	0.585776	2.799162
H	-1.140459	2.021119	2.871351
H	-1.182542	0.875870	4.233459
H	1.659867	4.389218	1.473170
H	0.781788	3.761954	2.889731
H	0.391228	3.160232	1.263332
H	3.538861	2.701446	-0.069800
H	2.436560	1.318073	-0.250508
H	4.062783	1.075766	0.422792
H	4.425394	3.711928	2.508486
H	4.676038	2.222579	3.458967
H	3.522224	3.460223	4.012739
H	9.164351	-0.794136	3.303709
H	8.371276	-0.006744	1.916816
H	8.329257	-1.777997	2.079737
H	7.728190	0.838993	5.361703
H	5.970431	1.155372	5.208814
H	7.095423	1.764629	3.978154
H	7.502091	-2.055833	5.591611
H	6.835944	-3.093563	4.309141
H	5.735910	-2.247501	5.402522
H	5.483563	-4.266452	0.851809
H	5.066237	-3.916813	2.547907
H	6.708385	-3.580624	1.943231
H	6.055207	-1.929678	-1.122534
H	7.194406	-1.151085	0.004407
H	5.800717	-0.242138	-0.622612
H	3.303426	-2.629565	-0.407561
H	2.887769	-1.024533	0.242492
H	2.635714	-2.480909	1.232998
H	1.491463	1.066096	3.976928
H	5.086782	0.386017	2.191258
H	3.825670	0.397037	5.131025
H	3.077577	-1.575235	6.953619
H	3.094552	-2.022319	8.664240
H	0.043135	0.645071	7.180745
H	1.277441	0.033900	6.077130
H	0.780840	-1.693868	7.736652
H	1.087764	-0.599073	9.079857

H	4.270391	2.870197	6.541646
H	5.393739	1.905686	7.500067
H	4.178000	2.916400	8.333229
H	5.339300	-0.518510	6.757943
H	6.032167	0.141519	8.269133
H	5.406751	-1.510625	8.223013
H	4.377791	-0.503472	10.297227
H	4.402001	1.233788	9.964498
H	2.854197	0.387217	10.211525
H	2.251243	2.267929	5.362849
H	0.689306	2.700250	6.079199
H	2.141176	3.600825	6.550570
H	0.567984	2.733429	8.573401
H	1.393956	1.521564	9.556593
H	2.238893	3.011360	9.074112
H	3.558532	0.760751	6.386586

33

TBTMG

N	-2.334837	0.988610	-0.393274
C	-2.126394	-0.221590	-0.047231
N	-0.812450	-0.684503	-0.000366
N	-3.070284	-1.188076	0.330958
C	-3.498349	1.815214	-0.100005
C	-0.478859	-1.994617	-0.513629
C	0.247458	0.289766	-0.107281
C	-2.919976	-1.951659	1.541851
C	-4.182202	-1.525407	-0.514416
C	-2.925675	3.226983	0.125306
C	-4.437946	1.869951	-1.314774
C	-4.278076	1.414786	1.165452
H	0.316161	-2.460584	0.091353
H	-0.121137	-1.942486	-1.560513
H	-1.356552	-2.650840	-0.490583
H	-0.007992	1.182094	0.476265
H	1.181260	-0.148640	0.278505
H	0.414418	0.617287	-1.150609
H	-2.014282	-1.629853	2.071177
H	-3.786129	-1.801906	2.213934
H	-2.834862	-3.038772	1.346285
H	-5.162623	-1.243468	-0.082702
H	-4.080106	-1.023037	-1.484324
H	-4.207451	-2.616313	-0.698876
H	-3.724669	3.962805	0.309452
H	-2.246380	3.226424	0.991535
H	-2.347145	3.541281	-0.755253
H	-5.206415	2.646255	-1.175720
H	-4.955840	0.914608	-1.475211
H	-3.864298	2.108327	-2.222665
H	-4.825444	0.471018	1.051147
H	-3.592958	1.310098	2.021363
H	-5.011503	2.197707	1.413438

34

TBTMGH^+

C	-0.327844	-1.978453	-0.255715
N	-0.846251	-0.626796	-0.076581
C	0.159121	0.416786	0.079310
C	-2.155827	-0.341382	-0.190159
N	-3.071130	-1.264683	0.156067
C	-4.310181	-1.443355	-0.586054
N	-2.520831	0.855232	-0.678355
C	-3.534713	1.831263	-0.163371
C	-4.003296	1.441335	1.239161
C	-2.883620	-2.147307	1.299190
C	-2.795632	3.174962	-0.090106
C	-4.709403	1.949961	-1.137945
H	0.013641	-2.412968	0.695859
H	0.525971	-1.940192	-0.947400
H	-1.095310	-2.625085	-0.694910
H	-0.290025	1.321222	0.505904
H	0.935522	0.060557	0.770343
H	0.641957	0.664842	-0.880177
H	-2.037765	-1.808059	1.907792
H	-3.790063	-2.113148	1.922161

H	-2.714876	-3.190738	0.991383
H	-5.187210	-1.111185	-0.008964
H	-4.266003	-0.889012	-1.529604
H	-4.433748	-2.512829	-0.812620
H	-3.482479	3.965368	0.241566
H	-1.958723	3.135302	0.623454
H	-2.407014	3.472906	-1.077035
H	-5.373110	2.767252	-0.822407
H	-5.310775	1.033288	-1.177235
H	-4.355086	2.178133	-2.153903
H	-4.609420	0.527229	1.250289
H	-3.152256	1.310669	1.925240
H	-4.630480	2.250303	1.637999
H	-1.825600	1.290359	-1.275452

92

TS_1^{TBTMG}

C	-3.841983	-3.438623	1.632834
Si	-2.401107	-2.660933	0.683161
C	-0.844233	-3.050915	1.680051
C	-2.592669	-0.808388	0.436125
Sn	-1.060945	0.026027	-0.991929
C	-0.830674	2.049623	0.001379
Si	0.068641	2.146260	1.640355
C	1.941093	2.188129	1.377945
C	-2.242921	-3.578540	-0.961778
Si	-4.317282	-0.245959	-0.065231
C	-5.479690	-0.233166	1.423558
C	-5.051110	-1.342030	-1.417826
C	-4.348051	1.518311	-0.754117
Si	-0.274164	3.267445	-1.317431
C	-1.606919	3.430080	-2.647101
C	1.313715	2.686182	-2.168471
C	0.053989	5.013713	-0.666858
C	-0.419289	3.700627	2.603861
C	-0.317972	0.718030	2.819239
H	-3.644656	-4.508878	1.807887
H	-4.794722	-3.361580	1.086187
H	-3.977830	-2.957099	2.614636
H	-3.126515	-3.437537	-1.601452
H	-2.126623	-4.659422	-0.780158
H	-1.364605	-3.240867	-1.534717
H	-0.774406	-4.133977	1.872147
H	-0.848412	-2.534325	2.653463
H	0.059273	-2.743859	1.133837
H	-5.376960	1.771988	-1.057224
H	-3.708067	1.647326	-1.642239
H	-4.032568	2.260299	-0.003602
H	-6.473051	0.148940	1.136698
H	-5.083861	0.424638	2.214524
H	-5.613270	-1.234932	1.856921
H	-5.207659	-2.377976	-1.079850
H	-4.394599	-1.372435	-2.302594
H	-6.027435	-0.944608	-1.738535
H	-1.366293	0.757760	3.156336
H	0.317552	0.793406	3.717517
H	-0.151875	-0.266054	2.358658
H	2.273918	1.297139	0.823576
H	2.465672	2.215331	2.345213
H	2.247486	3.079851	0.809301
H	0.054747	3.701528	3.599386
H	-1.510637	3.731230	2.756651
H	-0.128400	4.628001	2.090268
H	0.284023	5.689238	-1.507012
H	0.907547	5.045381	0.028483
H	-0.824576	5.419099	-0.140163
H	-1.291384	4.139588	-3.429119
H	-2.552300	3.799279	-2.218185
H	-1.813297	2.465154	-3.138823
H	2.111021	2.473279	-1.440217
H	1.683408	3.460982	-2.859421
H	1.140083	1.770839	-2.755095
H	-2.398061	-0.322969	1.409479
H	-1.881494	2.300962	0.240560
H	0.483640	-0.679782	0.261939

C	2.553189	-2.000896	2.461684
N	3.500700	-1.046401	1.930052
C	4.057755	-0.120841	2.891727
C	3.696850	-0.924210	0.579624
N	4.904016	-0.358317	0.212289
C	6.154160	-0.766788	0.807098
N	2.765171	-1.317702	-0.233225
C	2.940175	-1.923363	-1.561220
C	2.603440	-0.939721	-2.690262
C	4.976011	0.671548	-0.795222
C	1.913594	-3.070724	-1.602447
C	4.337003	-2.526205	-1.773507
H	2.976698	-2.475935	3.361389
H	2.344052	-2.769467	1.709426
H	1.599286	-1.524169	2.738446
H	4.517623	0.730785	2.378005
H	3.255716	0.267152	3.540251
H	4.815170	-0.602167	3.534864
H	5.466475	0.330014	-1.725127
H	3.967105	1.022492	-1.042205
H	5.551297	1.529976	-0.407601
H	5.986469	-1.604103	1.495747
H	6.854598	-1.106367	0.023443
H	6.641991	0.052733	1.365256
H	1.935015	-3.586662	-2.574334
H	2.124024	-3.806673	-0.811555
H	0.896183	-2.683957	-1.446282
H	5.120181	-1.764533	-1.877733
H	4.337903	-3.129031	-2.693889
H	4.608005	-3.188858	-0.936402
H	2.615639	-1.459454	-3.660246
H	1.596056	-0.521818	-2.548549
H	3.318026	-0.108943	-2.747398
H	1.298915	-0.926594	0.081987

92

int$_1^{TBTMG}$

C	-4.065832	-3.383691	1.747517
Si	-2.498579	-2.625503	0.996330
C	-2.065584	-3.683244	-0.513125
C	-1.130695	-2.913711	2.274444
C	-2.658699	-0.814944	0.565652
Si	-4.279315	-0.301928	-0.219623
C	-4.232808	1.449122	-0.940109
Sn	-0.915706	-0.085529	-0.712000
C	-0.812336	2.018402	0.207709
Si	-0.051487	3.156708	-1.061588
C	0.203351	4.939751	-0.467027
Si	-0.159933	2.156292	1.944179
C	-0.659761	0.753567	3.109547
C	1.738900	2.182028	1.943988
C	-0.743899	3.740460	2.805741
C	-5.694642	-0.276741	1.039115
C	-4.756025	-1.457418	-1.638955
C	-1.138065	3.258950	-2.602318
C	1.664983	2.542670	-1.600270
C	4.292765	-0.022075	2.533272
N	3.600870	-0.882110	1.586586
C	3.758077	-0.755494	0.255835
N	2.770883	-1.098426	-0.565345
C	2.845960	-1.891681	-1.824614
C	4.223340	-2.536401	-2.000291
C	2.600296	-1.765760	2.169201
N	4.931180	-0.258334	-0.214738
C	4.964285	0.692314	-1.310759
C	6.212039	-0.650578	0.336445
C	2.472095	-1.032375	-3.035228
C	1.798674	-3.002969	-1.660614
H	-3.879226	-4.428653	2.045809
H	-4.905838	-3.385677	1.035005
H	-4.388172	-2.829280	2.643637
H	-2.804943	-3.562742	-1.319022
H	-2.031345	-4.750307	-0.238288
H	-1.082818	-3.410381	-0.927412
H	-1.026720	-3.988155	2.498040

H	-1.346182	-2.387700	3.218123
H	-0.171200	-2.540180	1.886969
H	-5.202027	1.681433	-1.411162
H	-3.451362	1.568953	-1.706997
H	-4.055656	2.203776	-0.156834
H	-6.620085	0.102354	0.574824
H	-5.446942	0.390080	1.881530
H	-5.907135	-1.272975	1.453562
H	-4.957351	-2.481799	-1.287330
H	-3.946560	-1.508436	-2.385250
H	-5.663610	-1.093908	-2.147205
H	-1.741808	0.765753	3.315750
H	-0.139570	0.862191	4.076142
H	-0.415669	-0.229556	2.681809
H	2.117341	1.316015	1.377973
H	2.128312	2.135523	2.973747
H	2.136954	3.096481	1.477011
H	-0.374842	3.786234	3.843924
H	-1.845461	3.767311	2.844049
H	-0.408598	4.649140	2.284455
H	0.580980	5.569624	-1.289459
H	0.927908	5.001263	0.360767
H	-0.743527	5.379654	-0.114552
H	-0.693734	3.927884	-3.357296
H	-2.135402	3.652498	-2.347353
H	-1.275405	2.266453	-3.059721
H	2.375557	2.583567	-0.760586
H	2.064653	3.165435	-2.417304
H	1.608682	1.503014	-1.958411
H	-2.582523	-0.253696	1.514060
H	-1.882484	2.293786	0.255886
H	0.387744	-0.745183	0.435946
H	3.030212	-2.249709	3.058103
H	2.307331	-2.537858	1.449062
H	1.698260	-1.204576	2.457196
H	4.678239	0.870012	2.027190
H	3.575528	0.309355	3.295567
H	5.120300	-0.549969	3.033637
H	5.408684	0.259583	-2.222477
H	3.949964	1.042422	-1.533647
H	5.571591	1.561500	-1.012420
H	6.086215	-1.491889	1.028330
H	6.871310	-0.980200	-0.483030
H	6.710911	0.177254	0.866942
H	1.766167	-3.627375	-2.564638
H	2.034005	-3.649906	-0.801587
H	0.799020	-2.569199	-1.514166
H	5.014388	-1.811672	-2.230098
H	4.172203	-3.241957	-2.841299
H	4.516094	-3.106793	-1.104522
H	2.453137	-1.656833	-3.940403
H	1.467972	-0.601832	-2.898581
H	3.188220	-0.217078	-3.201273
H	1.804815	-1.000957	-0.175970

6. References

[1] P. J. Davidson, D. H. Harris and M. F. Lappert, *J. Chem. Soc. Dalt. Trans.* 1976, **21**, 2268–2274.

[2] T. Fjeldberg, A. Haaland, B. E. R. Schilling, M. F. Lappert and A. J. Thorne, *J. Chem. Soc. Dalt. Trans.*, 1986, 1551.

[3] E. Brunner, *J. Chem. Eng. Data*, 1985, **30**, 269.

[4] K. W. Zilm, G. A. Lawless, R. M. Merrill, J. M. Millar and G. G. Webb, *J. Am. Chem. Soc.* 1987, **109**, 7236–7238.

[5] Applied radius for Sn atoms was 2.16 Å according to: A. Bondi, *J. Phys. Chem.*, 1964, **68**, 441.

[6] *Macromodel embedded in Maestro suite (Version 9.1)*; Schrödinger, Inc.: Portland, OR, 2010.

[7] For the *ω*B97X-D functional, see: (a) J.-D. Chai and M. Head-Gordon, *Phys. Chem. Chem. Phys.* 2008, **10**, 6615. (b) J.-D. Chai and M. Head-Gordon, *J. Chem. Phys.* 2008, **128**, 084106. (c) S. Grimme, *J. Comput. Chem.* 2006, **27**, 1787.

[8] For the Def2SVP and Def2TZVPP basis sets, see: F. Weigend and R. Ahlrichs, *Phys. Chem. Chem. Phys.*, 2005, **7**, 3297.

[9] J. Tomasi, B. Mennucci, E. Cancès, *J. Mol. Struct. (Theochem)* 1999, **464**, 211.

[10] A. V. Marenich, C. J. Cramer and D. G. Truhlar, *J. Phys. Chem. B,* 2009, **113**, 6378.

[11] *Gaussian 16, Revision A.03*, M. J. Frisch, G. W. Trucks, H. B. Schlegel, G. E. Scuseria, M. A. Robb, J. R. Cheeseman, G. Scalmani, V. Barone, B. Mennucci, G. A. Petersson, H. Nakatsuji, M. Caricato, X. Li, H. P. Hratchian, A. F. Izmaylov, J. Bloino, G. Zheng, J. L. Sonnenberg, M. Hada, M. Ehara, K. Toyota, R. Fukuda, J. Hasegawa, M. Ishida, T. Nakajima, Y. Honda, O. Kitao, H. Nakai, T. Vreven, J. A. Montgomery, J. E. Peralta, F. Ogliaro, M. Bearpark, J. J. Heyd, E. Brothers, K. N. Kudin, V. N. Staroverov, R. Kobayashi, J. Normand, K. Raghavachari, A. Rendell, J. C. Burant, S. S. Iyengar, J. Tomasi, M. Cossi, N. Rega, J. M. Millam, M. Klene, J. E. Knox, J. B. Cross, V. Bakken, C. Adamo, J. Jaramillo, R. Gomperts, R. E. Stratmann, O. Yazyev, A. J. Austin, R. Cammi, C. Pomelli, J. W. Ochterski, R. L. Martin, K. Morokuma, V. G. Zakrzewski, G. A. Voth, P. Salvador, J. J. Dannenberg, S. Dapprich, A. D. Daniels, O. Farkas, J. B. Foresman, J. V. Ortiz, J. Cioslowski, D. J. Fox, Gaussian, Inc., Wallingford CT, 2016.
